# Activity concentration of ^137^Cs and natural radionuclides in soil around the Belarusian nuclear power plant in the pre-commissioning period

**DOI:** 10.1093/rpd/ncag063

**Published:** 2026-06-29

**Authors:** Christian Bernhardsson, Valery Ramzaev, Aliaksandr Dvornik, Mattias Jönsson, Guillaume Pédehontaa-Hiaa, Alexander Vodovatov, Vladislav Nekrasov, Charlotta Nilsson, Kristina Eriksson Stenström

**Affiliations:** Medical Radiation Physics, Department of Translational Medicine, Lund University, Carl Bertil Laurells gata 9, 205 02 Malmö, Sweden; Saint-Petersburg Research Institute of Radiation Hygiene after Professor P.V. Ramzaev, 197101, 8 Mira Str., Saint-Petersburg, Russia; Medical Radiation Physics, Department of Translational Medicine, Lund University, Carl Bertil Laurells gata 9, 205 02 Malmö, Sweden; Medical Radiation Physics, Department of Translational Medicine, Lund University, Carl Bertil Laurells gata 9, 205 02 Malmö, Sweden; Medical Radiation Physics, Department of Translational Medicine, Lund University, Carl Bertil Laurells gata 9, 205 02 Malmö, Sweden; Division of Particle and Nuclear Physics, Department of Physics, Lund University, Professorsgatan 1BLund, SE-221 00, Sweden; Saint-Petersburg Research Institute of Radiation Hygiene after Professor P.V. Ramzaev, 197101, 8 Mira Str., Saint-Petersburg, Russia; Saint-Petersburg Research Institute of Radiation Hygiene after Professor P.V. Ramzaev, 197101, 8 Mira Str., Saint-Petersburg, Russia; Division of Particle and Nuclear Physics, Department of Physics, Lund University, Professorsgatan 1BLund, SE-221 00, Sweden; Division of Particle and Nuclear Physics, Department of Physics, Lund University, Professorsgatan 1BLund, SE-221 00, Sweden

## Abstract

In this study, soil cores (0–20 cm) were collected at 45 sites located within a 30-km radius zone around the Belarusian nuclear power plant (BelNPP) in 2019 before its commissioning. Activity of gamma-emitting radionuclides was measured in the samples using HPGe detectors. The study revealed low levels of ^137^Cs in the soil: the weighted average dry weight (d.w.) activity concentration (AC_w_) varied from 1.13 to 8.71 Bq/kg (d.w.). The AC_w_ of natural radionuclides ranged: for ^226^Ra from 7.8 to 30.8 Bq/kg (d.w.), for ^232^Th from 10.9 to 33.0 Bq/kg (d.w.), and for ^40^K from 376 to 789 Bq/kg (d.w.). A good agreement between the ambient dose equivalent rate (ADER) values calculated with the soil samples and the ADER values previously measured *in situ* at the same sites is observed. The sites characterized here now have established pre-operational levels of ^137^Cs, ^226^Ra, ^232^Th, and ^40^K in soil thus can be surveyed again in future environmental studies.

## Introduction

The two VVER-1200 reactor units of the Belarusian Nuclear Power Plant, BelNPP (Astrovets district, Grodno region, Republic of Belarus) were gradually put into operation between 2021 and 2023 [1]. Along with other man-made radionuclides, ^137^Cs may be present in the gas-aerosol emissions from the BelNPP (as well as other NPPs) during its normal operation [2–6]. To demonstrate compliance with radiation safety standards regarding the levels of emissions of technogenic radionuclides, the BelNPP has a system of radiation monitoring of the environment. The monitoring survey includes measurements of the total ambient dose equivalent rate of gamma radiation (ADER, μSv/h) in the air, the volumetric activity of ^137^Cs in the surface layer of atmospheric air (VA, Bq/m^3^) and the density of radionuclide fallout (DRF, Bq/m^2^) on the ground [7]. In 2024, atmospheric emissions from the BelNPP, in addition to ^137^Cs, included ^3^H, ^14^C, ^60^Co, ^131^I, ^133^I, ^134^Cs, and radioactive noble gases [7]. The radioactive releases from power units No. 1 and No. 2 in the environment were significantly lower than the official standards for permissible radioactive emissions from the BelNPP. Small amounts of ^137^Cs activity were detected in 2024 in the observation zone (12.9 km radius around the NPP) in atmospheric air (the annual average of VA = 0.05 × 10^−5^ Bq/m^3^) and in atmospheric fallout (the DRF range is <0.0007–0.017 Bq/m^2^ × day). The content of ^3^H, ^14^C, and ^90^Sr in atmospheric air was below the VA detection limit of 0.5 Bq/m^3^, 0.5 Bq/m^3^, and 0.09 × 10^−5^ Bq/m^3^, respectively. The level of ^90^Sr fallout was below the detection limit of DRF (0.019 Bq/m^2^ × day) [7].

The gamma- and beta-emitting radionuclide ^137^Cs has a long half-life (T_1/2_ = 30.1 year); it accumulates in the soil and can enter the human body through the food chains [8]. Therefore, to assess the external and internal radiation doses of the population living near nuclear fuel cycle facilities, along with the study of operational indicators (ADER, VA, DRF), it is necessary to monitor the content of technogenic radionuclides in soil, drinking water, agricultural crops and other food products [6, 9, 10]. Practical results of organizing and conducting such environmental monitoring for ^137^Cs near a number of NPPs are provided in various publications [6, 11–17].

When interpreting the results of studying the radiation environment around operating NPPs, the influence of other sources of radioactive contamination of the environment should be considered. For the region where the BelNPP is located, such sources of ^137^Cs are atmospheric fallout due to nuclear weapons tests in the atmosphere and the Chernobyl accident [2]. These events took place long before the NPP began operating. In this case, the integral contribution of the “background” sources to the current level of radioactive contamination can be assessed based on results of a radioecological survey conducted during the pre-startup period of the NPP life cycle [9, 10, 18]. In addition to man-made radionuclides, natural radionuclides (e.g. ^40^K, ^226^Ra, and ^232^Th) are measured in environmental media at the locations of radiation-hazardous facilities. This gives an idea of the extent to which the technogenic contamination affects the integral indicators (dose rate and dose) of the radiation environment.

This study was initiated as part of an independent international (Sweden–Belarus–Russia) research on regular environmental surveys of the radiation situation in the region surrounding the BelNPP [19, 20]. Results of *in situ* measurements of ADER at the 45 land sites located within a 30-km radius zone around the BelNPP have been published recently in Radiation Protection Dosimetry [21]. The goal of this study was to determine the pre-commissioning (2019) levels of soil contamination with ^137^Cs and the resulting gamma dose rate in air at the same 45 sites. For comparison, the dry weight (d.w.) activity concentrations (AC, Bq/kg d.w.) of natural radionuclides (NRN) in the soil and the corresponding values of the gamma dose rate in the air at the same sites were determined.

The aims of this study were:

- to sample 20 cm-deep cores on each of the investigated sites and cut the cores into six layers;
- to measure the AC of ^137^Cs, ^40^K, ^226^Ra, and ^232^Th in the samples at different depths by gamma-ray spectroscopy;
- to calculate the ^137^Cs inventory in soil, i.e. the ^137^Cs soil contamination density (A_Cs_, kBq/m^2^) for the total 20 cm sampling depth of soil and for the top 5 cm deep layer (the sampling depth of 5 cm is often used to determine the density of soil contamination by technogenic radionuclides shortly after single-pulse atmospheric fallout [22]);
- to calculate the absorbed gamma radiation dose rate in air (ADR, nGy/h) and ADER (nSv/h) from ^137^Cs and NRN in soil;
- to compare the ADER values determined in this study based on *ex situ* laboratory measurements of soil samples and the ADER values measured by others [21] *in situ* at the same sites simultaneously with the soil sampling.

## Materials and methods

### Description of the sites

A total of 45 sites of grasslands were selected in September and October 2019 for soil core sampling in the Astrovets district in all directions around the BelNPP reactors (54.76°N, 26.09°E) (Fig.  1). The distance between the NPP and the sampling sites varied from 4 to 29 km. The geographic location of each site (Table 1) was determined using a GPS navigator. The sites codes are given in Table 1, using the same numbering as in Ramzaev *et al.* [21]. The sites were located within 44 populated settlements or near them (at a distance <1 km). The selection of settlements and sampling sites within their area was coordinated with the local administration. Four criteria were considered. First, year-round access to each site by car was considered. Second, sampling points had to be present in all geographic sectors within the observation zone (a 12.9 km radius around the NPP). Third, the list of populated areas for selecting our sites within this zone included all those settlements where observation points for radiation monitoring of atmospheric fallout had been earlier established by the BelNPP itself: Vorona, Vornyany, Goza, Podolcy, Olhovka, Chehi, Markuny, Chernishki, Rymdyuny. Two of our sites are located in the city of Astrovets where there is also a post of the BelNPP radiation monitoring network [7]. Fourth, sampling points were more densely distributed toward the Lithuanian border, given the proximity of a large settlement: Vilnius (the NPP is only ~50 km west). It should be noted that this scattering of sampling points does not fully correspond to the main wind directions in the area: westerly and northwesterly [2].

**Figure 1:**
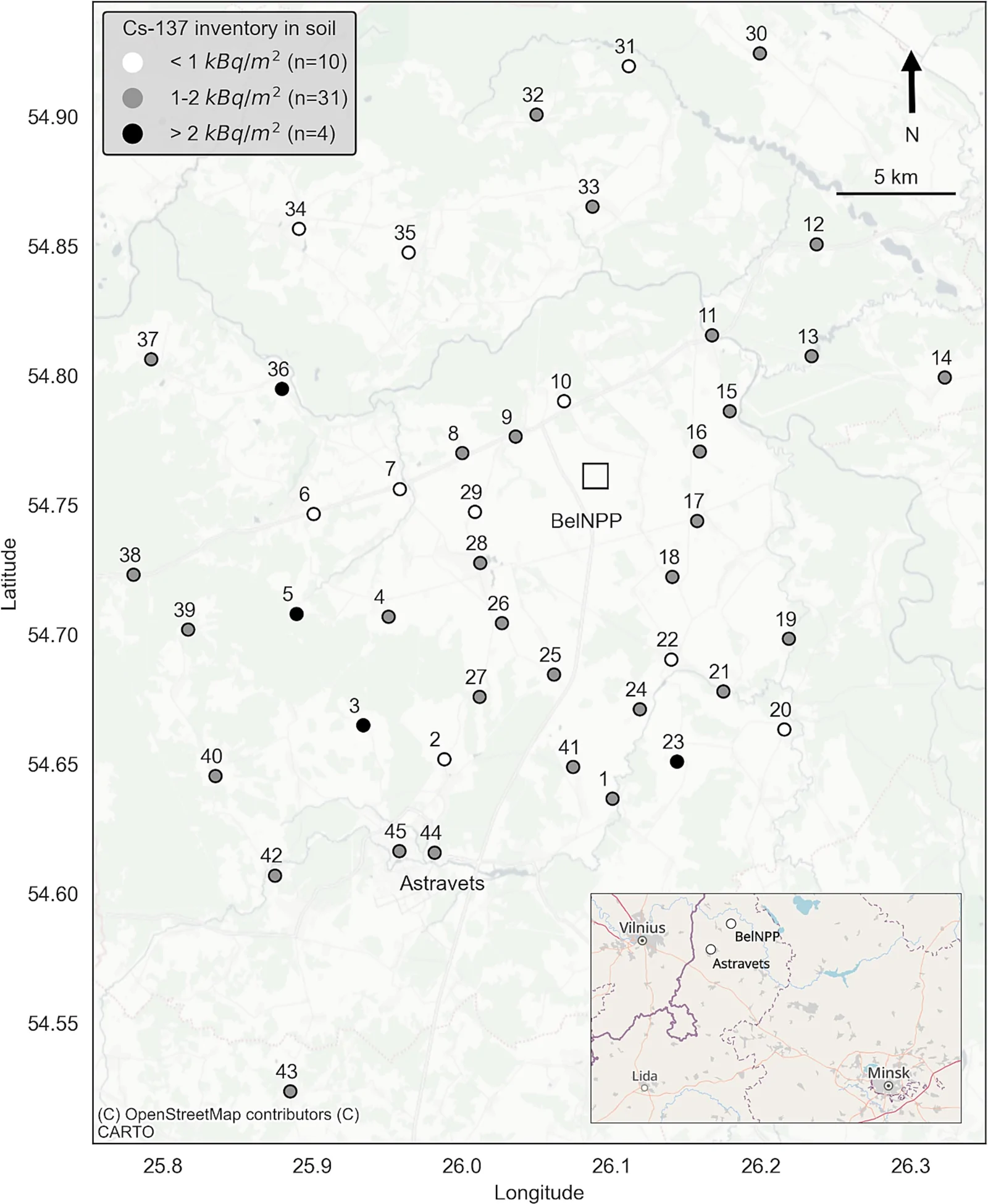
Location of the 45 sites surveyed around the Belarusian nuclear power plant (BelNPP), near the city of Astrovets, in 2019. Inventory of ^137^Cs in the top 20-cm layers in kiloBecquerel per square meter (kBq/m^2^) in three categories with numbers of samples (n) in each category. The site numbers in the map are identical with the numbers in the study by Ramzaev *et al.* [21]. In the smaller map, the location of the city of Vilnius and Minsk are indicated, respectively.

**Table 1:** Name and code of settlements, geographic coordinates, activity concentration (AC_w_) of radionuclides in Becquerel per kilogram in the top 20-cm soil layer (Bq/kg) by dry weight (d.w.) at the sites surveyed around the BelNPP in 2019. Weighted coefficient of variation (%) of the AC_w_ for six sub-samples per site is provided in brackets.

Code of site^a^	Group of sites	Settlement	Geographic coordinates		AC_w_ (Bq/kg, d.w.)			
			Latitude	Longitude	^137^Cs	^226^Ra	^232^Th	^40^K
1^b^	AL	Sosny	54.63655	26.10051	5.07 (24)	7.80 (6.4)	12.4 (8.7)	395 (6.3)
2^b^	AL	Mali	54.65176	25.98813	1.71 (63)	14.3 (8.8)	22.0 (6.2)	519 (2.0)
3^b^	AL	Edoklani	54.66488	25.93396	7.28 (12)	15.6 (4.1)	21.4 (5.4)	536 (2.0)
4^b^	AL	Karveli	54.70678	25.95086	5.84 (13)	22.3 (8.2)	26.4 (3.2)	603 (3.6)
5^c^	AL	Lynkishki	54.70784	25.88936	8.71 (9.4)	19.5 (7.2)	21.6 (4.3)	528 (3.3)
6^b^	AL	Trokeniki	54.74644	25.90078	2.29 (48)	14.6 (3.1)	17.3 (15)	438 (12)
7^b^	OPS	Vorona	54.75603	25.95836	4.00 (18)	24.3 (7.0)	33.0 (5.0)	716 (3.8)
8^b^	AL	Berezovka	54.76996	26.00010	5.97 (56)	21.5 (4.0)	26.2 (3.0)	625 (2.5)
9^b^	AL	Goza	54.77633	26.03576	3.98 (55)	13.5 (4.7)	19.4 (9.4)	496 (2.4)
10^b^	AL	Meshlyany	54.78997	26.06812	2.81 (4.7)	13.6 (7.9)	20.6 (8.0)	524 (2.9)
11^b^	OPS	Mihalishki	54.81542	26.16701	4.69 (72)	10.0 (20)	14.1 (15)	386 (10)
12^b^	OPS	Sidorishki	54.85053	26.23689	7.04 (16)	9.10 (15)	12.6 (11)	406 (5.9)
13^c^	OPS	Olhovka	54.80737	26.23356	4.78 (62)	20.4 (21)	16.3 (10)	376 (3.8)
14^b^	AL	Supronenty	54.79913	26.32256	5.73 (5.2)	14.0 (6.3)	18.9 (5.5)	456 (2.6)
15^b^	AL	Markuny	54.78611	26.17885	5.40 (14)	11.6 (9.2)	17.5 (12)	469 (8.4)
16^b^	AL	Skerdimy	54.77054	26.15885	3.99 (11)	14.0 (4.0)	18.0 (5.8)	529 (3.0)
17^b^	OPS	Chehi	54.74374	26.15709	5.30 (12)	19.2 (8.6)	21.9 (4.7)	556 (2.7)
18^b^	AL	Gaygoli	54.72216	26.14047	6.37 (3.5)	18.8 (6.4)	24.7 (6.8)	606 (5.3)
19^b^	AL	Gelyuny	54.69830	26.21838	6.37 (14)	14.4 (5.5)	20.2 (2.8)	504 (4.4)
20^c^	OPS	Rymdyuny	54.66327	26.21534	1.56 (34)	10.3 (17)	13.6 (6.6)	421 (5.7)
21^c^	OPS	Gudeniki	54.67794	26.17460	6.23 (44)	17.6 (14)	15.5 (9.7)	454 (4.3)
22^b^	OPS	Gervyaty	54.69025	26.13979	1.13 (47)	16.0 (6.9)	24.5 (7.3)	568 (2.9)
23^b^	AL	Galchuny	54.65086	26.14363	7.89 (30)	13.4 (3.8)	17.2 (5.7)	467 (5.4)
24^b^	AL	Matski	54.67106	26.11876	4.82 (17)	15.3 (4.4)	21.5 (12)	520 (2.2)
25^b^	AL	Chernishki	54.68447	26.06141	5.70 (25)	14.6 (2.7)	20.2 (11)	528 (5.6)
26^b^	AL	Kernyany	54.70431	26.02659	5.27 (9.6)	16.9 (4.2)	21.5 (5.4)	528 (2.0)
27^b^	AL	Filipany	54.67585	26.01169	5.16 (13)	13.6 (4.7)	18.1 (6.5)	562 (18)
28^b^	OPS	Vornyany	54.72754	26.01211	4.28 (37)	19.7 (8.1)	25.2 (4.1)	576 (2.4)
29^b^	AL	Bolniki	54.74726	26.00857	3.33 (9.7)	21.3 (4.2)	25.6 (3.9)	605 (2.3)
30^c^	AL	Troschany	54.92423	26.19899	5.65 (8.4)	30.8 (10)	33.0 (4.4)	782 (2.8)
31^c^	OPS	Barani	54.91928	26.11139	2.56 (130)	10.2 (20)	10.9 (8.1)	376 (9.7)
32^c^	AL	Gadiluny	54.90059	26.04973	6.62 (35)	11.8 (17)	13.8 (15)	442 (7.2)
33^b^	OPS	Podolcy	54.86507	26.08713	3.57 (14)	17.1 (3.6)	21.9 (9.4)	565 (2.1)
34^c^	OPS	Kemelishki	54.85651	25.89095	2.02 (18)	17.2 (16)	18.1 (6.6)	537 (5.3)
35^c^	OPS	Rytan	54.84737	25.96423	2.34 (8.7)	17.9 (17)	21.3 (4.3)	599 (2.5)
36^c^	AL	Bystrisa	54.79472	25.87961	6.41 (140)	11.1 (19)	11.8 (9.3)	383 (6.4)
37^c^	OPS	Zharneli	54.80615	25.79220	4.22 (31)	22.1 (7.7)	22.4 (4.5)	621 (1.5)
38^c^	OPS	Osinovka	54.72300	25.78039	5.73 (69)	27.9 (6.3)	31.6 (9.2)	789 (8.5)
39^c^	AL	Zakharishki	54.70183	25.8169	5.24 (25)	14.0 (8.2)	18.4 (7.4)	452 (5.7)
40^c^	AL	Dreveniki	54.64529	25.83516	3.47 (22)	17.8 (20)	22.0 (5.1)	564 (5.1)
41^c^	AL	Radjuli	54.64879	26.07418	6.84 (8.1)	15.9 (11)	19.9 (4.2)	511 (4.2)
42^c^	OPS	Palushi	54.60683	25.87496	4.63 (43)	14.2 (20)	17.3 (2.4)	463 (4.2)
43^c^	OPS	Grodi	54.52358	25.88515	5.71 (19)	25.4 (13)	29.8 (11)	683 (10)
44^c^	OPS	Astrovets-i	54.61570	25.98162	3.12 (53)	16.6 (4.7)	19.3 (4.1)	546 (3.4)
45^b^	OPS	Astrovets-p	54.61631	25.95807	4.36 (53)	16.0 (14)	22.7 (19)	486 (9.6)

^a^The site code numbers correspond to those in Ramzaev *et al.* [21].

^b^Soil samples were analyzed by the RIRH laboratory.

^c^Soil samples were analyzed by the LU laboratory.

AL—agricultural land.

OPS—open public space.

According to the present status of land using, the sites could be roughly classified into two large categories: (i) used and abandoned agricultural lands (26 sites; abbreviation AL in Supplementary materials S1), such as meadows, fields, vegetable gardens, and fruit orchards; (ii) open public spaces (19 sites; abbreviation OPS in Supplementary materials S1), such as parks, yards, squares, playgrounds, and the banks of water reservoirs. The current exterior appearance of the sites was documented using photography (Supplementary materials S2).

### Field work

The soil cores were collected at five points on a site in the “envelope” geometry: at the corners and in the center of a 4.5 m × 4.5 m square with a steel core sampler (length and inner diameter of 25 cm and 5 cm, respectively). The sampler has a detachable, two-layer design: two semicircular, sharpened inner blades and an outer cylindrical shell with blade attachments. The blades are tightly closed vertically, preventing soil particles from entering the shell. This design allows for obtaining a dense vertical soil core that can be subsequently cut into the required number of horizontal layers. The inner surface of the blades is sufficiently smooth, ensuring reliable removal of soil traces on-site with a rag or paper towel after the sampling cycle is complete. Earlier this type of sampler was used for soil sampling in radioactively contaminated areas of Russia [23, 24] and Belarus [25]. The cores were sliced into six horizontal layers corresponding to soil depths of 0–2.5, 2.5–5.0, 5.0–7.5, 7.5–10, 10–15, and 15–20 cm. The five slices from the same depth from the same site were combined. A total of 270 combined samples were obtained from the 45 sites. The samples from 18 sites were measured at Lund University (LU) in Sweden while the samples from other 27 sites were measured at the Research Institute of Radiation Hygiene (RIRH) in Russia.

On the day of soil sampling, ADER was measured at the center of each site using the portable gamma spectrometer-dosemeter MKS AT6101D (ATOMTEX, Belarus) [20]. The detector was placed on a tripod at a height of 1 m above the ground surface. Full technical details of the gamma spectrometer, measurement conditions, and the measurement results are provided in the work by Ramzaev *et al.* [21].

### Laboratory analysis

The soil samples received by the RIRH were air-dried to constant weight at room temperature in the laboratory [25]. The samples at LU were air-dried to constant weight at 70°C. Each dry sample was well mixed using a plastic spatula. Pebbles and plant roots were left in the samples. Therefore, some inhomogeneity in radioactivity distribution within sample material might be anticipated.

Due to logistic problems and the subsequent time gap between field sampling and laboratory stage (for some samples up to 2 years), many samples lost unknown portion of their moisture. Thus, the initial fresh (wet) weight of the samples could not be determined correctly.

Depending on the volume of sample, either the entire sample material (usually from the layers located at the depth of 0–2.5, 2.5–5, 5–7.5, and 7.5–10 cm) or an aliquot (from the layers depth of 10–15 and 15–20 cm) was used. The sample for measurement (counting sample) was placed in a sealed plastic container with a volume of 330 cm^3^ (RIRH) or 200 cm^3^ (LU). The mass of the entire sample and counting sample was determined using electronic scales with the uncertainty <±1 g.

At the RIRH laboratory, the activity measurements were performed using a semiconductor gamma-spectrometer placed inside a chamber with 20 cm thick steel walls. The detector block was additionally surrounded by a 10 cm thick lead shield. The relative efficiency of the HPGe detector was 32%; the energy resolution was 1.8 keV for the 1332 keV line from ^60^Co. The absolute efficiency of the detector was calibrated using certified volumetric activity measures (volume = 330 cm^3^) containing ^137^Cs, ^152^Eu, ^226^Ra, ^232^Th, and ^40^K. The spectrometer was additionally calibrated for measuring the activities in the counting samples of smaller volumes using the Nuclide Master Plus software (Laboratory of Spectrometry and Radiometry, Russia). The activities of ^137^Cs, ^226^Ra, and ^232^Th in the counting samples were determined via the activity of their daughter radionuclides: for ^137^Cs by the 661.6 keV line from ^137m^Ba; for ^226^Ra by the 609.3 keV line from ^214^Bi; for ^232^Th by the 583.1 keV line from ^208^Tl. For ^40^K, its own 1460 keV line was used.

At the LU laboratory, two HPGe detectors and the GammaVision software (ORTEC, USA) were used to conduct the measurements. The resolution for detectors 1 and 2 at 1.33 MeV (^60^Co line) was 1.97 keV and 1.87 keV, respectively. The measured relative efficiency of the HPGe detectors was 92.5% and 118.5%, respectively. The measuring geometry consisted of a 200 ml plastic vessel. The activities of ^137^Cs, ^226^Ra, and ^40^K in the counting samples were determined using the same energy lines as described above. For ^232^Th, the activity was determined by the mean activity of ^228^Ac for the 338 keV and 911 keV energy lines.

A series of intercomparison measurements was performed between the two gamma-ray spectroscopy laboratories [26] with ^137^Cs as the selected reference radionuclide. The AC of the radionuclide was determined in seven soil samples collected in Belarus. The mean difference between the laboratories was 2% (range: −8% to +6%). Additionally, for the quality control of the radionuclides analysis, the reference material IAEA-385 (sediments from Irish Sea) [27] was used. The differences between reference values (IAEA) and experimental values (RIRH and LU) of the ^137^Cs, ^226^Ra, ^232^Th, and ^40^K AC did not exceed ±10% (Table 2).

**Table 2:** Radionuclides AC in Becquerel per kilogram soil by dry weight (Bq/kg, d.w.) in the certified reference sample IAEA-385 [27] measured in laboratories of LU and RIRH.

Laboratory	Radionuclide			
	^137^Cs	^40^K	^226^Ra	^232^Th
*AC (Bq/kg, d.w.)*				
LU	21.2 ± 3.8 (0.58)	602 ± 89 (9)	22.1 ± 4.3 (1.4)	36.3 ± 6.5 (1.8)
RIRH	20.7 ± 2.7 (0.46)	576 ± 81 (6)	20.8 ± 3.3 (1.3)	33.9 ± 5.1 (1.7)
IAEA	19.8 ± 0.6^a^	611 ± 27^a^	22.8 ± 0.6^a^	33.8 ± 0.9^a^
*AC ratio*				
LU/RIRH	1.02	1.05	1.06	1.07
IAEA/LU	1.07	0.98	0.97	1.07
IAEA/RIRH	1.05	0.94	0.91	1.00

Total uncertainty (±) of a measurement is given with a probability of 95%. The minimum detectable AC is provided in brackets. Results corrected to 1 January 2019.

^a^The uncertainty is expressed as an expanded uncertainty using a coverage factor k = 2 [27].

The counting time varied from 15 000 to 320 000 s depending on the activity of the ^137^Cs radionuclide in a counting sample and the laboratory workload.

### Calculation of activity concentrations and dose rates

The measured ^137^Cs activity was recalculated to the sampling date taking into account the half-life of the radionuclide; the corrected AC value was used for all further calculations. No decay correction was applied with respect of the measured ^226^Ra, ^232^Th, and ^40^K activities.

The final results of measurements of ^137^Cs and NRN for each layer are provided in terms of AC (Bq/kg for dry weight). The AC in the entire 0–20 cm layer of soil is provided in terms of average weighted AC (AC_w_, Bq/kg for dry weight) which takes the measured AC and the mass of each primary sub-sample into account (Supplementary materials S1). The weighted coefficient of variation (CV_w_, %) was calculated as an inequality index of radionuclide distribution between the six vertical layers of soil.

Additionally, for ^137^Cs, the activity per unit area or inventory (A_Cs-i_, kBq/m^2^) in the i-th layer was calculated using the formula:


(1) \begin{eqnarray*} {A}_{Cs-i}=A{C}_{Cs-i}\times \frac{m_i}{S}\times 0.001, \end{eqnarray*}

where AC_Cs-i_ is the AC of ^137^Cs in the i-th layer (Bq/kg); m_i_ is the mass of the entire sample from the i-th layer (kg); S is the total area of five cores (0.01 m^2^).

The total ^137^Cs inventory in the upper 20 cm of soil (A_Cs-20_, kBq/m^2^) was calculated by summing the ^137^Cs inventory values in all six layers. The ^137^Cs inventory in the upper 5 cm (A_Cs-5_, kBq/m^2^) was calculated by summing the ^137^Cs inventory values in the top two layers.

The average depth of migration of ^137^Cs in the upper 20 cm of soil (Z, cm) was determined using the formula given in Ramzaev and Barkovsky [23]:


(2) \begin{eqnarray*} Z=\sum \limits_{i=1}^n{Z}_i\times{q}_i, \end{eqnarray*}

where Z_i_ is the depth (cm) of the center of the i-th layer of the soil core, and q_i_ is the fraction of A_Cs-20_ in the i-th layer.

Calculations of the air kerma rate from ^137^Cs in the soil (KR_Cs_, nGy/h) were performed using the equation (2) provided in Ramzaev and Golikov [24]:


(3) \begin{eqnarray*} {\displaystyle \begin{array}{c}K{R}_{Cs}=3.6\times \sum \limits_i{X}_i\times \Big\{\frac{a_1}{a_3}\times \exp \left(-{a}_3\times{z}_{i-1}\right)\\[3pt]\qquad\quad\times \left[1-\exp \left(-{a}_3\times \left({z}_i-{z}_{i-1}\right)\right)\right]\\[3pt] {}+\frac{a_5}{a_6}\times \exp \left(-{a}_6\times{z}_{i-1}\right)\\[3pt]{}\qquad\qquad\times \left[1-\exp \left(-{a}_6\times \left({z}_i-{z}_{i-1}\right)\right)\right]\Big\}\end{array}}, \end{eqnarray*}

where KR_Cs_ is the air kerma rate (nGy/h) at a height of 1 m above the ground; 3.6 is the conversion factor from pGy/s to nGy/h [28]; X_i_ is the AC of ^137^Cs (Bq/g) in the i-th soil layer; z_i_ is the mass depth (in terms of g/cm^2^) of the lower boundary of the i-th layer of soil; a_1_, a_3_, a_5_, a_6_ are empirical coefficients equal, respectively, to: 3.39, 0.492, 2.38, 0.0723 (dimensionless). Before substituting AC values into equation 3, the experimentally obtained AC values in terms of Bq/kg were converted to AC values in terms of Bq/g using a reduction factor of 10^3^.

Equation (3) is a simplification of a formula derived in Golikov *et al.* [28] for calculating KR_Cs_ in a radioactively contaminated forest. It was assumed that the density of aboveground biomass was equal to 0 and the radionuclide was evenly distributed in each layer of soil. Equation (3) was validated by comparing predicted (with soil samples) and measured gamma dose rates in air at the areas in Russia and Belarus heavily contaminated by ^137^Cs after the Chernobyl accident [24, 25, 29]. The relative differences between calculated and measured values of dose rate were in the range from −22% to +31% (probability = 95%) [30]. In environmental gamma-ray measurements, air kerma rate can be considered as an equivalent to absorbed dose rate in air (ADR, nGy/h) [31]. Therefore, below we will use the abbreviation ADR_Cs_ for presenting results of calculation of absorbed dose rate from ^137^Cs.

The absorbed gamma dose rate in air (1 m above the ground surface) from natural radionuclides in soil (ADR_NRN_, nGy/h) was calculated using the values of AC_W_ (Table 1) of ^226^Ra, ^232^Th, and ^40^K in the top 20-cm layer of soil:


(4) \begin{eqnarray*} AD{R}_{NRN}&=\left(A{C}_{w- Ra}\times C{C}_{Ra}\right)+\left(A{C}_{w- Th}\times C{C}_{Th}\right)\nonumber\\&\qquad+\left(A{C}_{w-K}\times C{C}_K\right), \end{eqnarray*}

where AC_w-Ra_, AC_w-Th_, and AC_w-K_ is the AC_W_ of ^226^Ra, ^232^Th, and ^40^K, respectively; CC_Ra_, CC_Th_, and CC_K_ are the conversion coefficients from AC in soil to ADR in air (nGy/h per Bq/kg) for ^226^Ra, ^232^Th, and ^40^K, respectively. The numerical values of 0.462 for CC_Ra_, 0.604 for CC_Th_, and 0.0417 for CC_K_ from the 2000 UNSCEAR report [32] were used in the calculations.

To compare the results of ADR (nGy/h) calculations based on samples measurements *ex situ* with the results of *in situ* measurements of ADER (nSv/h) at the same sites [21], a conversion coefficient of 1.3 Sv/Gy (for ^137^Cs) and 1.25 Sv/Gy (for NRN) provided in the study by Ramzaev and Barkovsky [33] was used. The value of 1.3 Sv/Gy for ^137^Cs had been experimentally obtained via direct measurements at cultivated lands (kitchengardens) heavily contaminated by Chernobyl fallout. This value corresponds well to the value of 1.29 Sv/Gy that can be calculated based on values of KR and ADER recently deduced by Satoh and Petoussi-Henss [34] with a computer simulation for ^137^Cs uniformly distributed in soil. Note, that for relatively fresh fallout and exponential vertical distribution of ^137^Cs in soil, somewhat smaller conversion coefficient (1.25–1.26 Sv/Gy) is applicable [31]. The value of 1.25 Sv/Gy adopted in our study for NRN agrees well with ratio of 1.25, 1.24, and 1.21 Sv/Gy that can be computed based on KR and ADER data for the radionuclides of the ^238^U series, ^232^Th series, and ^40^K uniformly distributed in soil [34].

The values of ADER calculated from ^137^Cs (ADER_Cs_, nSv/h) and NRN (ADER_NRN_, nSv/h) in soil were summed and indicated as ADER_Cs + NRN_. To obtain ADER_Cs + NRN_ values for the set of *in situ* measurements, the value of the intrinsic noise of the detector and its response to cosmic radiation (8 nSv/h) [21] was subtracted from the dosemeter total reading values (ADER_tot_) that were provided in Table 1 in Ramzaev *et al.* [21].

### Statistical analysis

Parametric and non-parametric statistical methods were applied. The Shapiro–Wilk test was used to determine whether a sample came from a normally distributed population (*P*-value more than .05). The difference between groups was tested using the Mann–Whitney U test (independent samples). Spearman’s rank correlation coefficient was calculated to determine the degree of correlation between the group datasets. Statistically significant differences and correlations between groups were defined as comparisons resulting in *P* < .05. Statistical treatment of the data was performed using EXCEL for Windows and on-line free access platforms (https://www.socscistatistics.com and https://www.gigacalculator.com).

## Results and discussion

### ^137^Cs in soil

The 661.6 keV peak net area was quantified in 268 of the 270 samples. The AC values in the 268 samples varied from 0.43 to 20.8 Bq/kg (see Supplementary materials S1). The one sigma uncertainty in the 661.6 peak area measurement was <10% for the great majority (*n* = 217) of samples (mean = 7.5%). For two samples, the 661.6 keV peak was not formed. The ^137^Cs AC in the samples were below the calculated detection limit (DL) of 0.46 Bq/kg. For these samples, the value of 0.5 DL (0.23 Bq/kg) was used for deriving AC_w_ and A_Cs-20_.

The scatter of the AC through the soil column varies significantly between sites (Supplementary materials S1) as demonstrated by the wide range of CV_w_ of the AC values (4%–140%, mean = 32%, see Table 1). Examples of such uneven activity distributions along the vertical soil profile are shown in Fig.  2. Depending on the site, the maximum AC value could be observed in the upper soil layers or, on the contrary, was measured in the deepest layers. However, in many cases, the distribution of the AC was relatively uniform through the soil column. Such sites presenting a uniform distribution were predominantly located within agricultural lands (e.g. the site 18 in Fig.  2). The homogenization of the top soil layer can be explained by agricultural practices like plowing. However, it should be noted that the type of vertical distribution could not be unambiguously linked to the category of land use (see Fig.  2). The surface soil layer of sites in the open public space category is also subject to human influence. These sites are regularly visited by people and are sometimes used for the passage of cars and other vehicles. For ease of use, in many cases (e.g. site 20, 22, 44), such sites are pre-leveled. The surface layer can be mechanically moved and/or filled with imported soil. The soil in parks is occasionally cultivated.

**Figure 2:**
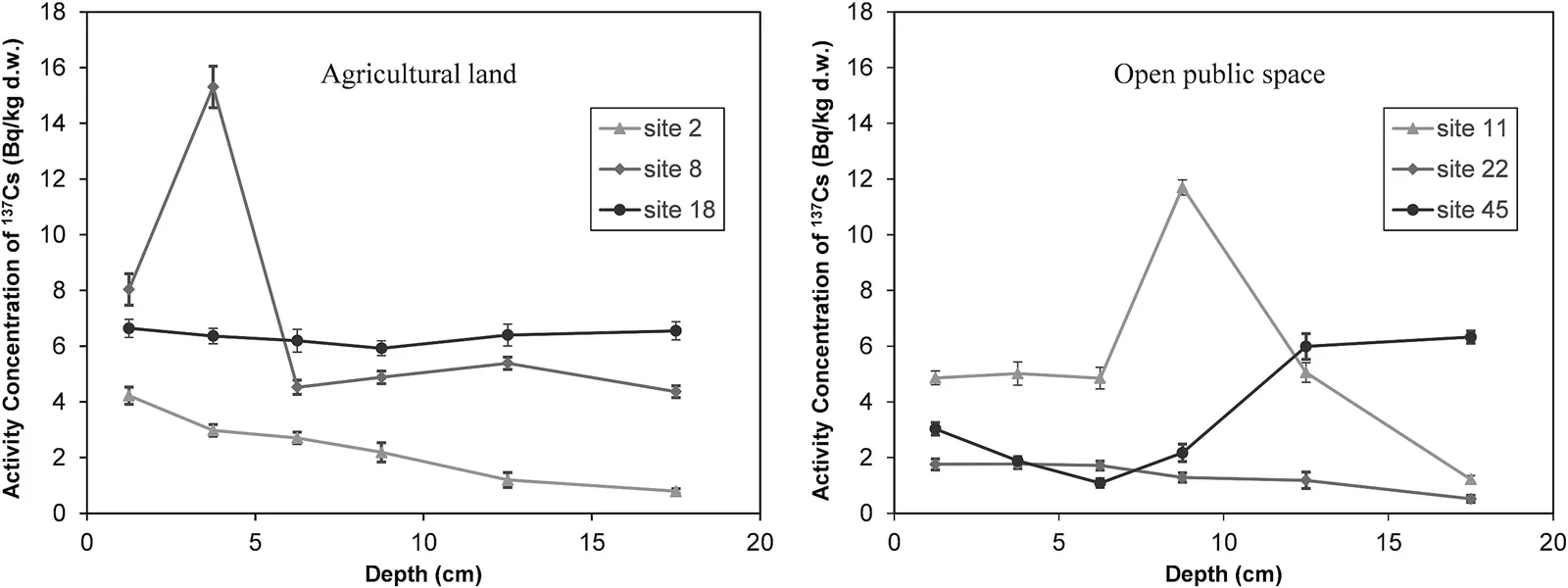
Examples of the vertical distribution of ^137^Cs AC in soil at 0 to 20 centimeter (cm) depths in Becquerel per kilogram dry weight (Bq/kg, d.w.) at the three sites from the agricultural land and open public space, respectively. Uncertainty bars at 1 sigma level are shown for each depth. Samples are surveyed around the Belarusian nuclear power plant in 2019.

Very pronounced differences were also observed between the surveyed sites in the AC_w_ of ^137^Cs in the upper 20 cm of soil (Table 1). The AC_w_ values varied by seven-fold and were in the range of 1.13–8.71 Bq/kg (mean = 4.78 Bq/kg, median = 5.07 Bq/kg). It is worth noting that the AC_w_ of ^137^Cs is normally distributed between the 45 sites surveyed in this work (Shapiro–Wilke test, *P*-value = .60) as shown in Fig.  3. The differences between the sites in terms of the ^137^Cs inventory in the upper 20 cm soil layer (A_Cs-20_) were also large: from 0.36 to 2.54 kBq/m^2^ (Table 3). The mean A_Cs-20_ value for the whole set of the 45 sites was 1.35 kBq/m^2^ (median = 1.39 kBq/m^2^). The top 5-cm soil layer contained, on average, 24% (range 7%–54%) of the total radionuclide inventory in the 20-cm layer. The absolute A_Cs-5_ values ranged from 0.05 to 1.11 kBq/m^2^ (Table 3) with the mean value of 0.32 kBq/m^2^. The average depth of ^137^Cs migration in the top 20-cm layer of soil (Z, Table 3) varied from 5.1 to 14.2 cm (average = 10.4 cm). The pronounced differences between the sites in terms of Z may be associated with differences in the natural vertical and horizontal migration of the radionuclide in the soil and with human activity at the sites after the radionuclide deposition on the ground surface. Ramzaev *et al.* [25] reported that the center of ^137^Cs vertical migration (Z parameter) in 2016–18 was located three times deeper in cultivated meadows and arable lands compared to virgin meadows at heavily contaminated areas in the Gomel region of Belarus.

**Figure 3:**
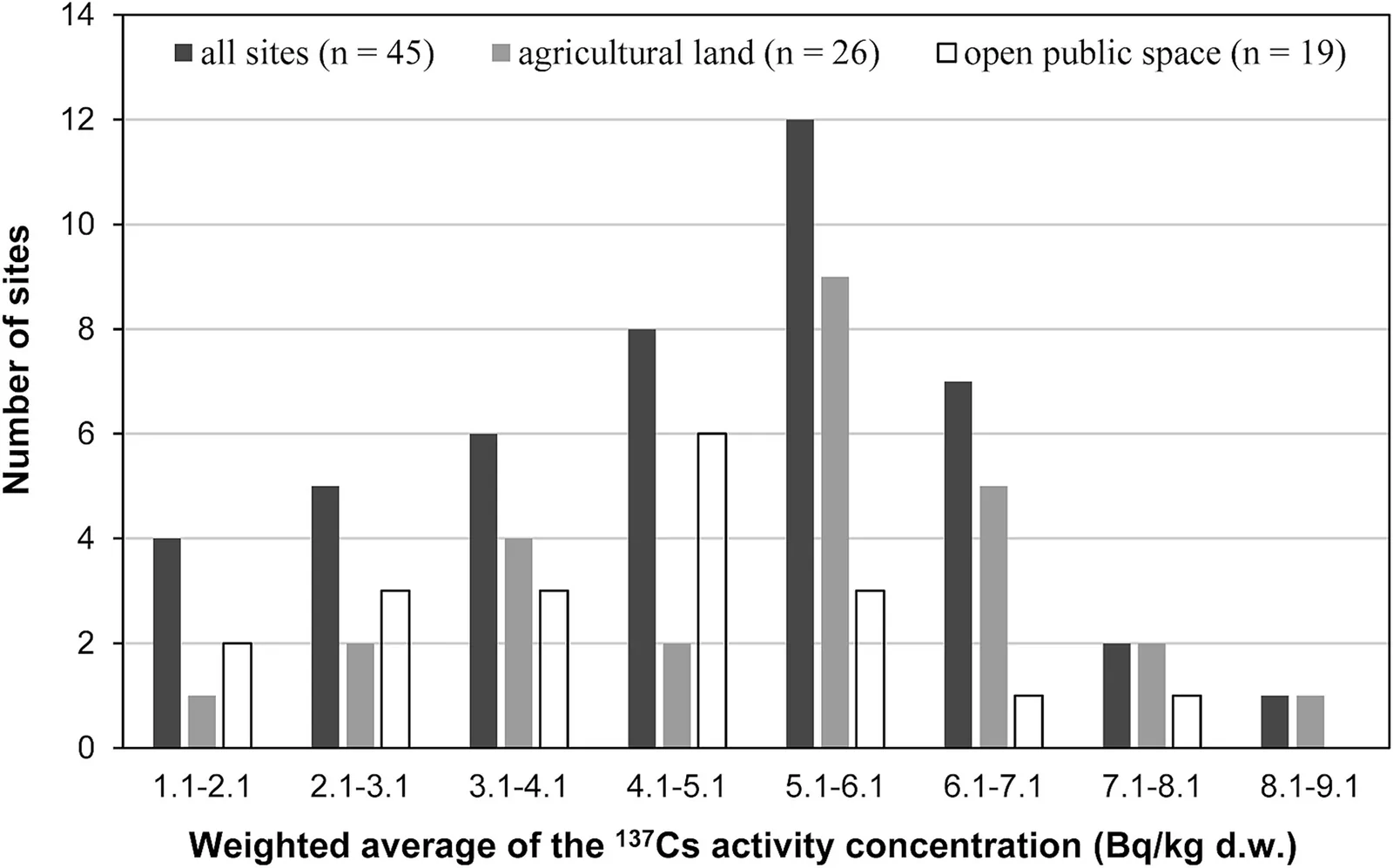
Distribution of weighted average AC of six soil fractions of ^137^Cs in Becquerel per kilogram dry weight (Bq/kg, d.w.) for the 45 sampled sites divided in all sites, agricultural land and open public space, respectively.

**Table 3:** Inventory of ^137^Cs activity concentration (A_Cs_) in the top 5-cm and 20-cm soil layers in kiloBecquerel per square meter (kBq/m^2^), mean migration depth in centimeter (cm) of ^137^Cs in the top 20-cm soil layer (Z), calculated absorbed dose rate (ADR) from ^137^Cs (Cs) and natural radionuclides (NRN) in nanoGray per hour (nGy/h) in the soil and total ambient dose equivalent rate (ADER) from ^137^Cs and NRN (ADER_Cs + NRN_) in nSv/h based on *ex situ* soil analysis and *in situ* measurements at the 45 sites around the Belarussian nuclear power plant.

Code of site^a^	A_Cs_ (kBq/m^2^)		Z (cm)	ADR (nGy/h)			ADER_Cs + NRN_ (nSv/h)		Ratio *in situ/ex situ*
	0–5 cm	0–20 cm		Cs	NRN	Cs + NRN	*ex situ*	*in situ* ^c^	
1	0.28	1.32	11.1	0.73	27.5	28.2	35.3	32.6	0.92
2	0.15	0.51	8.9	0.33	41.6	41.9	52.4	48.4	0.92
3	0.35	2.13	11.4	0.96	42.5	43.4	54.3	50.0	0.92
4^b^	0.25	1.34	10.7	0.72	51.4	52.1	65.2	61.6	0.94
5	0.53	2.54	10.9	1.18	44.1	45.2	56.6	44.1	0.78
6	0.05	0.68	13.9	0.20	35.5	35.7	44.6	36.4	0.82
7	0.16	0.96	11.7	0.55	61.0	61.5	76.9	63.6	0.83
8	0.49	1.55	10.4	0.94	51.8	52.8	66.0	57.3	0.87
9	0.38	1.11	8.1	0.65	38.7	39.3	49.2	41.0	0.83
10	0.16	0.85	11.5	0.36	40.6	41.0	51.2	46.0	0.90
11	0.26	1.41	9.6	0.69	29.2	29.9	37.4	33.7	0.90
12^b^	0.38	1.99	11.0	0.98	28.7	29.7	37.2	31.6	0.85
13^b^	0.44	1.32	8.4	0.87	35.0	35.8	44.8	37.7	0.84
14^b^	0.38	1.65	11.1	0.76	36.9	37.7	47.2	46.3	0.98
15	0.32	1.57	10.5	0.67	35.5	36.1	45.2	40.9	0.90
16	0.20	1.17	11.7	0.48	39.4	39.9	49.9	42.7	0.86
17	0.27	1.39	10.9	0.67	45.3	46.0	57.5	55.8	0.97
18	0.33	1.72	11.5	0.81	48.9	49.7	62.2	53.8	0.86
19	0.31	1.60	11.0	0.84	39.9	40.7	50.9	43.5	0.85
20	0.07	0.50	13.1	0.18	30.5	30.7	38.4	35.0	0.91
21	0.38	1.37	8.7	0.82	34.3	35.1	46.6	35.7	0.77
22	0.10	0.36	9.6	0.19	45.9	46.1	57.6	47.4	0.82
23	0.50	2.13	10.0	1.12	36.1	37.2	46.6	40.6	0.87
24	0.33	1.35	10.3	0.66	41.7	42.4	53.0	45.2	0.85
25	0.40	1.55	10.0	0.81	41.0	41.8	52.3	45.0	0.86
26^b^	0.37	1.37	10.4	0.68	42.8	43.5	54.4	54.6	1.00
27^b^	0.33	1.43	10.8	0.70	40.7	41.4	51.7	51.1	0.99
28	0.33	1.24	9.4	0.65	48.4	49.0	61.3	55.9	0.91
29	0.22	0.95	10.7	0.45	50.5	51.0	63.7	54.5	0.86
30	0.33	1.61	11.4	0.73	66.8	67.5	84.4	57.6	0.68
31	0.45	0.83	5.2	0.61	27.0	27.6	34.9	24.1	0.70
32	0.32	1.78	10.7	0.91	32.2	33.1	41.5	32.3	0.78
33	0.26	1.04	10.6	0.46	44.7	45.1	56.4	45.5	0.81
34	0.12	0.53	11.2	0.28	41.3	41.6	52.0	38.5	0.74
35	0.17	0.68	10.5	0.31	46.1	46.4	58.0	43.3	0.75
36	1.11	2.04	5.1	1.55	28.2	29.8	37.3	32.4	0.87
37	0.29	1.28	10.0	0.62	49.7	50.3	62.9	49.4	0.79
38	0.44	1.54	8.2	0.97	64.9	65.9	82.4	54.1	0.66
39	0.40	1.62	10.4	0.74	36.4	37.2	46.5	45.5	0.98
40	0.18	1.11	12.0	0.39	45.0	45.4	56.8	47.1	0.83
41	0.38	1.73	10.9	0.83	40.7	41.5	51.9	41.6	0.80
42	0.54	1.51	8.9	0.82	36.3	37.1	46.4	39.0	0.84
43	0.30	1.54	11.6	0.68	58.5	59.2	74.1	53.7	0.73
44	0.32	1.14	8.9	0.55	42.1	42.7	53.3	53.6	1.01
45	0.14	1.45	14.2	0.41	41.4	41.8	52.2	50.1	0.96

^a^The site code numbers correspond to those in Ramzaev *et al.* [21].

^b^The site was surveyed during a rainfall or soon after a rainfall.

^c^The data are taken from Ramzaev *et al.* [21].

Given the different land uses in the groups of agricultural lands and open public spaces, it is entirely reasonable to conduct an intergroup comparison. The Shapiro–Wilk test and Fig.  3 indicate that for both groups we cannot reject the hypothesis that the ^137^Cs AC_w_ and A_Cs-20_ data have come from a population which had a normal distribution (Table 4). The ^137^Cs AC_w_ and A_Cs-20_ in the group of agricultural lands, compared to the group of open public spaces, are higher by ~30% (Table 4). The differences are statistically significant (*P* < .05). These differences may indicate the artificial removal of some amount of ^137^Cs-containing soil from a part of the sites in the open public space group during their adjustment to the human environment. However, based on calculation of the average depth of migration of ^137^Cs in the upper 20 cm of soil (Table 4, Z), the group of agricultural lands does not differ statistically significant from the group of open public spaces (*P* > .05). It can be assumed that the effects of human impact on the vertical distribution of ^137^Cs through the surface soil layer in the group of agricultural lands were, on average, comparable to those in the open public space group.

**Table 4:** The average weighted activity concentration in soil of ^137^Cs (AC_w_) in Becquerel per kilogram dry weight (Bq/kg, d.w.), total inventory of ^137^Cs in the top 20-cm of soil (A_Cs-20_) in kiloBecquerel per square meter (Bq/m^2^), and the mean migration depth (Z) in centimeter (cm) by agricultural land (AL) and open public space (OPS), respectively.

Parameter	AC_w_ (Bq/kg, d.w.)		A_Cs-20_ (kBq/m^2^)		Z (cm)	
	AL	OPS	AL	OPS	AL	OPS
Minimum	1.71	1.13	0.51	0.36	5.1	5.2
Maximum	8.71	7.04	2.54	1.99	13.9	14.2
Median	5.53	4.28	1.55	1.28	10.8	10.0
Mean	5.30	4.07	1.48	1.16	10.6	10.1
S.d.	1.68	1.62	0.46	0.43	1.5	2.0
n	26	19	26	19	26	19
*P*-value	<.05		<.05		>.05	

All values were found to be normally distributed (the Shapiro–Wilk test, *P* > .05) except for the mean migration depth (Z) at the group of agricultural land. Results of the intergroup comparison (the Mann–Whitney U test) are provided in the last row.

S.d.—standard deviation.

The average level of ^137^Cs soil contamination determined in this study around the BelNPP is consistent with the A_Cs_ estimates (<2 kBq/m^2^) given by De Court *et al.* [35] for this European region which was not significantly contaminated by Chernobyl fallout. Moreover, our A_Cs_ estimates are in agreement with the results of the *in situ* gamma-ray spectroscopy measurements performed by Ramzaev *et al.* [21] at the same sites on the days of soil sampling: in all cases, the A_Cs_ value was below the detection limit of 4 kBq/m^2^ stated for the *in situ* measurements. In general, the background A_Cs_ values in the region of the BelNPP location are 2–3 orders of magnitude lower than the current ^137^Cs soil contamination density in different areas of Belarus affected by the Chernobyl accident. In particular, in settlements of the Gomel region in 2016–18, the values of ^137^Cs inventory in soil reached 498 kBq/m^2^ (the Khoiniki district [36]) and 1620 kBq/m^2^ (the Vetka district [25]).

Our data on pre-operational ^137^Cs soil contamination levels at specific sites can serve as a baseline data primarily for assessing regional fallout levels in the event of an accidental ^137^Cs release from the plant itself or other NPPs in Europe. Soil samples are suitable for establishing baseline condition but are not suitable for routine monitoring of atmospheric radioactive fallout rates, since, according to routine plate studies, the ^137^Cs fallout density in the Belarusian NPP observation zone is very low: in 2024, this indicator varied within the range of <0.0007–0.017 Bq/m^2^ [7]. However, during normal NPP operation, regular (e.g. once every 5 years) soil sampling at known sites and the subsequent radiometric analysis can be useful for risk communication regarding the dynamics of the radiation environment around the NPP. In particular, this can be used to assess the radiation situation in those settlements where permanent monitoring posts of the BelNPP are located. In this important, socially significant area of using independent scientific data, it will be appropriate to compare information on the current level of soil contamination around the NPP with the corresponding background (pre-operational) data and the information on the current levels of radioactive contamination of other regions of Belarus.

### Natural radionuclides in soil

The activity of NRN was quantified in all samples (*n* = 270). The AC values of ^226^Ra, ^232^Th, and ^40^K in the samples varied in the range of 5.6–33.9, 9.3–35.5, and 313–872 Bq/kg, respectively (Supplementary materials S1). The statistical uncertainty (1 sigma) on the count for the photopeaks defined in the Materials and methods section to determine the activity of ^226^Ra, ^232^Th, and ^40^K were on average 8.3%, 5.9%, and 2.2%, respectively. Difference in the average AC of NRN were observed from site to site, however the variability of AC between layers within a site was relatively small compared to ^137^Cs: the coefficient of variation of the AC (Table 1) averaged 9.7% (range 2.7%–21%), 7.5% (range 2.4%–19%), and 5.0% (range 1.5%–18%) for ^226^Ra, ^232^Th, and ^40^K, respectively. The AC_w_ of NRN in the top 20 cm of soil (Table 1) varied from 7.8 to 30.8 Bq/kg (mean = 16.5 Bq/kg, median = 15.9 Bq/kg) for ^226^Ra, from 10.9 to 33.0 Bq/kg (mean = 20.5 Bq/kg, median = 20.2 Bq/kg) for ^232^Th, and from 376 to 789 Bq/kg (mean = 527 Bq/kg, median = 528 Bq/kg) for ^40^K. The Shapiro–Wilk test indicated normal distribution of data for the AC_w_ of ^226^Ra (*P* = .094) and ^232^Th (*P* = .087), whereas the distribution was not normal (*P* = .029) for the ^40^K AC_w_. The distribution of data for the AC_w_ of NRN in the agricultural lands and open public space groups can be considered as normal (The Shapiro–Wilk test, *P* > .05), excluding the AC_w_ of ^226^Ra in the agricultural lands group (Table 5). No statistically significant differences were observed between the agricultural lands and open public space groups with respect of the ^226^Ra, ^232^Th, and ^40^K AC_w_ (the Mann–Whitney U test, *P* > .05).

**Table 5:** The average weighted activity concentration AC in soil of natural radionuclides (AC_w_) in Becquerel per kilogram dry weight (Bq/kg, d.w.) by agricultural land (AL) and open public space (OPS), respectively.

Parameter	AC_w_ (Bq/kg, d.w.)					
	^40^K		^226^Ra		^232^Th	
	AL	OPS	AL	OPS	AL	OPS
Minimum	383	376	7.8	9.1	11.8	10.9
Maximum	782	789	30.8	27.9	33.0	33.0
Median	522	546	14.5	17.2	20.2	21.3
Mean	522	533	15.8	17.4	20.4	20.6
S.d.	82	118	4.5	5.3	4.5	6.3
n	26	19	26	19	26	19
*P*-value	>.05		>.05		>.05	

All values were found to be normally distributed (the Shapiro–Wilk test, *P* > .05) except for the AC_w_ of ^226^Ra at the group of agricultural land. Results of the intergroup comparison (the Mann–Whitney U test) are provided in the last row.

S.d.—standard deviation.

Over the 45 sites surrounding the BelNPP, the median values of the ^226^Ra and ^232^Th AC in soil were much lower compared to the worldwide median AC values of 35 Bq/kg and 30 Bq/kg, respectively, while the median AC of ^40^K was, on the contrary, higher than the worldwide median AC of 400 Bq/kg [32].

### Gamma dose rate in air

Calculated values of ADR_Cs_ (Table 3) varied from 0.18 to 1.58 nGy/h (mean = 0.68 nGy/h, median = 0.68 nGy/h). The values of ADR_NRN_ (Table 3) were in the range 27.0–66.8 nGy/h (mean = 42.0 nGy/h, median = 41.3 nGy/h). The contribution of ^137^Cs to the total ADR (from NRN and ^137^Cs) was on average 1.7% (range = 0.4%–5.2%). The ADER_Cs + NRN_ (Table 3) was in the range 34.5–84.4 nSv/h (mean = 53.3 nSv/h, median = 52.2 nSv/h). The values of ADER_Cs + NRN_ obtained with measurements of soil samples *ex situ* correlate positively with the values of ADER_Cs + NRN_ that had been directly measured *in situ* by Ramzaev *et al.* [21] at the sites during soil sampling (Spearman’s correlation coefficient is 0.885, *P* < .01; *n* = 45). The ratio of measured values to calculated values of ADER_Cs + NRN_ (Table 3) varied from 0.66 to 1.01 (mean = 0.86, median = 0.86). Graphically, the relationship is depicted in Fig.  4. Lower average and median values were obtained from the directly measured dose rates compared to the calculated ones since the calculation were performed based on the dry weight of soil. Indeed, the soil moisture is a shielding factor for the radiation from radionuclides in soil [37, 38]. The UNSCEAR [32] uses the conversion factor (dry to wet basis) of 0.81 as representative value of soil moisture of 30% by volume and a soil density of 1.6 g/cm^3^ (wet weight). Therefore, the mean and median value of 0.86 for the *ex situ/in situ* ratio determined in this work is in reasonable agreement with the factor of 0.81 used by UNSCEAR to convert *ex situ* values of NRN to *in situ* values.

**Figure 4:**
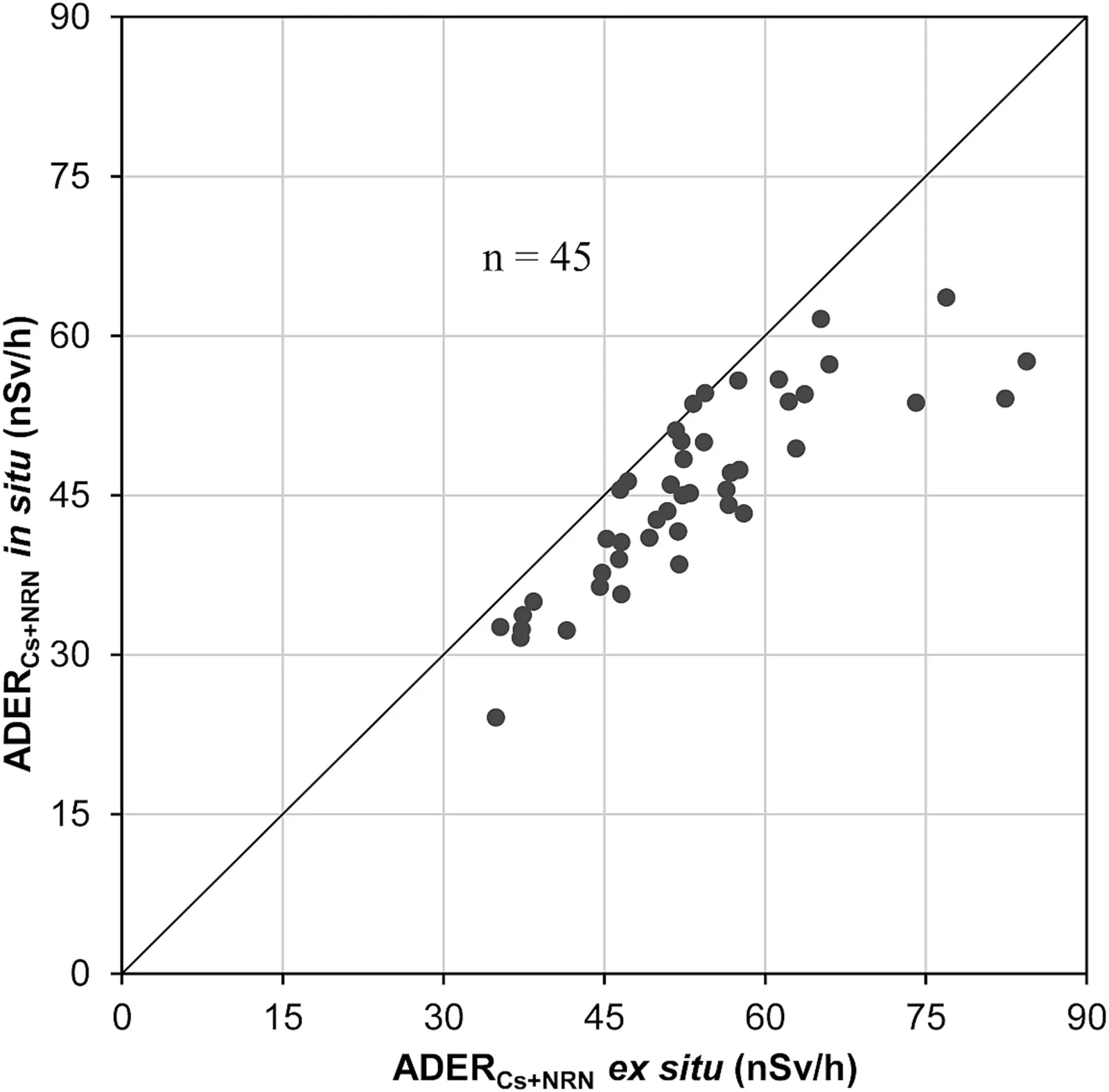
Relationship between the total ADER from ^137^Cs and natural radionuclides (ADER_Cs + NRN_) in nanoSievert per hour (nSv/h) correlating *ex situ* soil analysis with *in situ* measurements by Ramzaev *et al.* [21] at the 45 sites around the Belarusian nuclear power plant. The solid line indicates 1:1 ratio.

### Limitations of the study

There are several limitations in the study. Measurements of the radionuclide activity in soil samples from different sites were performed in two independent laboratories; each laboratory had its own standards for determining activity in the samples. Therefore, the observed variation in radionuclides AC between sites may be due, to some extent, to interlaboratory differences. To determine the significance of this factor, we conducted interlaboratory comparisons using the certified reference material IAEA-385. The differences between the laboratories were within 7%. The difference was particularly small for the main radionuclide of interest, ^137^Cs (only ~2%).

Radioactivity measurements were performed for soil samples collected only to a depth of 20 cm. For a number of sites, the vertical distribution pattern of ^137^Cs indicated the possibility of some portion of the total radionuclide inventory occurring at a depth exceeding 20 cm (e.g. site 18 and site 45 in Fig.  2).

We used data only for the 20 cm cores to calculate KR_Cs_. Thus, an underestimation of the true KR_Cs_ value might be expected. However, taking into account the attenuation of radiation intensity due to the overlying 20 cm soil layer, the contribution of the deep-lying source to the total air KR_Cs_ will be insignificant. To address this issue, authors of the work [24] sampled 30 soil plots in the Chernobyl contamination zone in 2010. It was deduced that, depending on the type of vertical distribution, the contribution from the layer with a depth of 20–30 cm could vary from 0.004% to 8.6%.

The water content was not determined at the sampling sites. However, it was assumed that the content of soil moisture may vary from site to site and that it can be a factor influencing the scatter (Fig.  4) of the *ex situ/in situ* ratio determined for each site (Table 3). Besides the effect of humidity, the range of the ratio of *in situ* to *ex situ* values can be associated, to some extent, with the statistical uncertainties of ADER_Cs + NRN_ measurements *in situ* and uncertainties of the ADER_Cs + NRN_ derivation based on soil samples analysis *ex situ*.

Another limiting factor is the fact that in six cases (marked with the symbol^b^ in Table 3) the *in situ* measurements were made during rainfall or shortly after rainfall. It is known that the gamma radiation dose rate in the air can increase sharply during rainfall [39]. This is due to the washing out of radon decay products from the atmosphere and their deposition on the ground [39, 40]. For the six cases we sampled during rainfall, the average ratio of *in situ* to *ex situ* values of ADER_Cs + NRN_ was 0.93 (median = 0.96). The values of both parameters exceeded averages for the whole sample.

Two of the surveyed sites were closer to large buildings. The site Astrovets-i (No. 44) was surrounded by one-storey and multi-storey buildings. The site Chehi (No. 17) was near a large multi-storey stone building. For the two sites, the *in situ* to *ex situ* ratio values (1.01 and 0.97) were among the highest values in the whole set. The relatively high value of the *in situ* to *ex situ* ratio can be associated with contribution of natural gamma-radiation sources in the building materials (brick, concrete). Medeiros and Yoshimura [41] reported that in the city of San Paulo (Brazil) in some outdoor sites (squares) the buildings enhanced the background gamma-radiation levels estimated only from soil for the semi-infinite geometry at 1 m above the ground surface by ~35%.

After excluding the eight sites discussed above (the six rainfall and the two large buildings), the correlation between the ADER_Cs + NRN_ values obtained *in situ* and *ex situ* become stronger: Spearman’s correlation coefficient is 0.920, *P* < .01; *n* = 37. The mean and median value of the *in situ* to *ex situ* ratio for the group of the 37 sites located far away from large buildings and surveyed in dry weather conditions is 0.84 and 0.85, respectively; these are ~10% lower than those for the group of six plots surveyed in rainy weather. The difference between the two latter groups is statistically significant (the Mann–Whitney U test, *P* <.05).

## Conclusions

The study was performed within the framework of an independent research project for environmental surveys of the radiation situation in the region surrounding the BelNPP. Measurements of soil radioactivity were conducted in two laboratories located in Sweden and Russia.

The study revealed low levels of soil contamination by ^137^Cs in 45 sites around the BelNPP in the pre-commissioning period. The inventory of ^137^Cs in the upper 20-cm soil layer varied from 0.36 to 2.54 kBq/m^2^. Over the 45 sites surrounding the BelNPP, the median values of the ^226^Ra and ^232^Th AC in soil were much lower compared to the worldwide median AC values, while the median AC of ^40^K was, on the contrary, higher than the worldwide median AC. The total ADER from ^137^Cs and NRN (ADER_Cs + NRN_) deduced from soil samples was in the range 34.5–84.4 nSv/h. The contribution of ^137^Cs to ADER_Cs + NRN_ was on average 1.7% (range = 0.4%–5.4%). A good agreement between the ADER_Cs + NRN_ values calculated with the soil samples and the ADER_Cs + NRN_ values measured *in situ* at the same sites previously is observed (Spearman’s correlation coefficient is 0.885, *P* < .01; *n* = 45). The comparison had two main limitations. The ADER_Cs + NRN_ calculation was performed using dry soil samples. The ADER measurements *in situ* were conducted at six sites during rainfall and at two sites near large stone buildings.

The study showed that when studying the ^137^Cs content in soil, the land use category should be considered: the ^137^Cs AC_w_ and A_Cs-20_ in the agricultural lands group, compared to the open public space group, were higher by ~30%. The differences were statistically significant (*P* < .05). This may be associated with the removal of some ^137^Cs-containing soil from some of the sites in the open public space group during their adjustment to the human environment. However, land use does not appear to have a significant impact on the content of natural radionuclides in soil: no statistically significant differences were observed between the agricultural lands and open public space groups for the ^226^Ra, ^232^Th, and ^40^K AC_w_ (*P* > .05). The site-to-site variations in the pre-operational ^137^Cs soil contamination and the ranges of ADER_Cs + NRN_ should be taken into account when assessing the radiation environment in the vicinity of the BelNPP. Indeed, future environmental radiological programs would benefit from re-surveying the plots characterized in this work with established pre-operational levels of ^137^Cs, ^226^Ra, ^232^Th, and ^40^K in soil as well as of gamma-dose rates in air.

## Supplementary Material

Supplementary-S2-to-Baseline_levels_of_137Cs-Bel_NPP-26-05-2025_ncag063
