# Supplementary material for: Activity concentration of 137Cs and natural radionuclides in soil around the Belarusian nuclear power plant in the pre-commissioning period
Source: Radiat Prot Dosimetry. 2026 Jun 29;202(16):1291–305. doi: 10.1093/rpd/ncag063 (PMC13639805; doi:10.1093/rpd/ncag063)

Plots surveyed by the joint expedition of Lund University, Research Institute of Radiation Hygiene and Institute of Radiobiology around BelNPP in 2019.

Sosny-Plot-1. 16-09-2019.

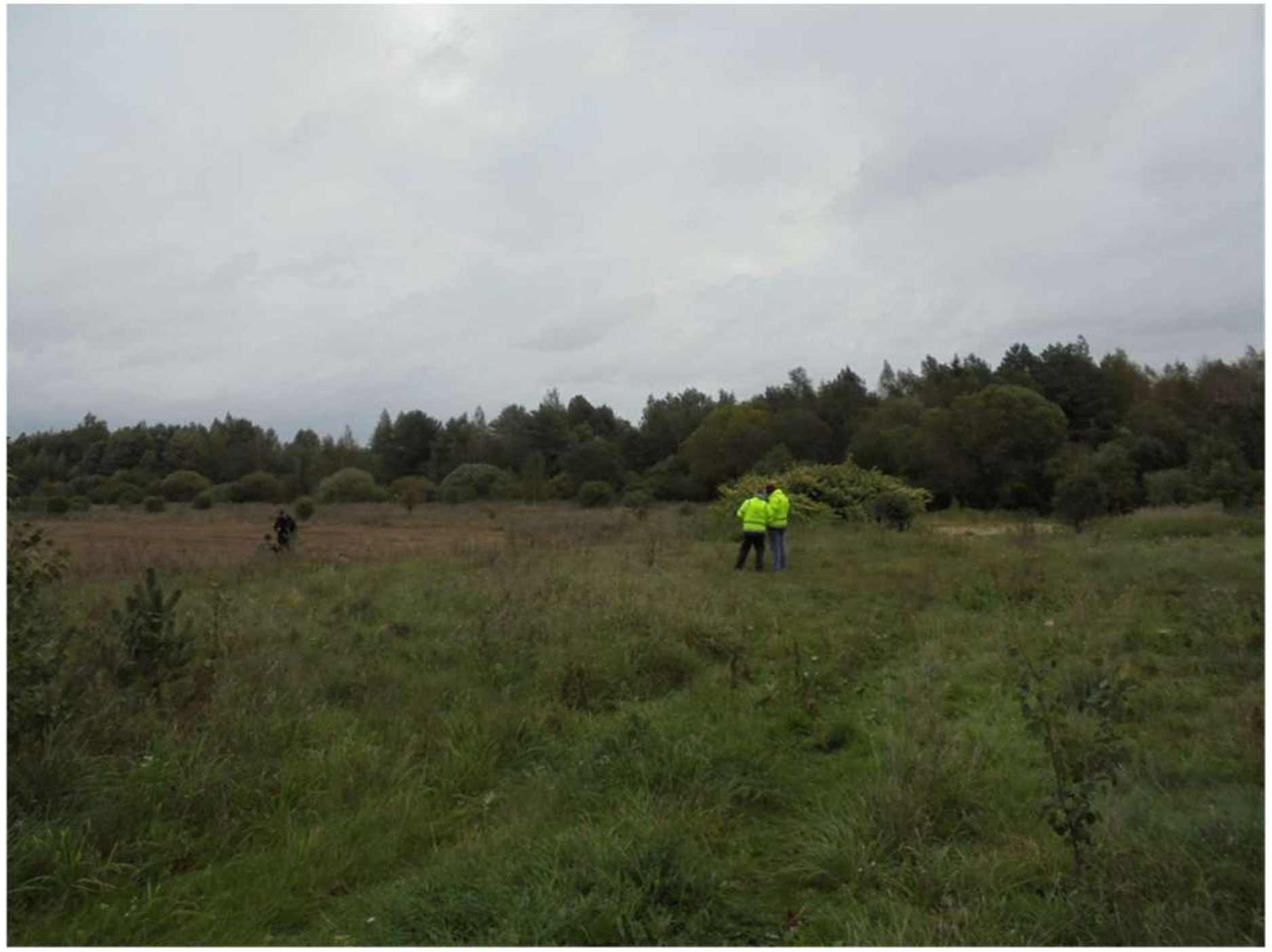

Mali-Plot-2. 16-09-2019

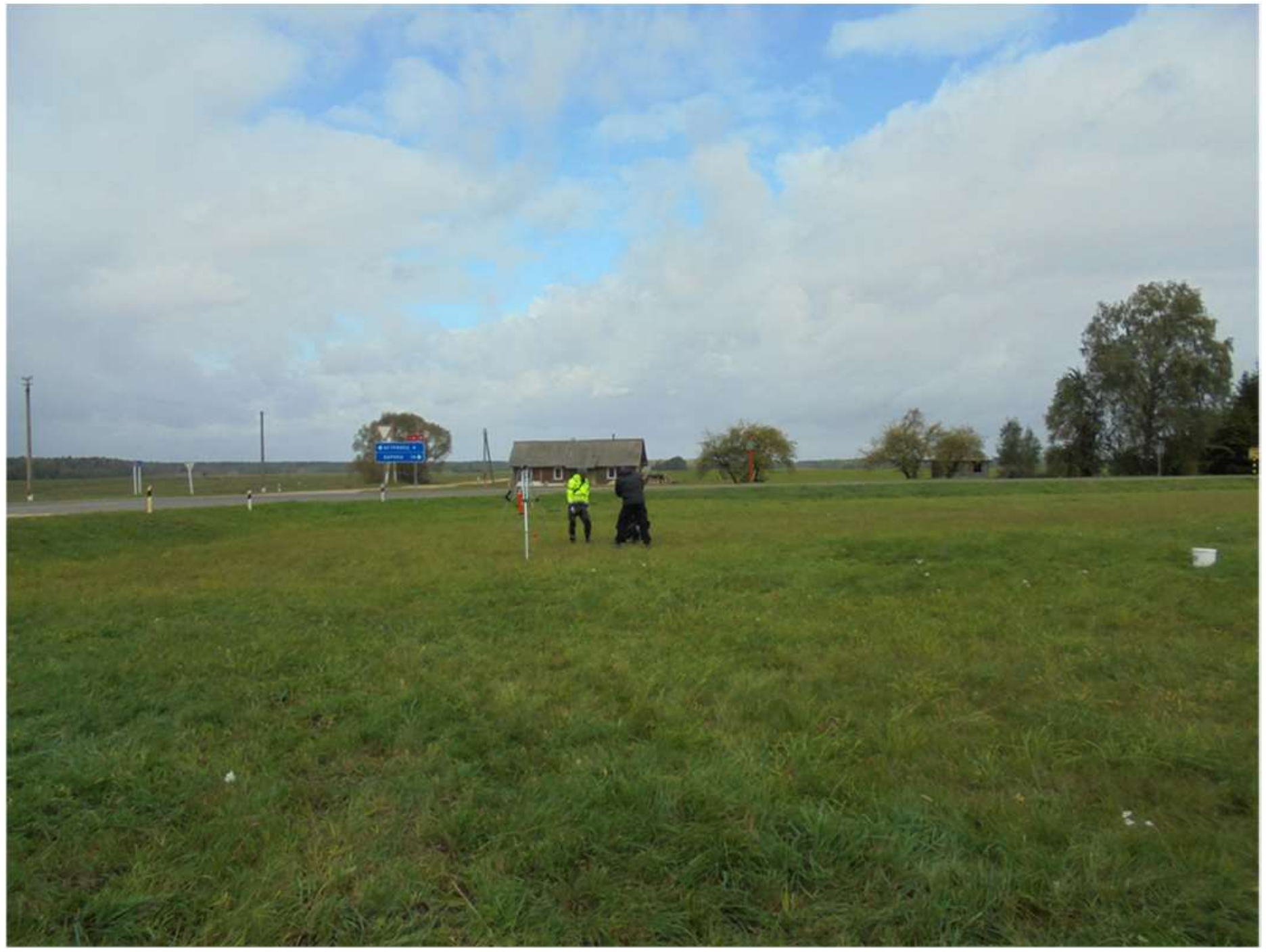

Plots surveyed by the joint expedition of Lund University, Research Institute of Radiation Hygiene and Institute of Radiobiology around BelNPP in 2019.

Edoklani-Plot-3. 16-09-2019

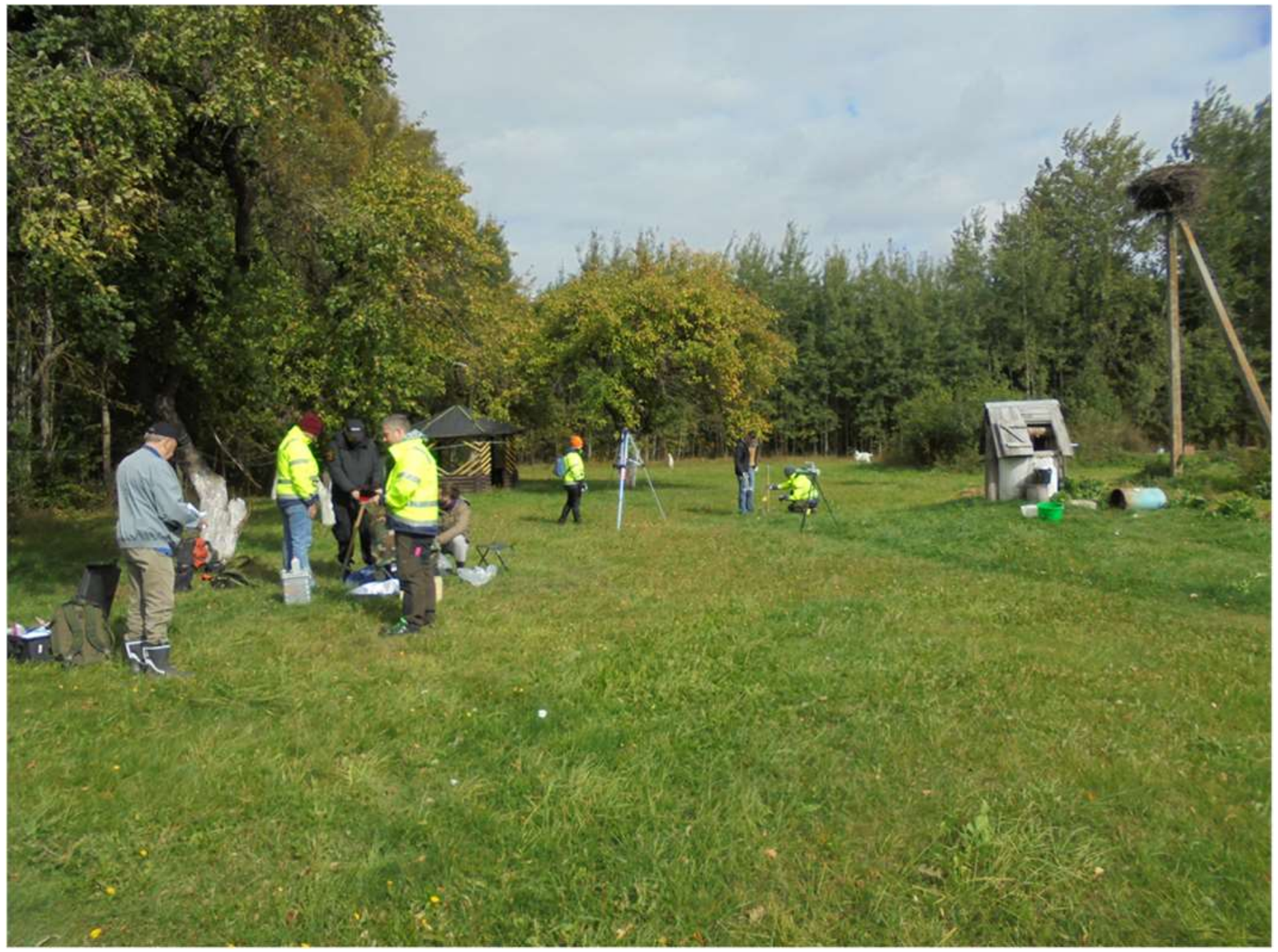

Karveli-Plot-4. 16-09-2019

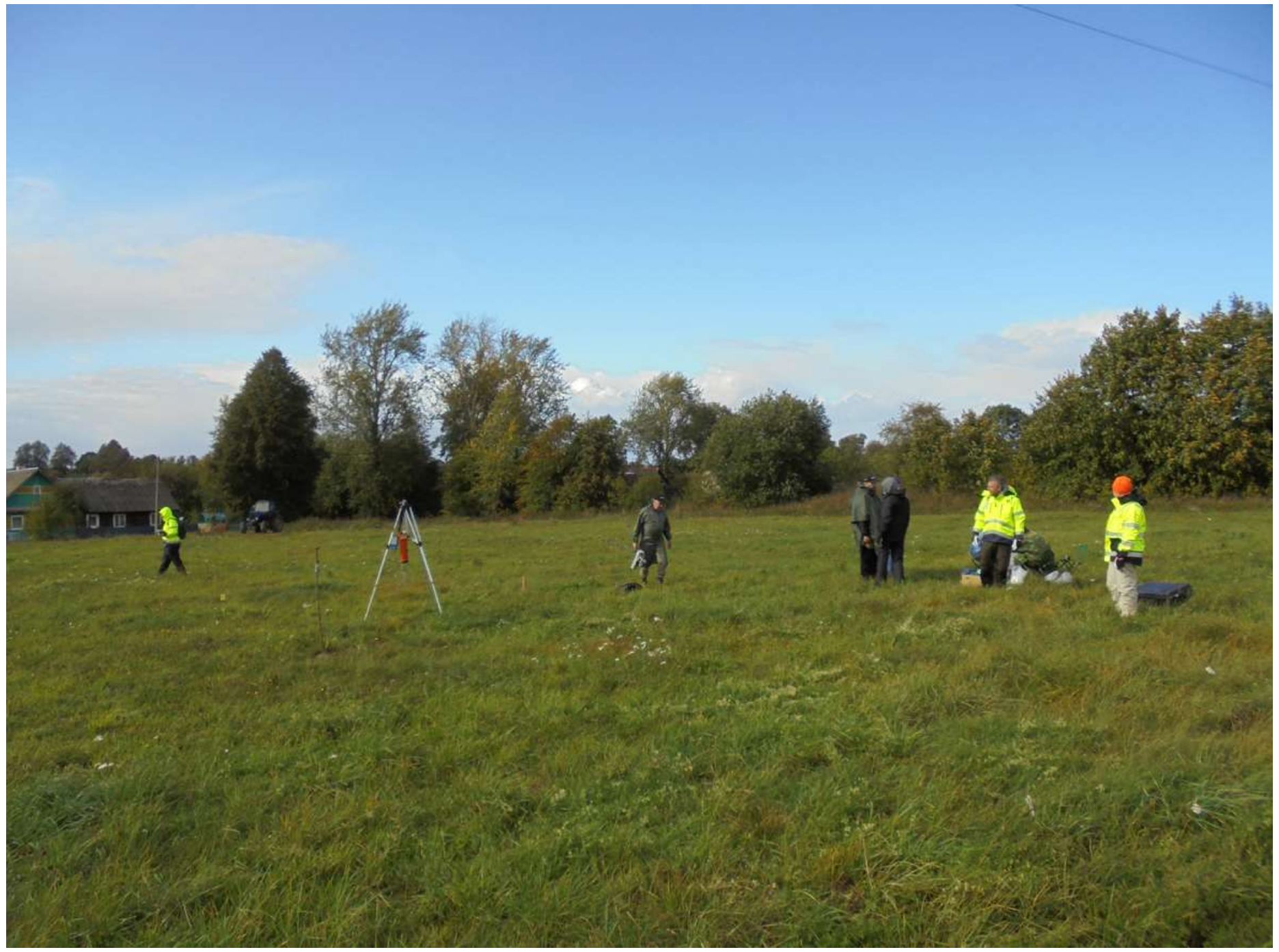

Plots surveyed by the joint expedition of Lund University, Research Institute of Radiation Hygiene and Institute of Radiobiology around BelNPP in 2019.

Lynkishki-Plot-5. 16-09-2019.

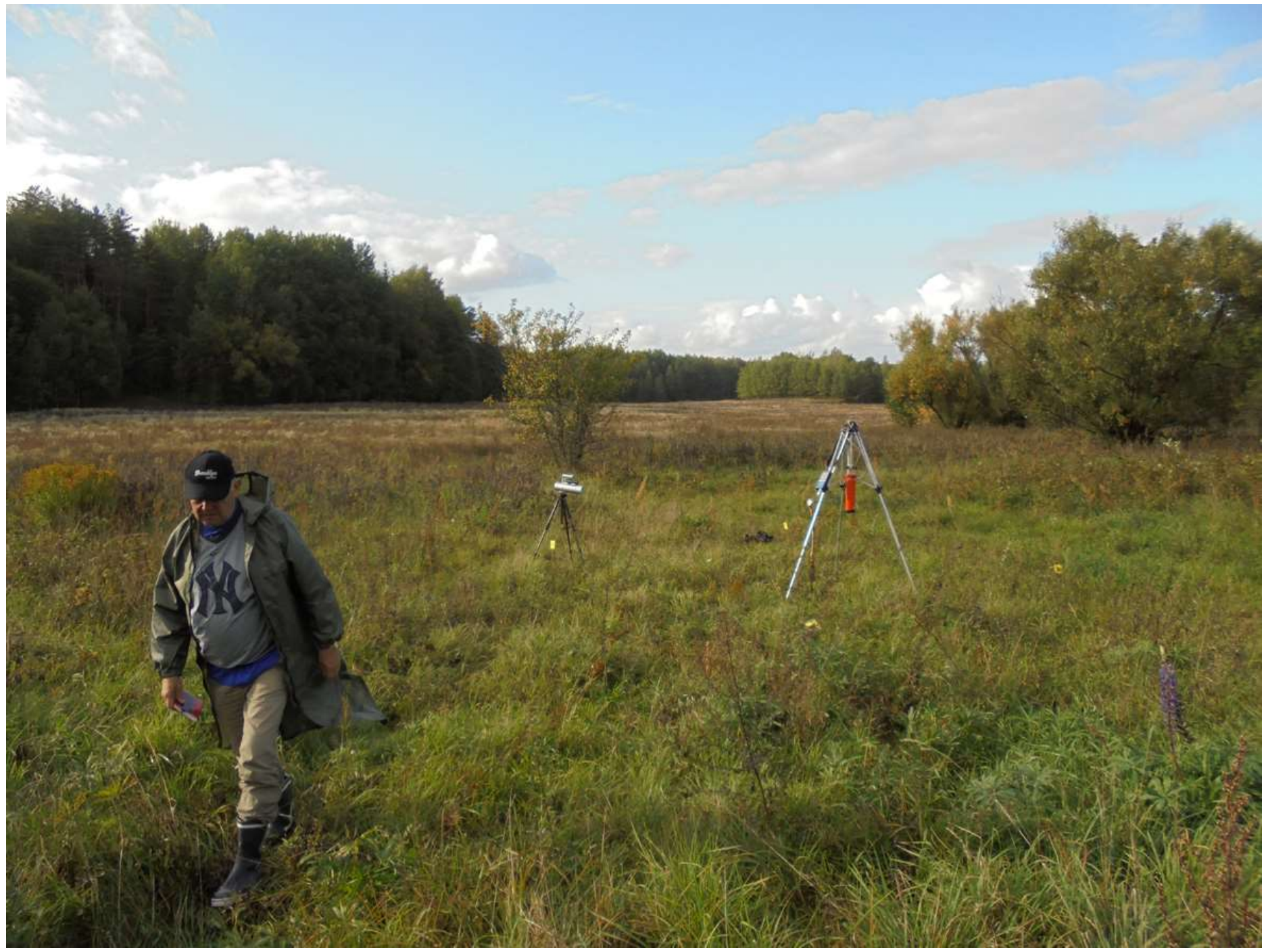

Trokeniki-Plot-6. 17-09-2019.

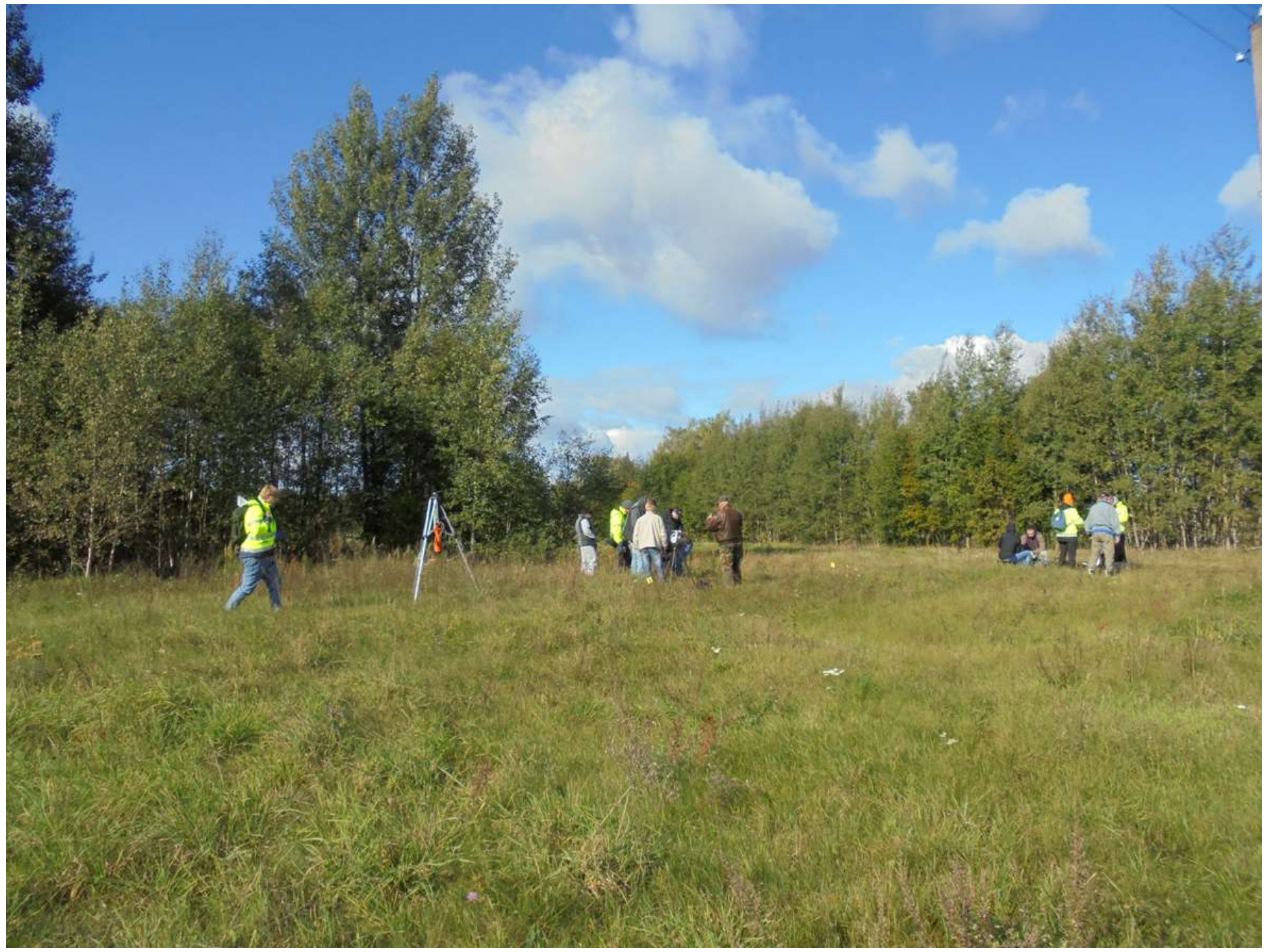

Plots surveyed by the joint expedition of Lund University, Research Institute of Radiation Hygiene and Institute of Radiobiology around BelNPP in 2019.

Vorona-Plot-7. 17-09-2019.

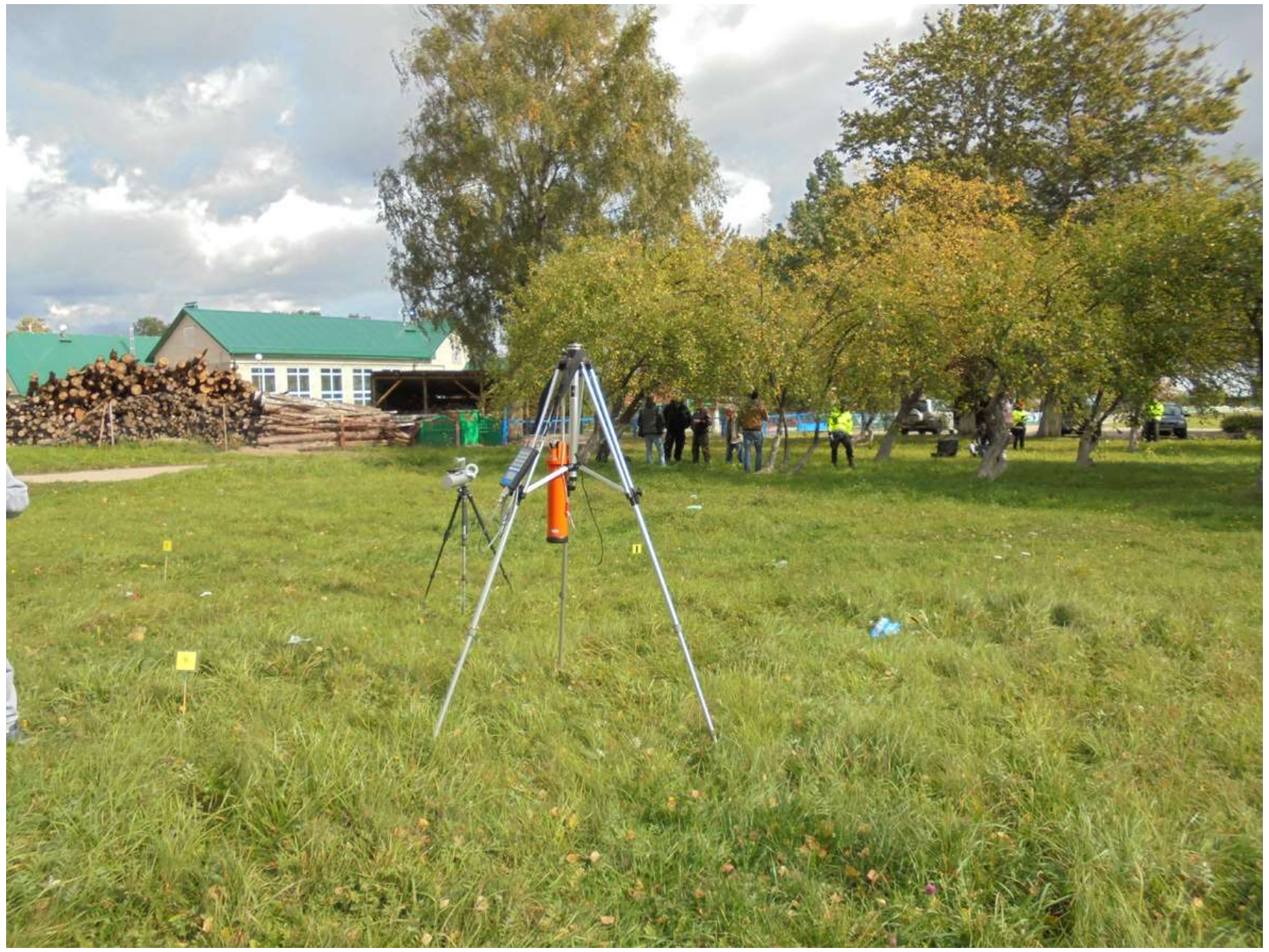

Berezovka-Plot-8. 17-09-2019.

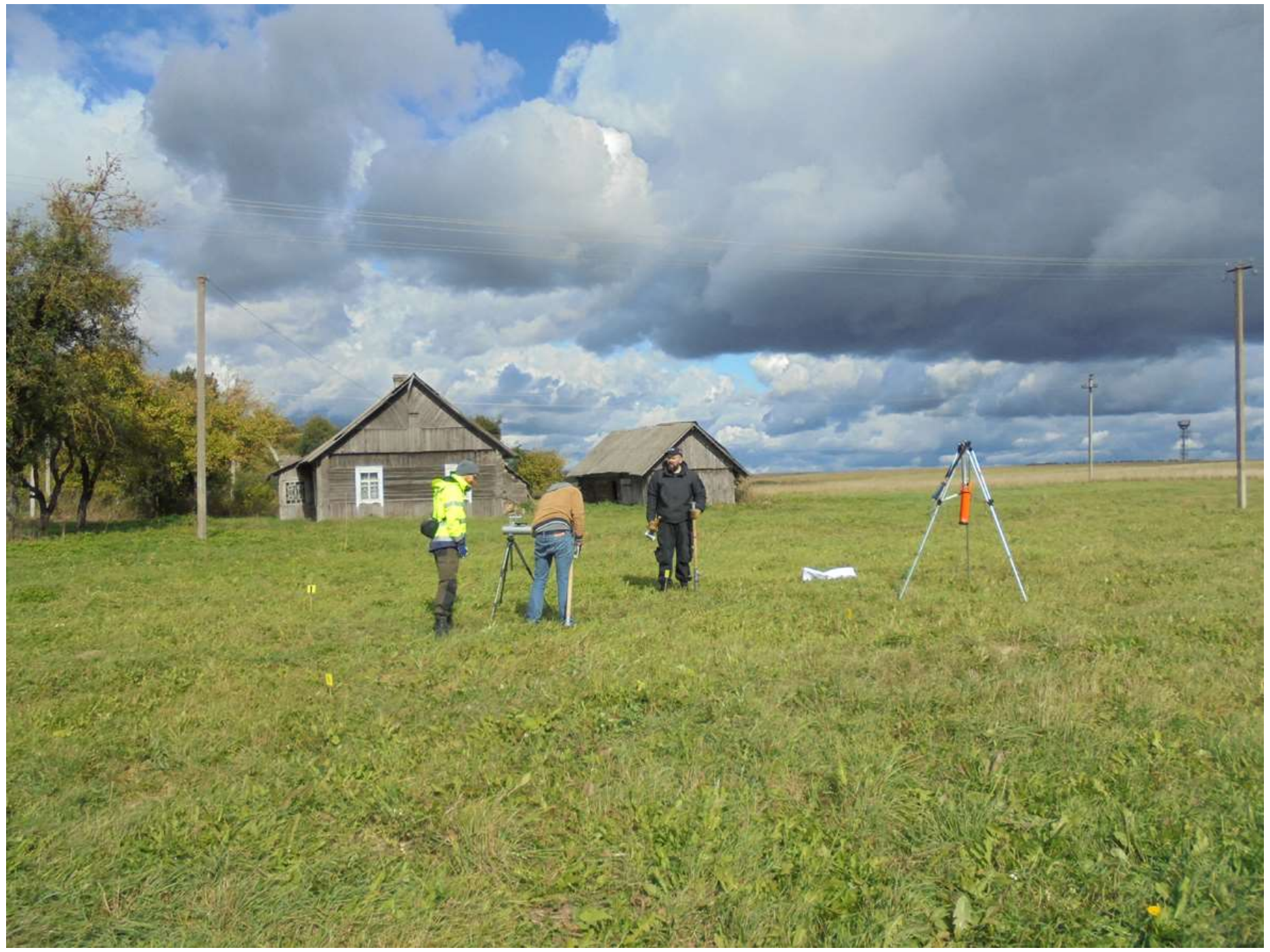

Plots surveyed by the joint expedition of Lund University, Research Institute of Radiation Hygiene and Institute of Radiobiology around BelNPP in 2019.

Goza-Plot-9. 17-09-2019.

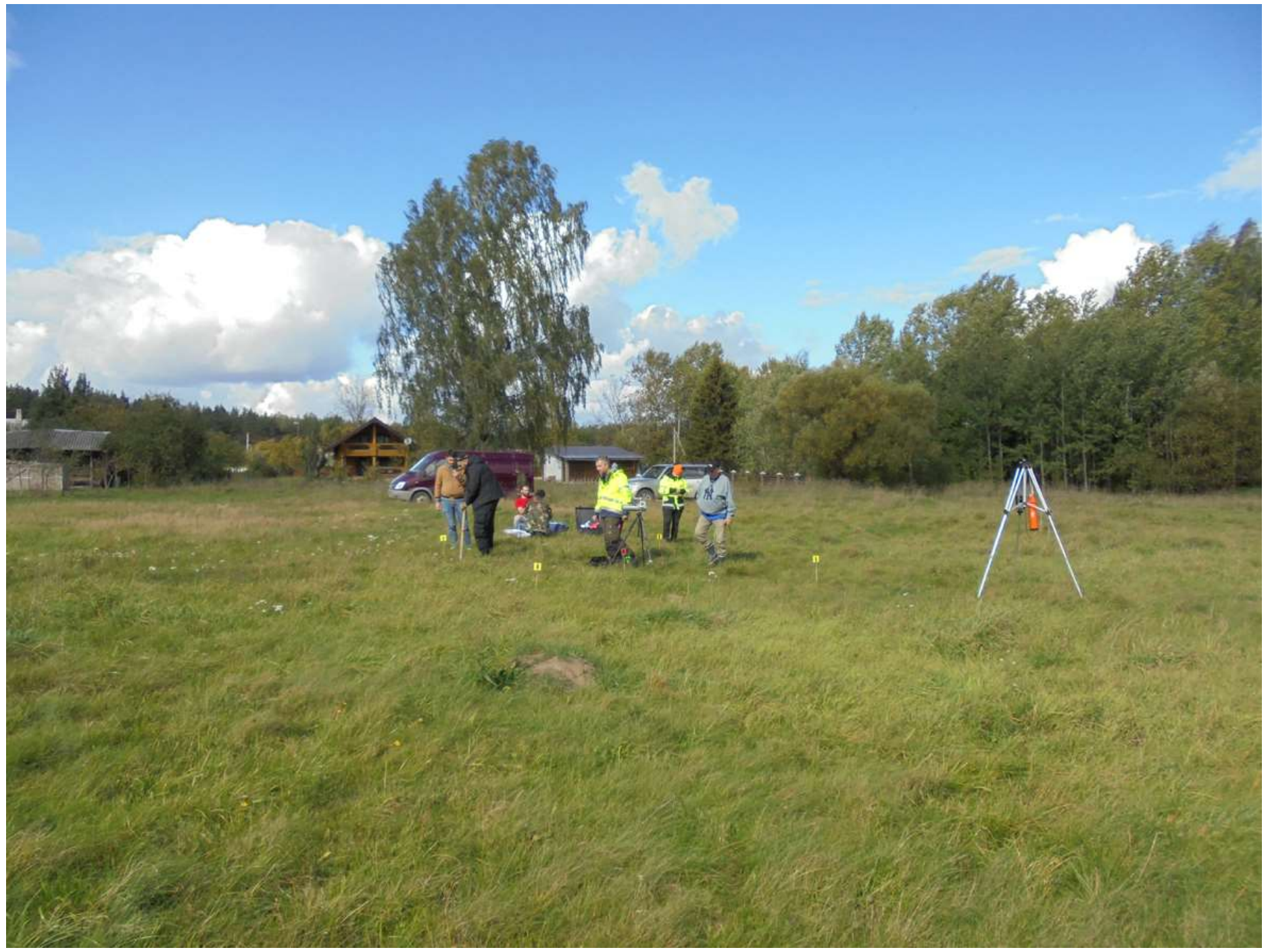

Meshlyany-Plot-10. 17-09-2019.

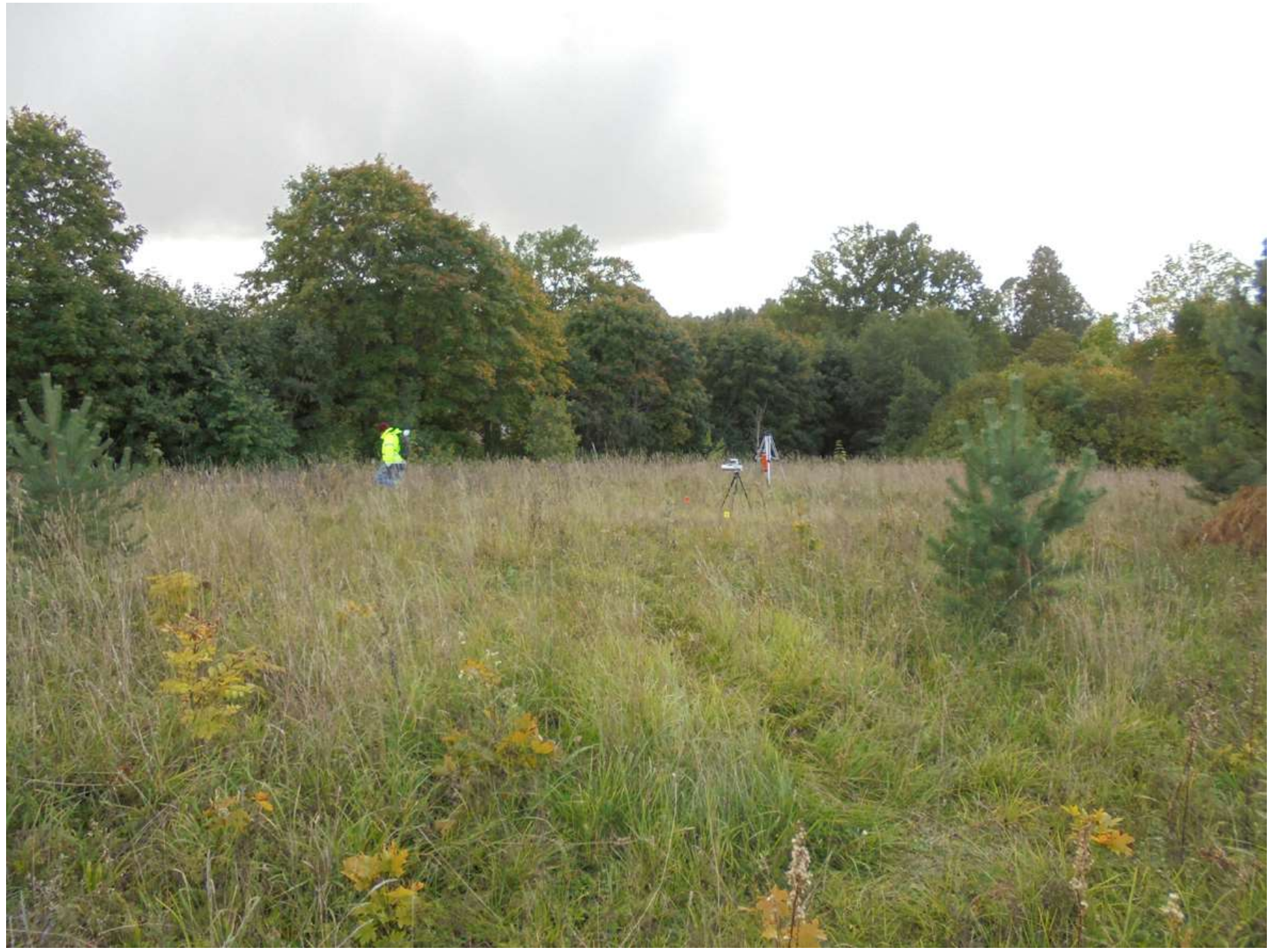

Plots surveyed by the joint expedition of Lund University, Research Institute of Radiation Hygiene and Institute of Radiobiology around BelNPP in 2019.

Mikhalishki-Plot-11. 17-09-2019.

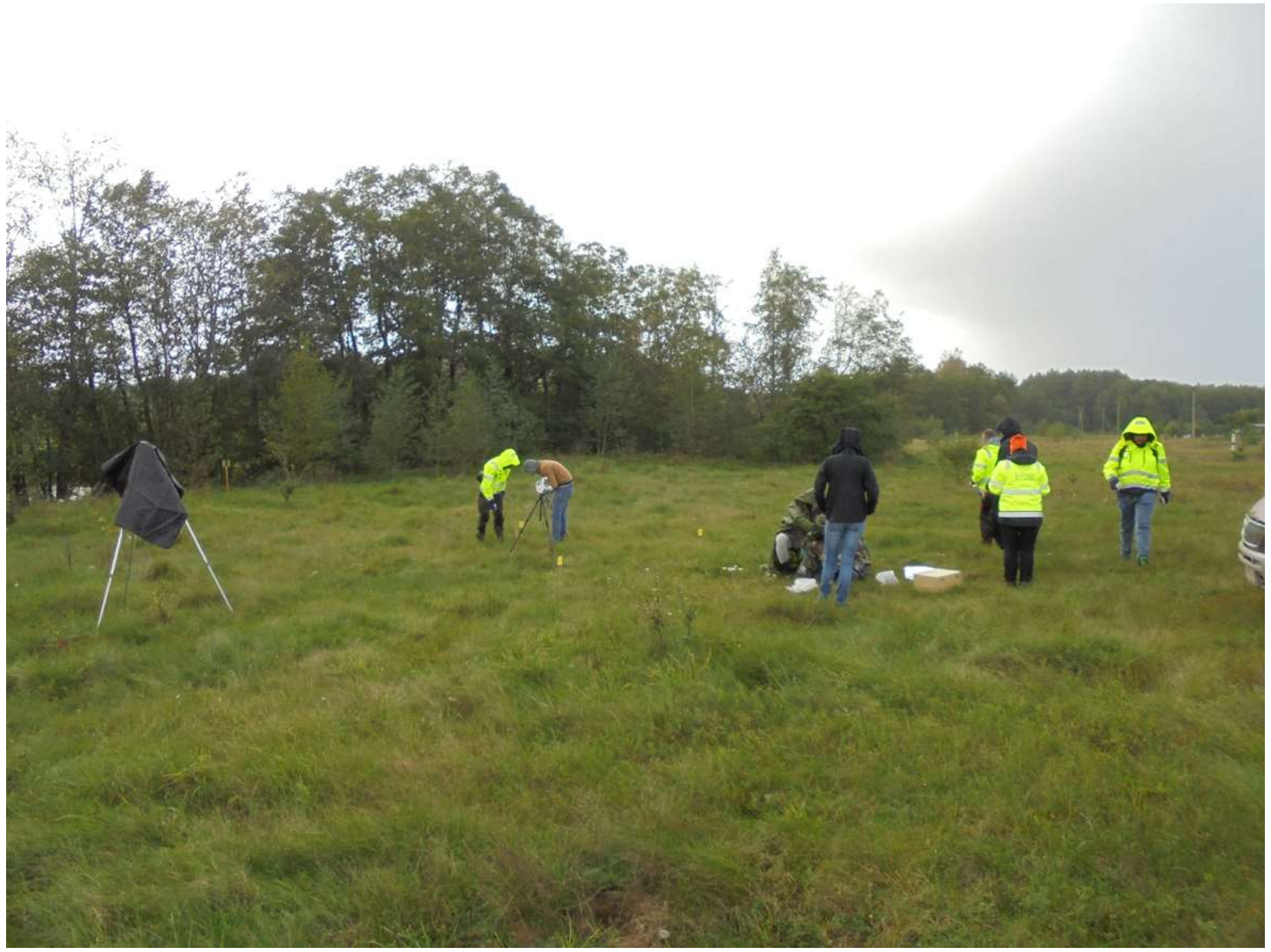

Sidorishki-Plot-12. 18-09-2019.

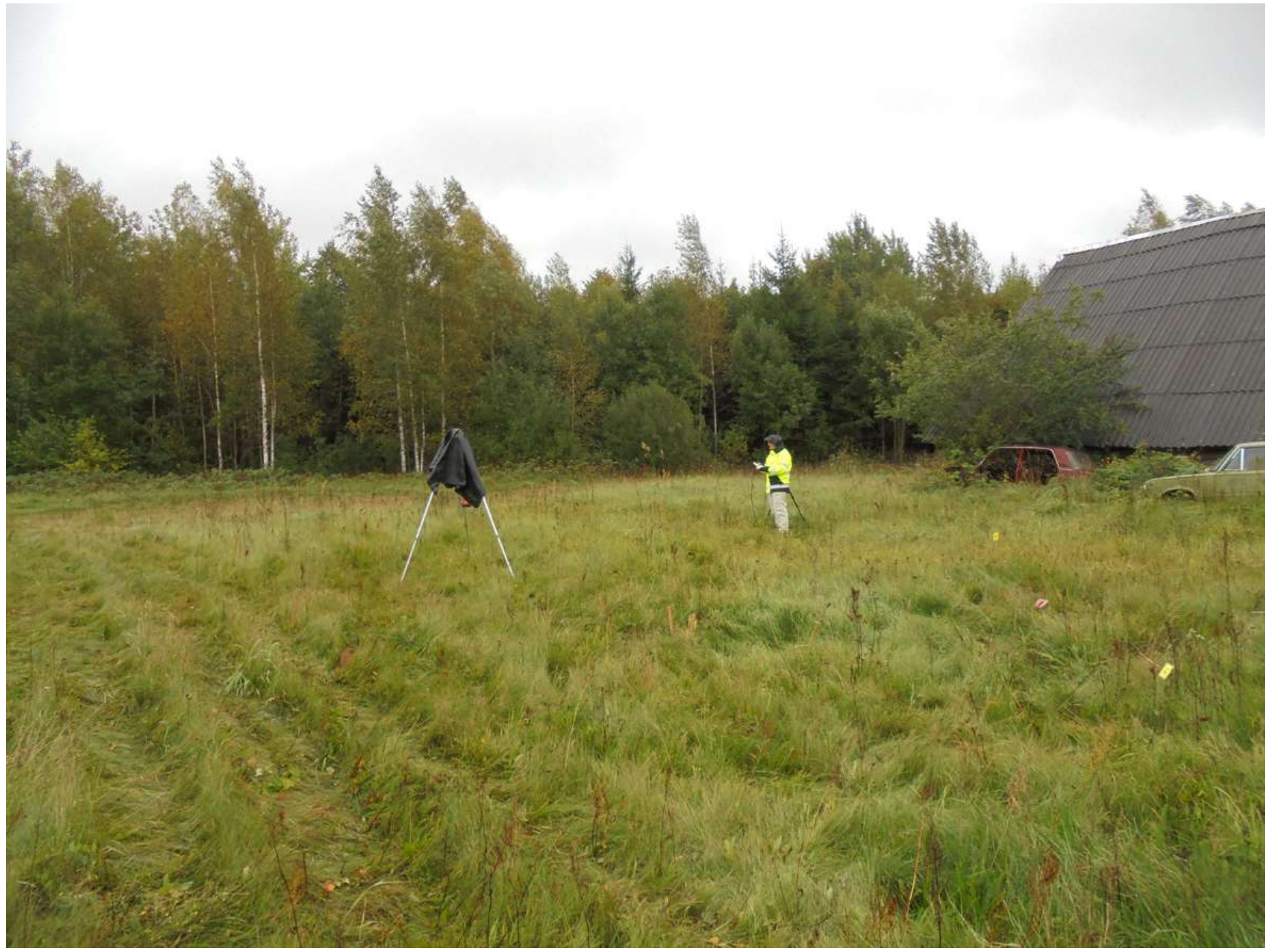

Plots surveyed by the joint expedition of Lund University, Research Institute of Radiation Hygiene and Institute of Radiobiology around BelNPP in 2019.

Olhovka-Plot-13. 18-09-2019.

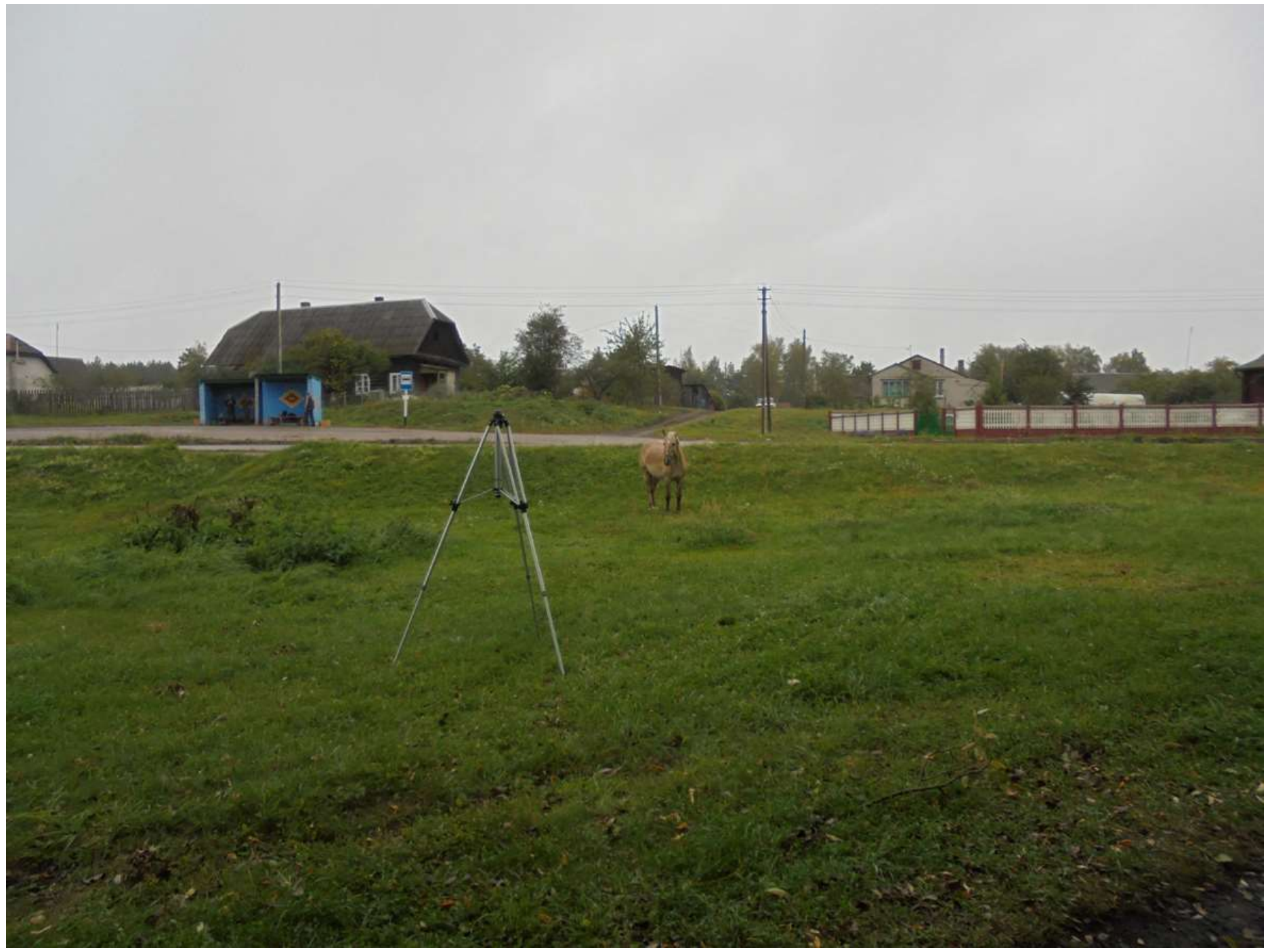

Supronenty-Plot-14. 18-09-2019.

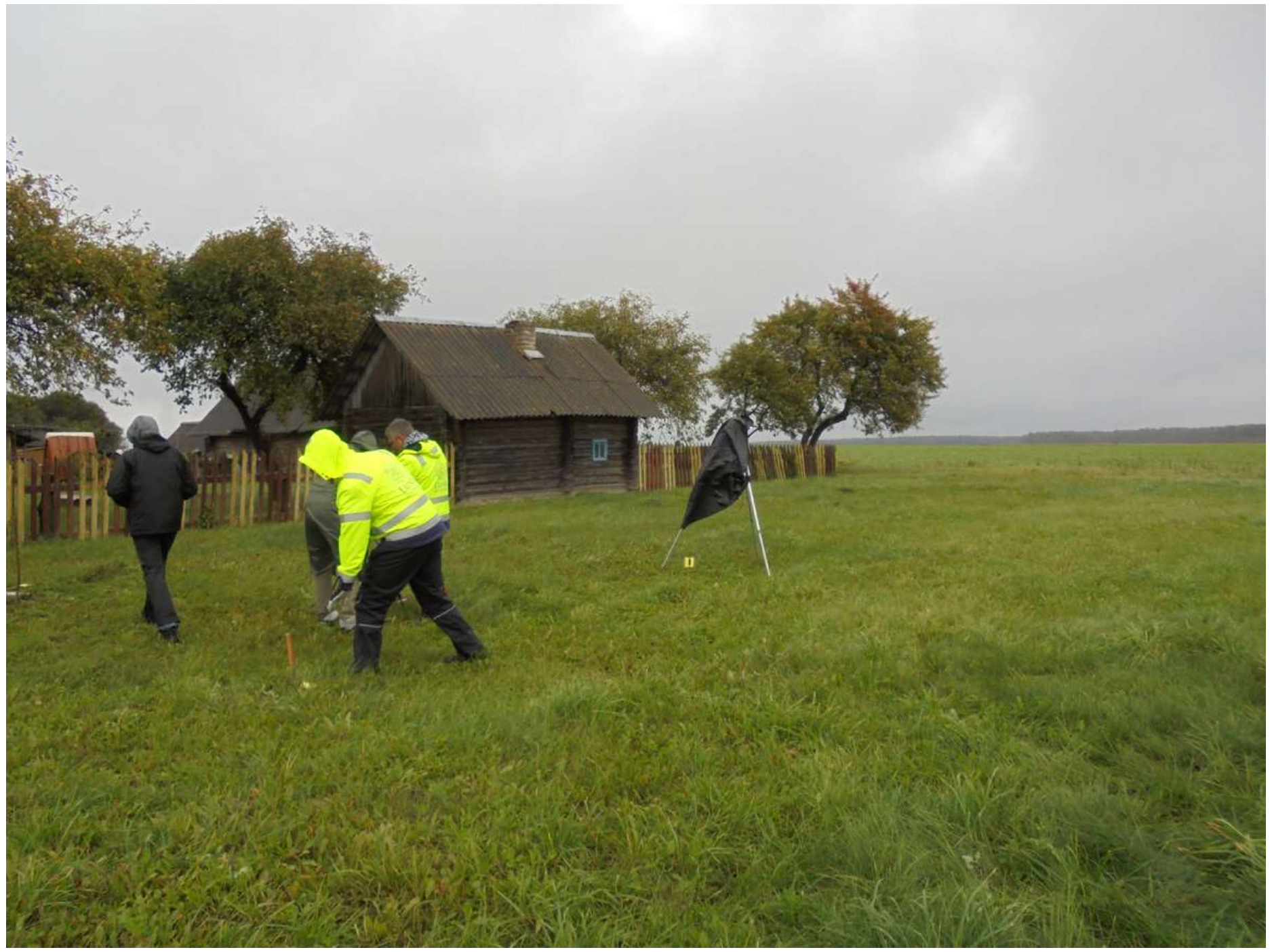

Plots surveyed by the joint expedition of Lund University, Research Institute of Radiation Hygiene and Institute of Radiobiology around BelNPP in 2019.

Markuny-Plot-15. 18-09-2019.

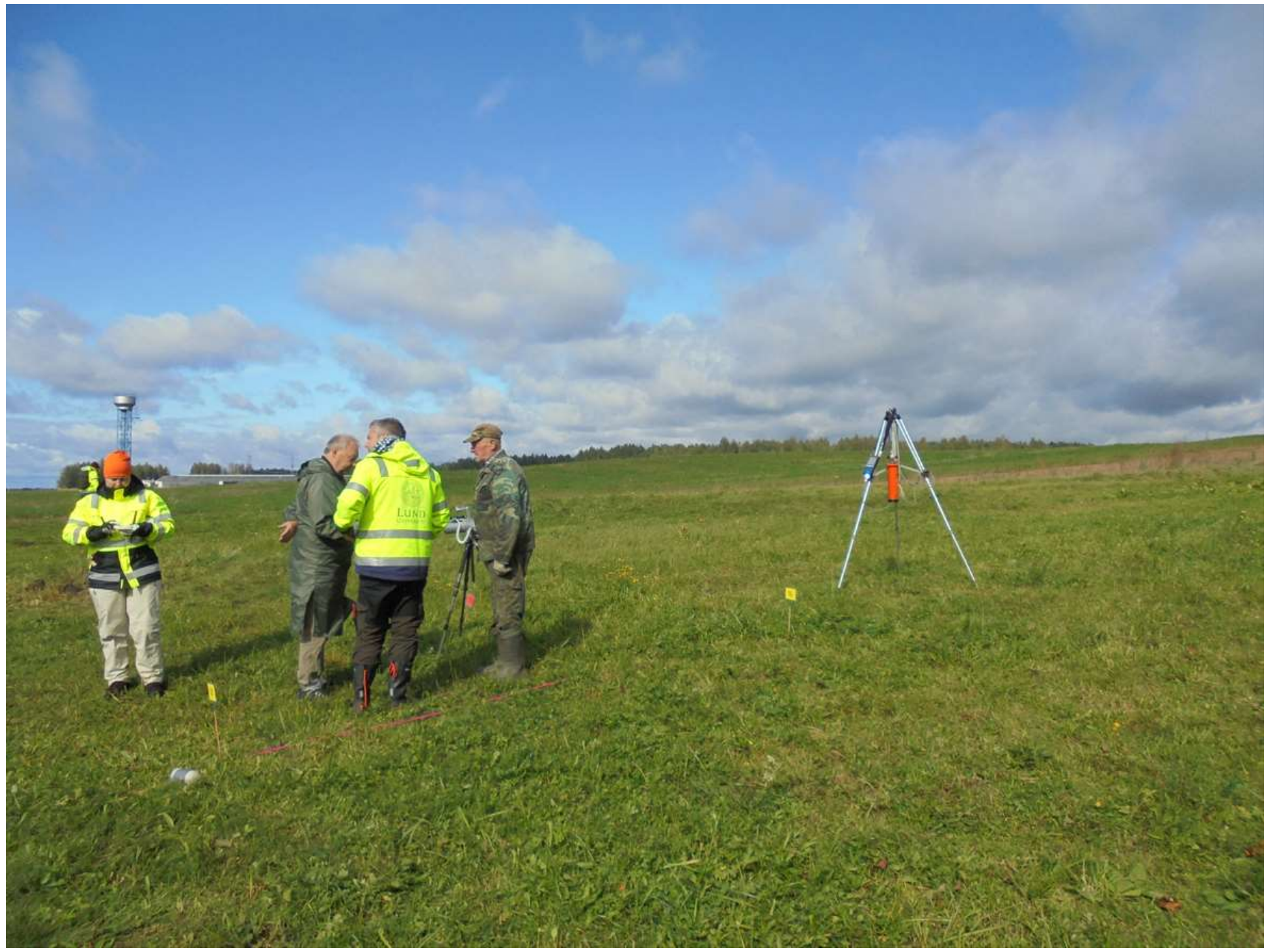

Skerdimy-Plot-16. 18-09-2019.

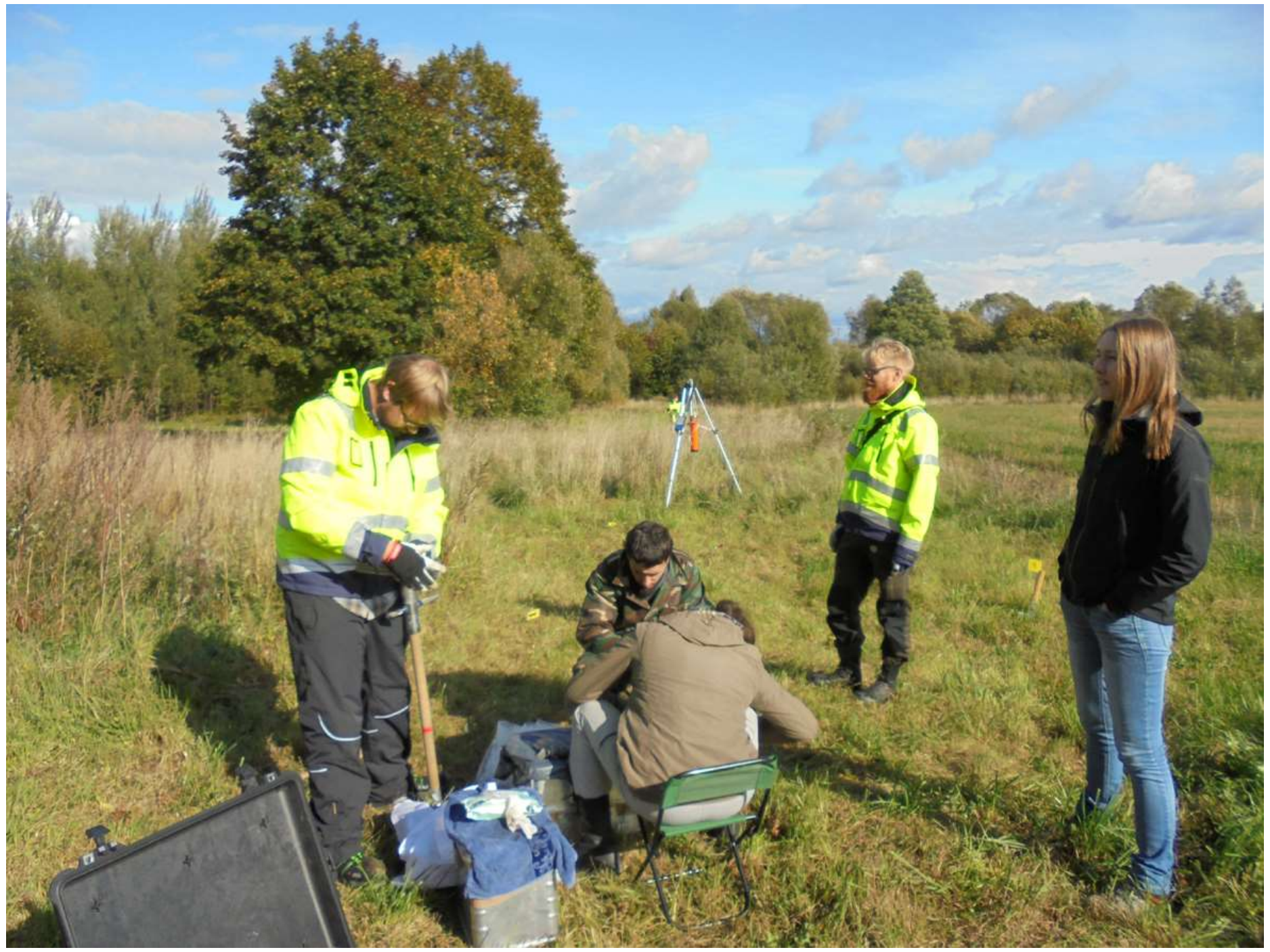

Plots surveyed by the joint expedition of Lund University, Research Institute of Radiation Hygiene and Institute of Radiobiology around BelNPP in 2019.

Chehi-Plot-17. 18-09-2018.

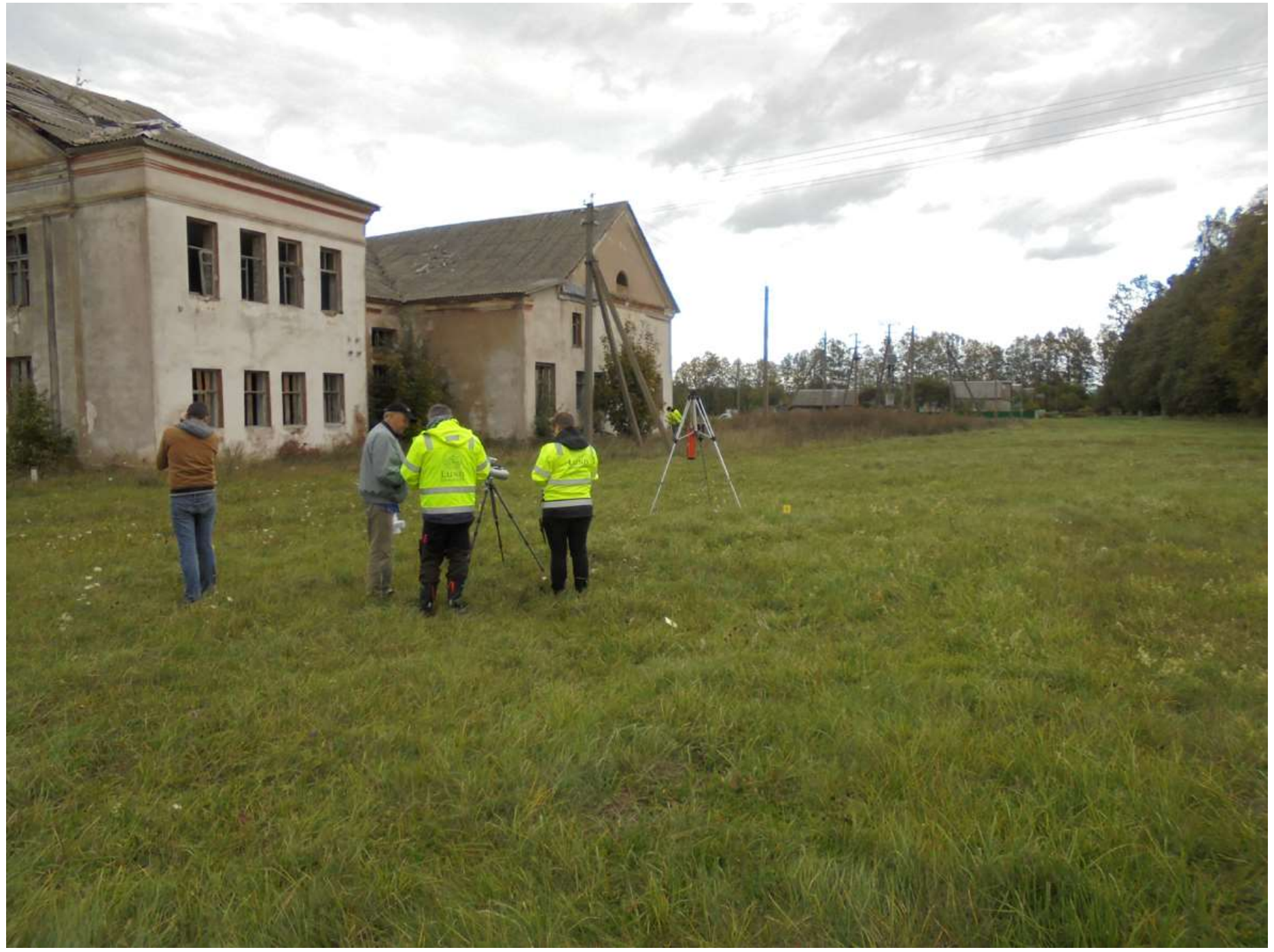

Gaigoli-Plot-18. 18-09-2019.

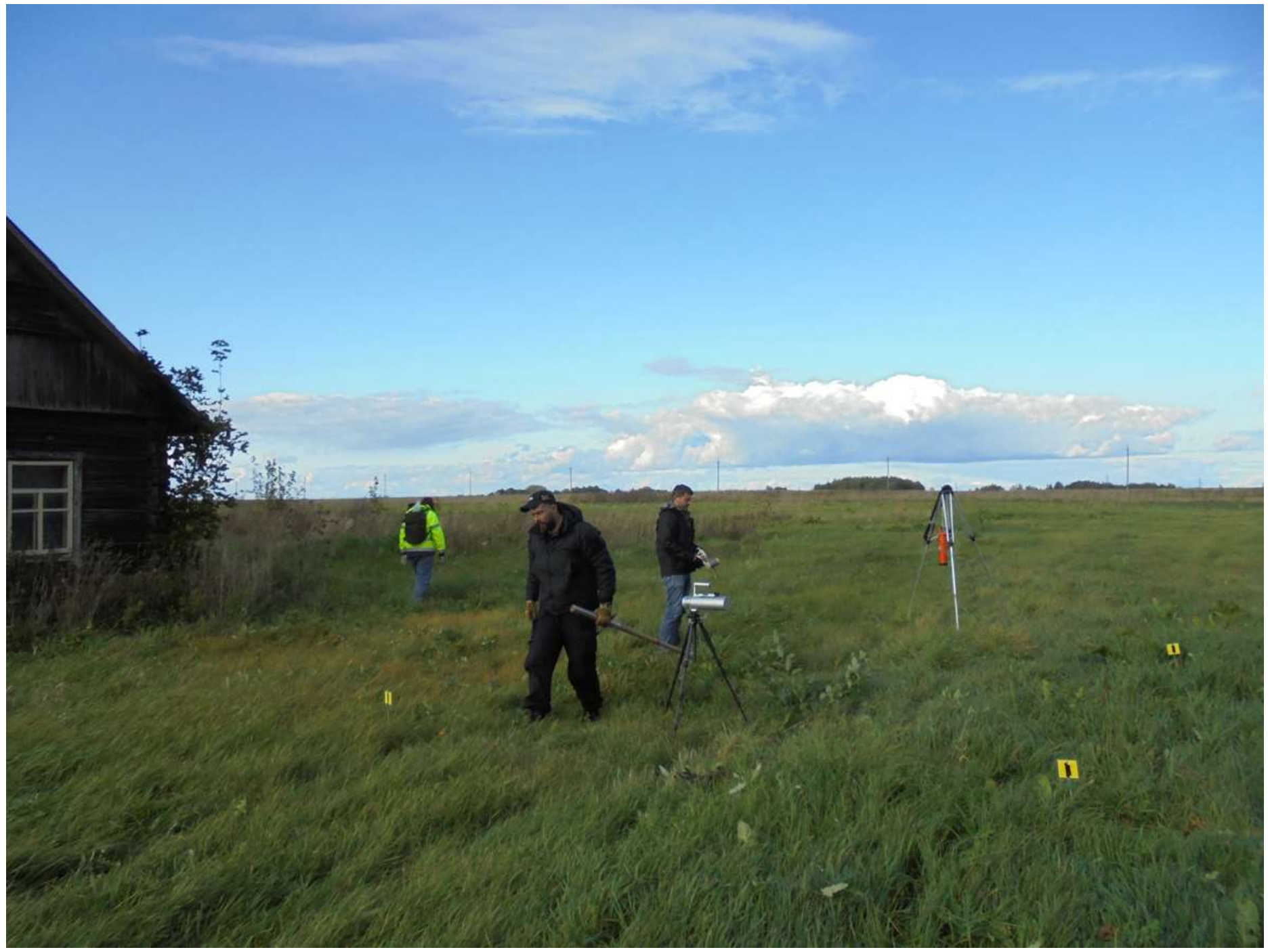

Plots surveyed by the joint expedition of Lund University, Research Institute of Radiation Hygiene and Institute of Radiobiology around BelNPP in 2019.

Gelyuny-Plot-19. 19-09-2019.

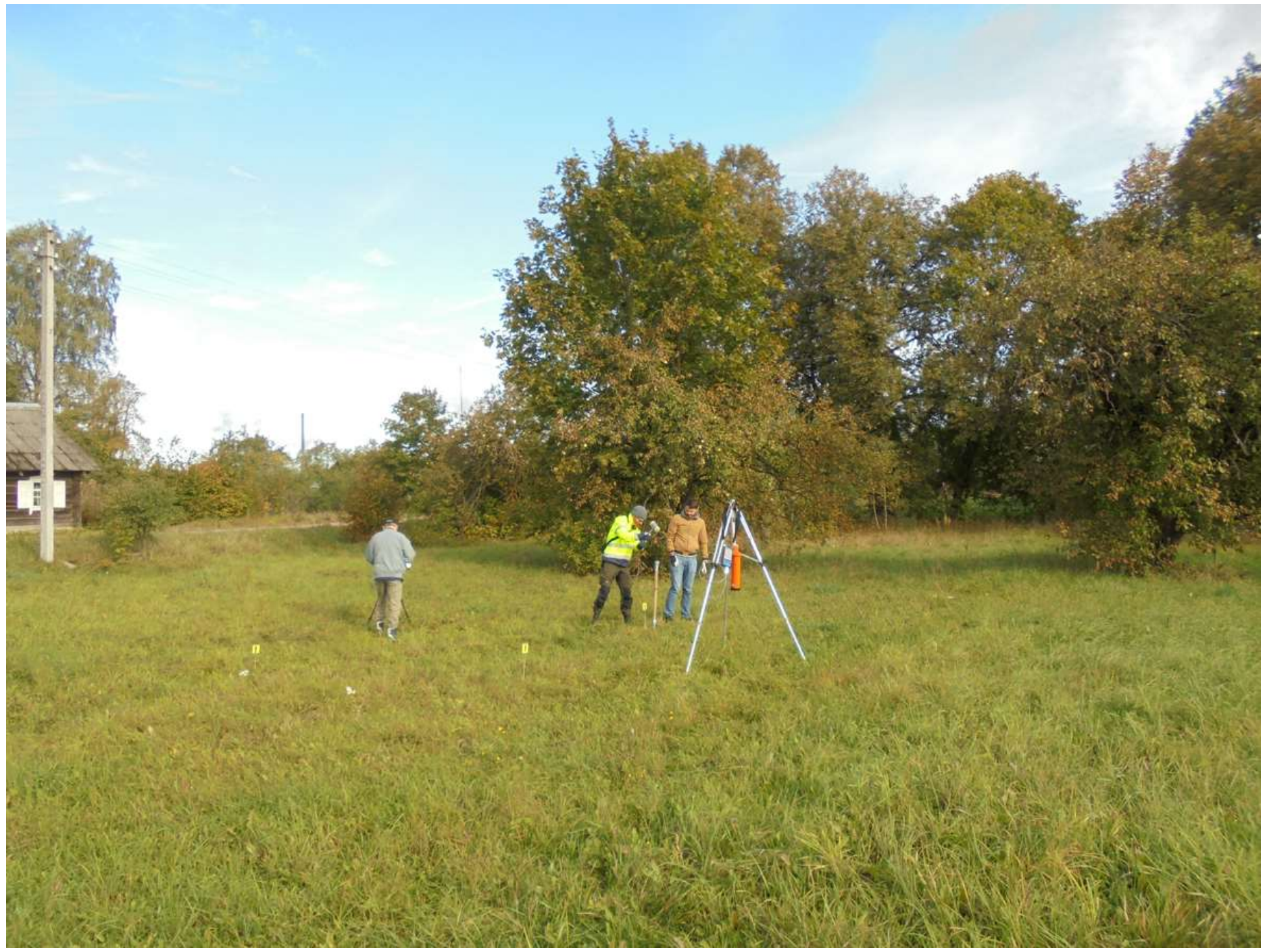

Rymdyuny-Plot-20. 19-09-2019.

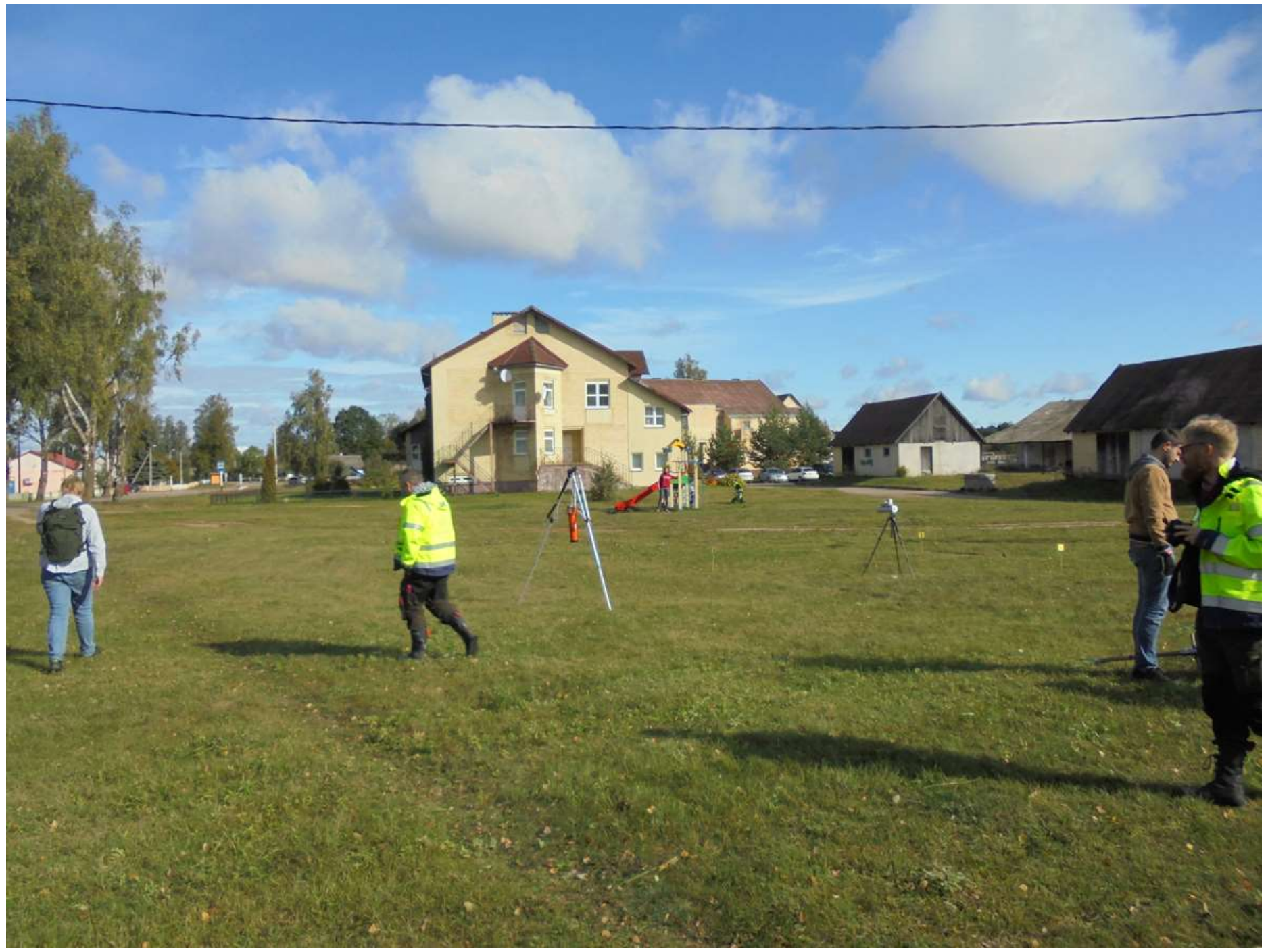

Plots surveyed by the joint expedition of Lund University, Research Institute of Radiation Hygiene and Institute of Radiobiology around BelNPP in 2019.

Gudeniki-Plot-21. 19-09-2019.

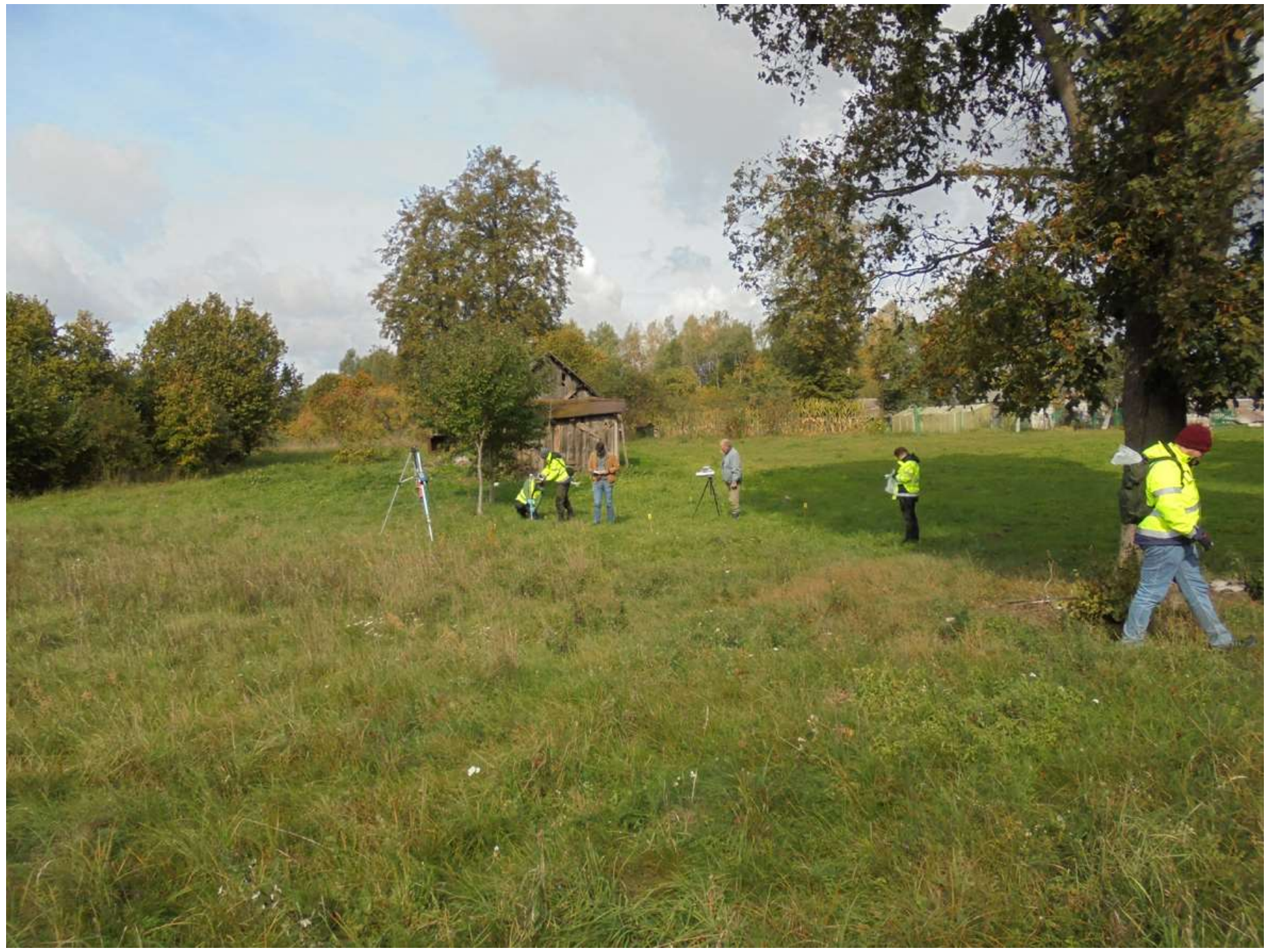

Gervyaty-Plot-22. 19-09-2019.

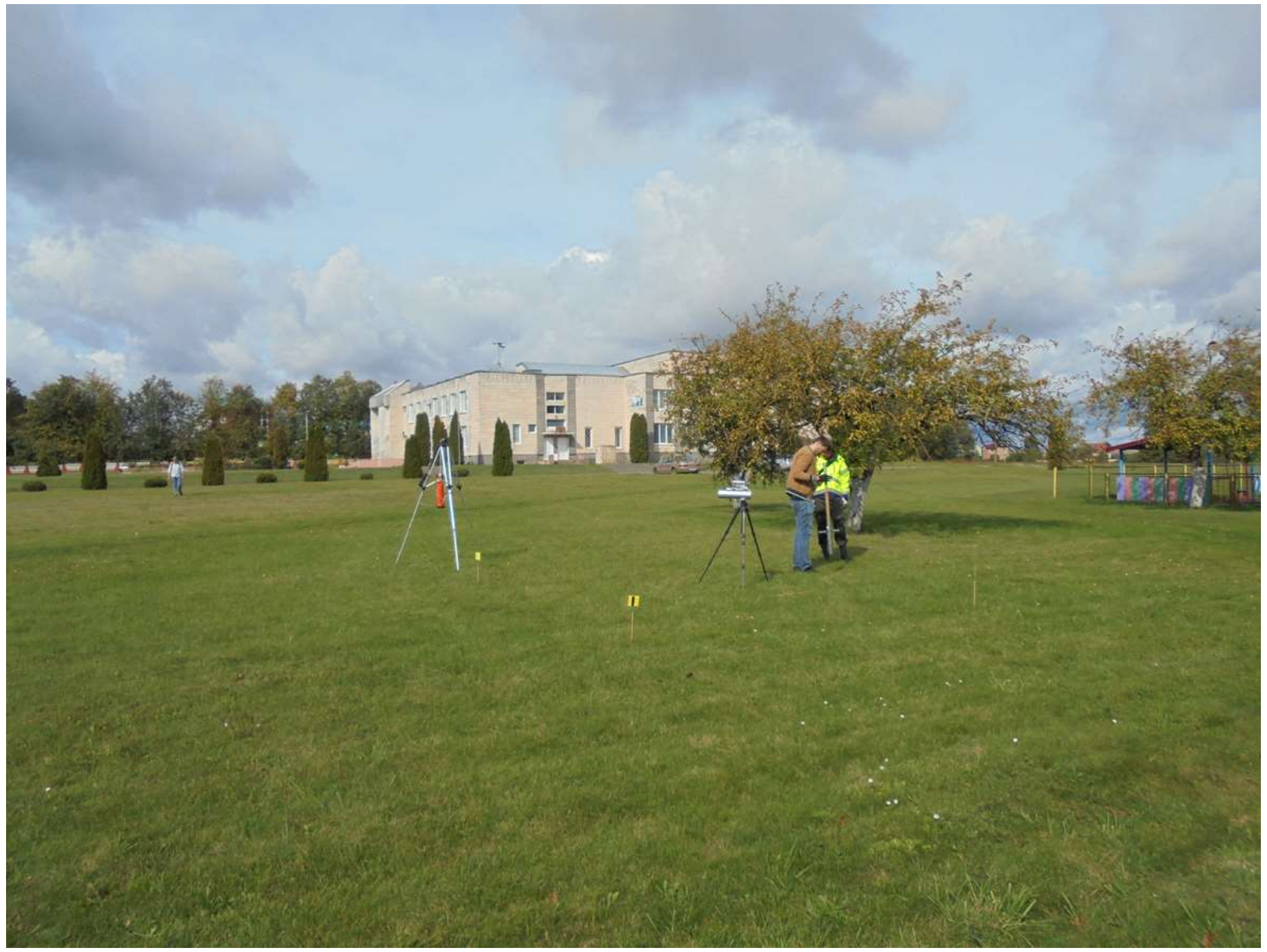

Plots surveyed by the joint expedition of Lund University, Research Institute of Radiation Hygiene and Institute of Radiobiology around BelNPP in 2019.

Galchuny-Plot-23. 19-09-2019.

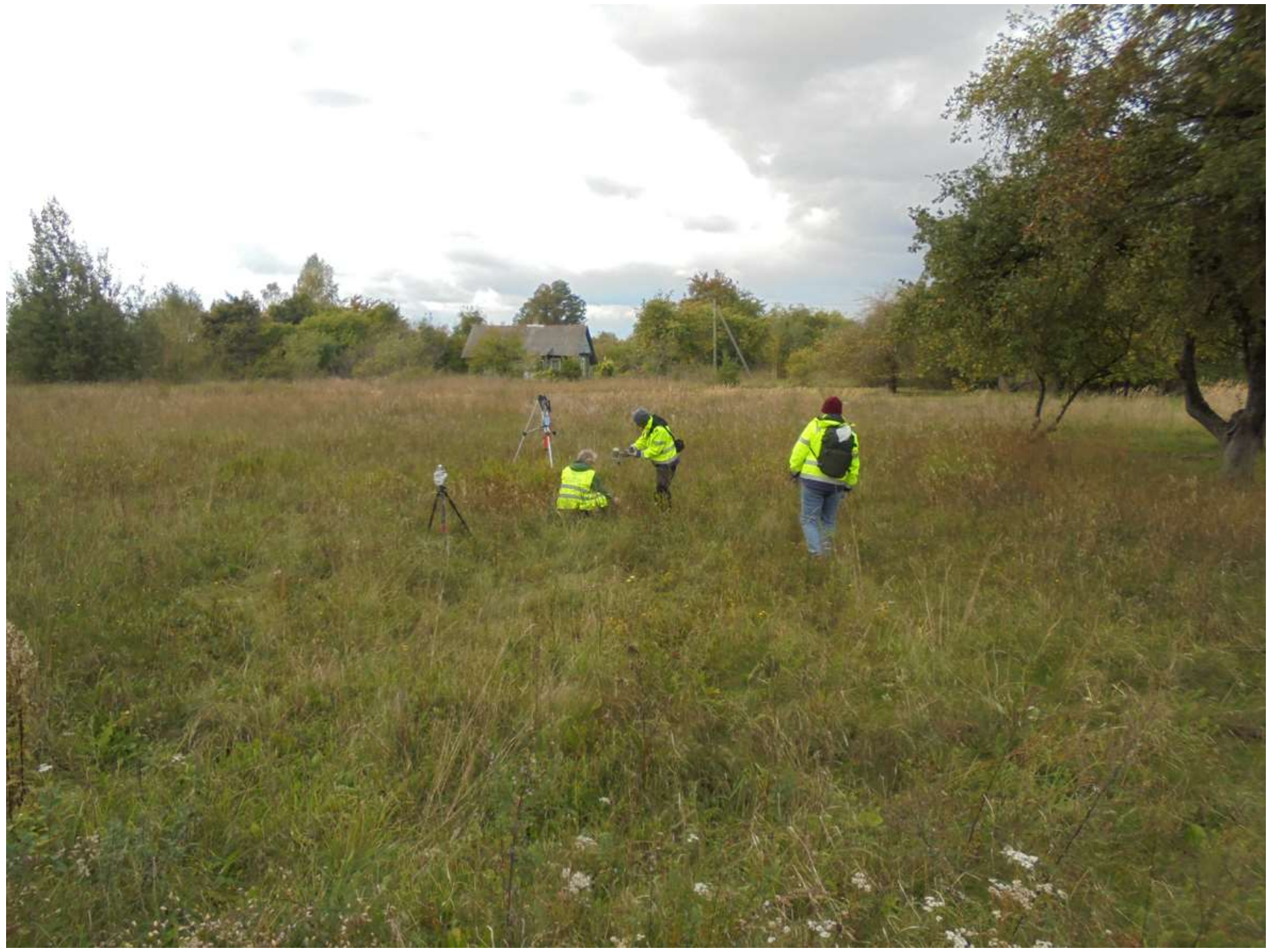

Matski-Plot-24. 19-09-2019.

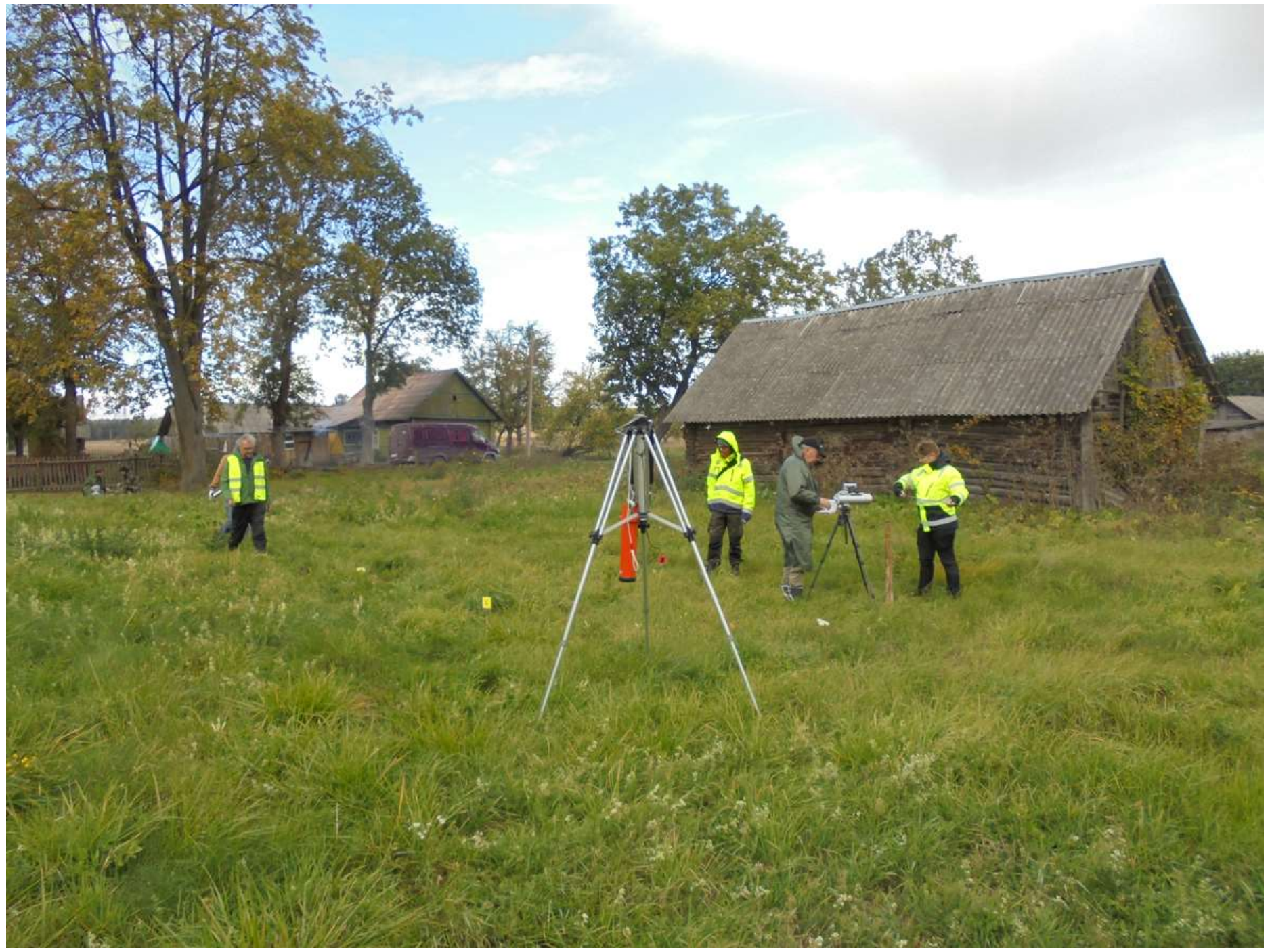

Plots surveyed by the joint expedition of Lund University, Research Institute of Radiation Hygiene and Institute of Radiobiology around BelNPP in 2019.

Chernishki-Plot-25. 19-09-2019.

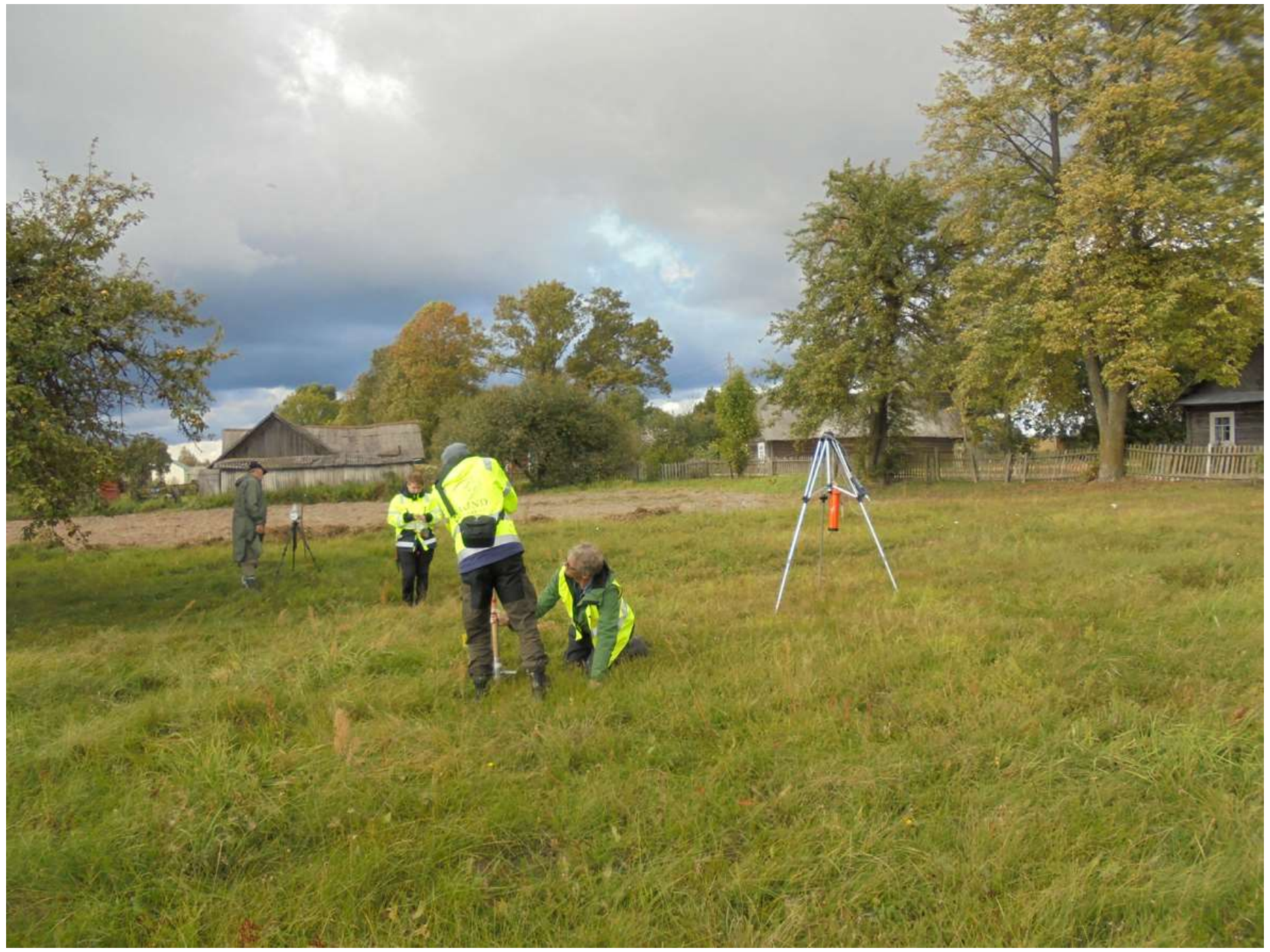

Kernyany-Plot-26. 19-09-2019.

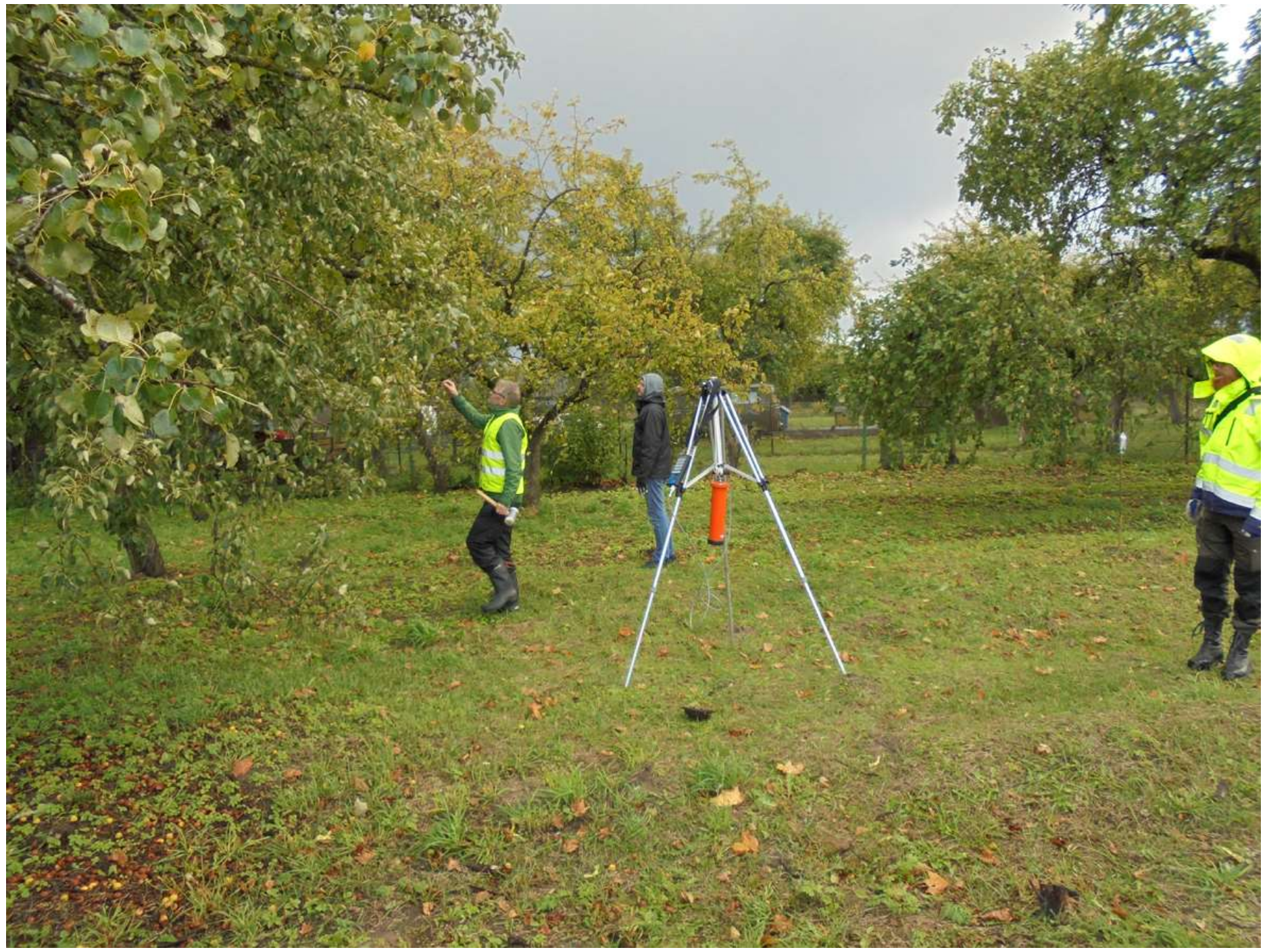

Plots surveyed by the joint expedition of Lund University, Research Institute of Radiation Hygiene and Institute of Radiobiology around BelNPP in 2019.

Filipany-Plot-27. 19-09-2019.

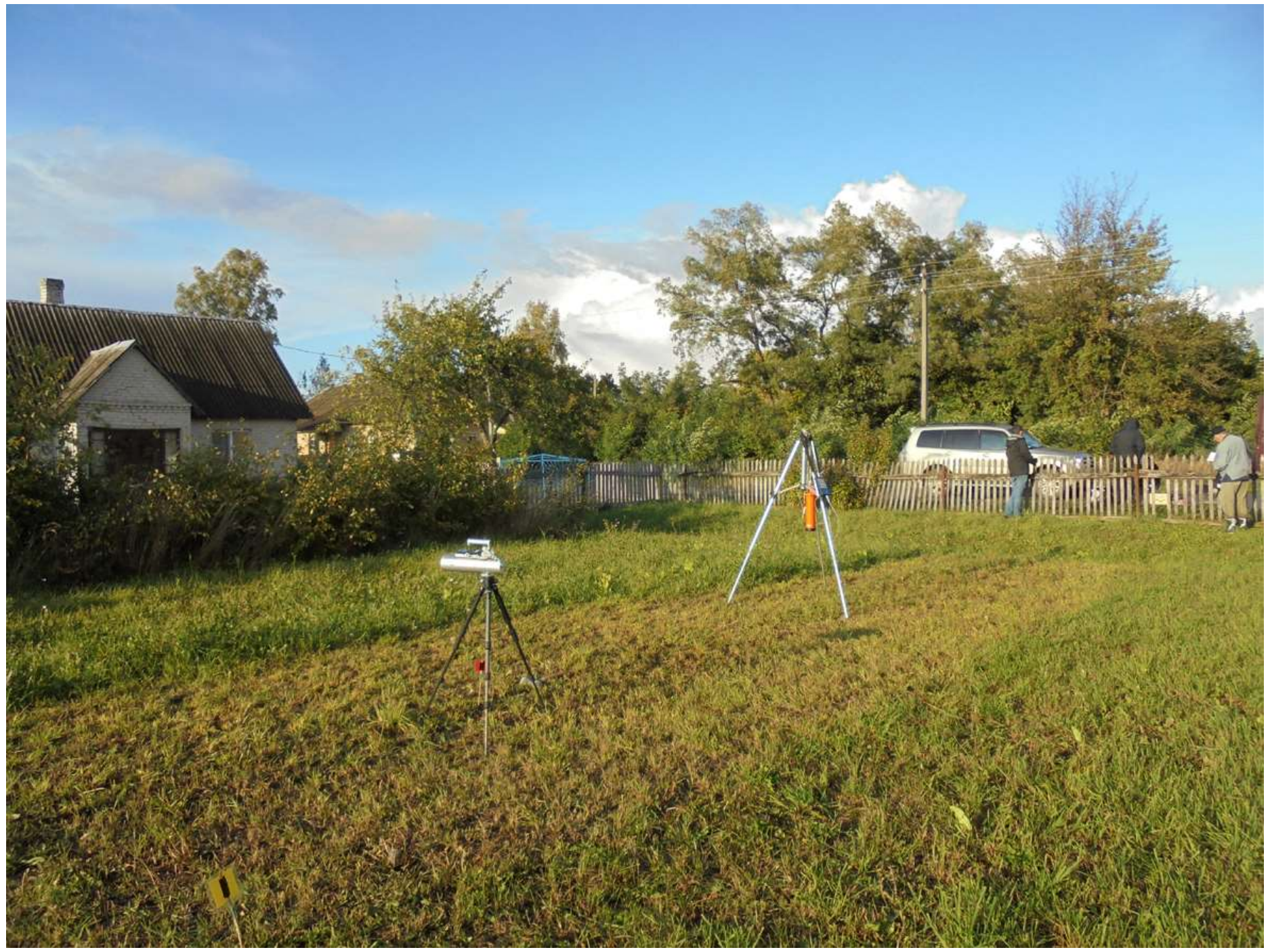

Vornyany-Plot-28. 20-09-2019.

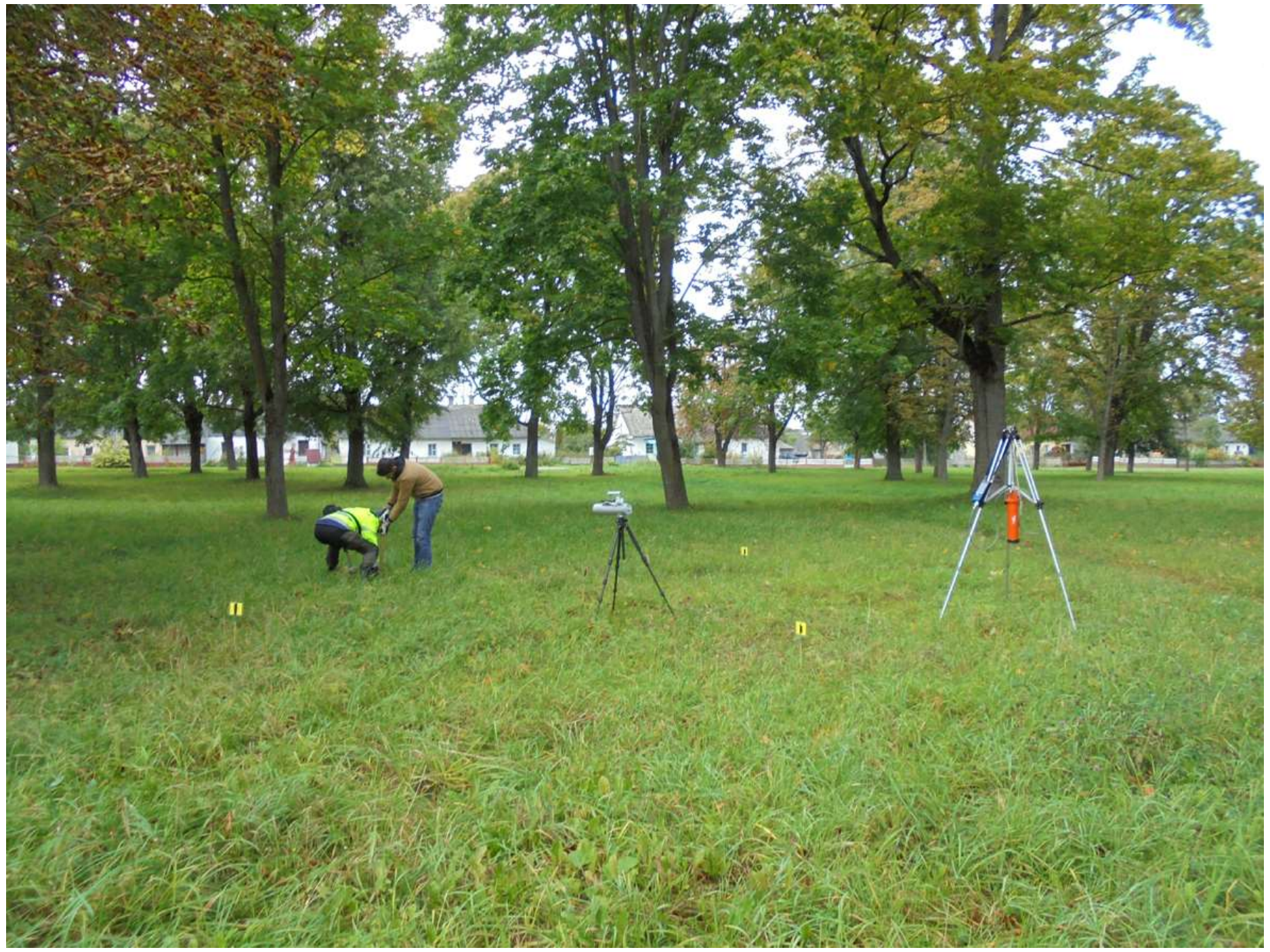

Plots surveyed by the joint expedition of Lund University, Research Institute of Radiation Hygiene and Institute of Radiobiology around BelNPP in 2019.

Bolniki-Plot-29. 20-09-2019.

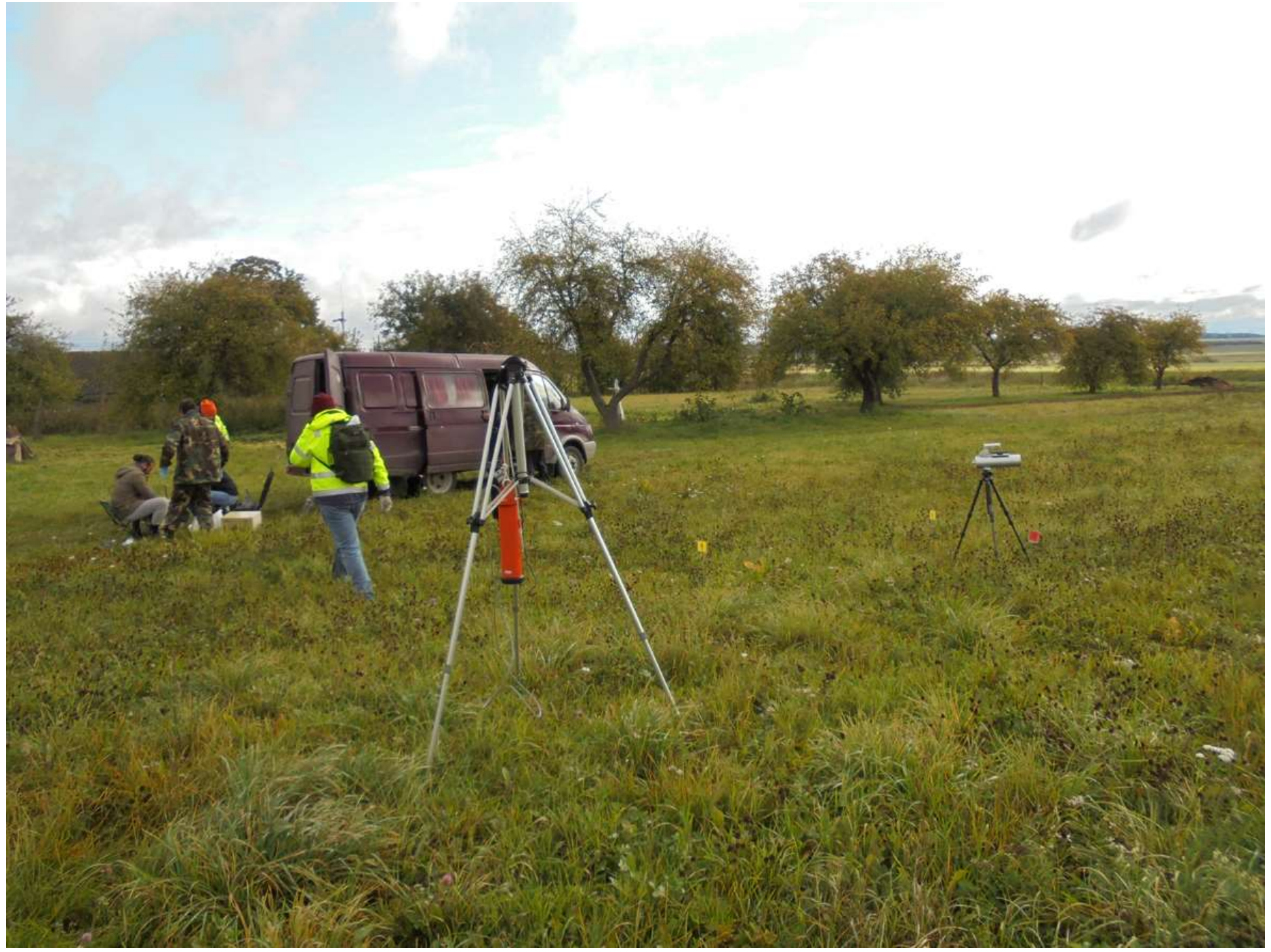

Troschany-Plot-30. 15-10-2019.

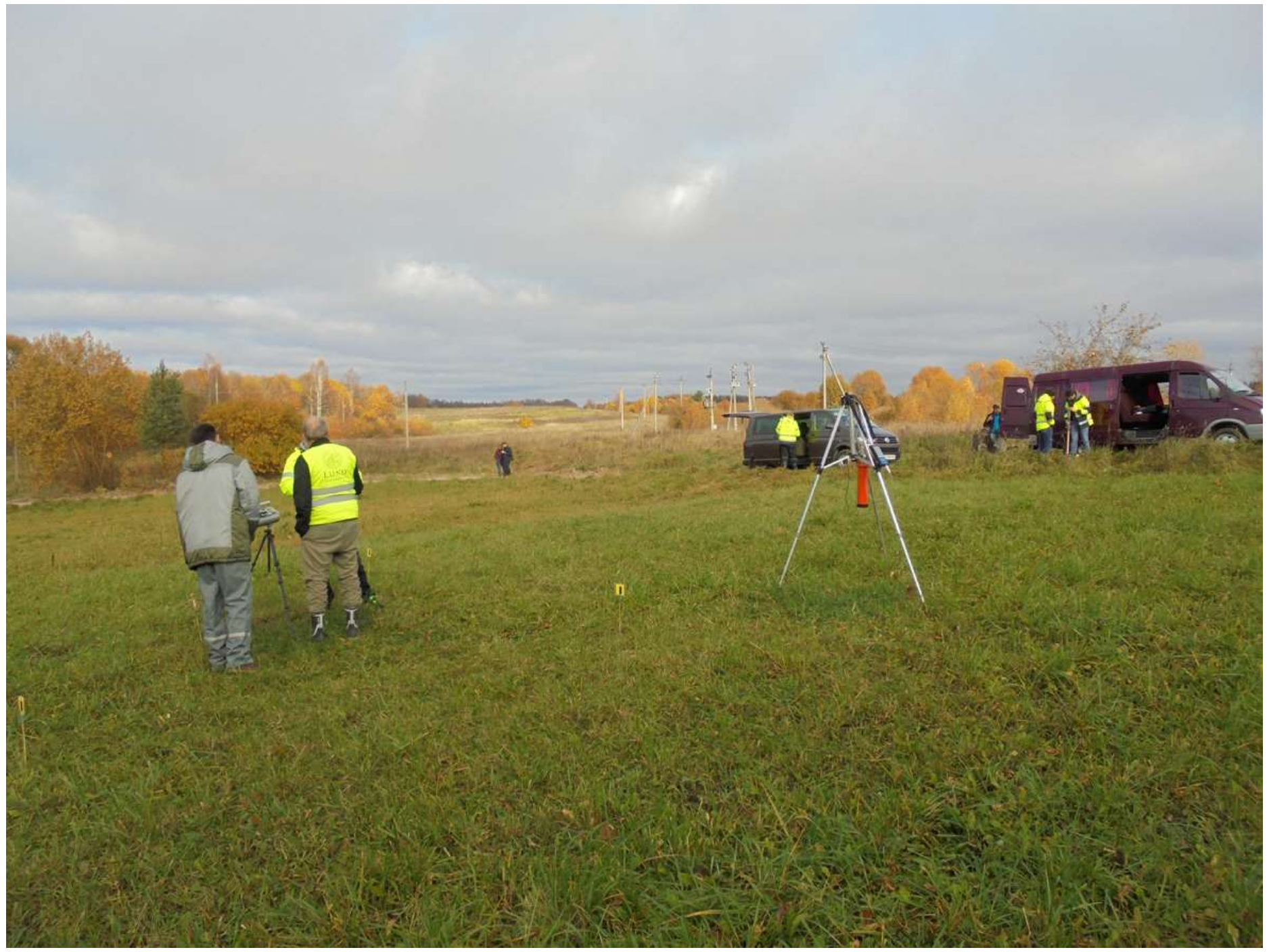

Plots surveyed by the joint expedition of Lund University, Research Institute of Radiation Hygiene and Institute of Radiobiology around BelNPP in 2019.

Barani-Plot-31. 15-10-2019.

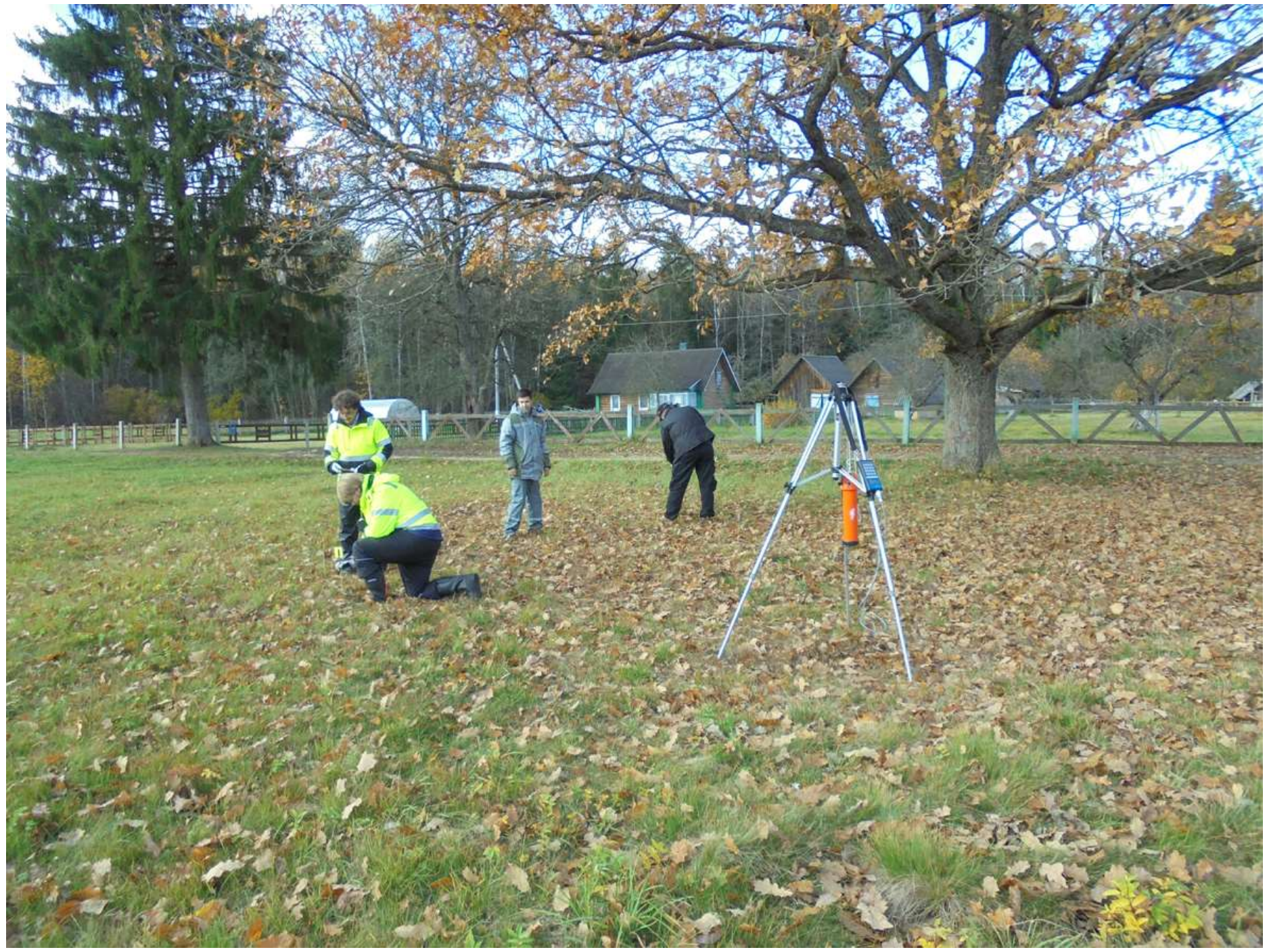

Gadiluny-Plot-32. 15-10-2019.

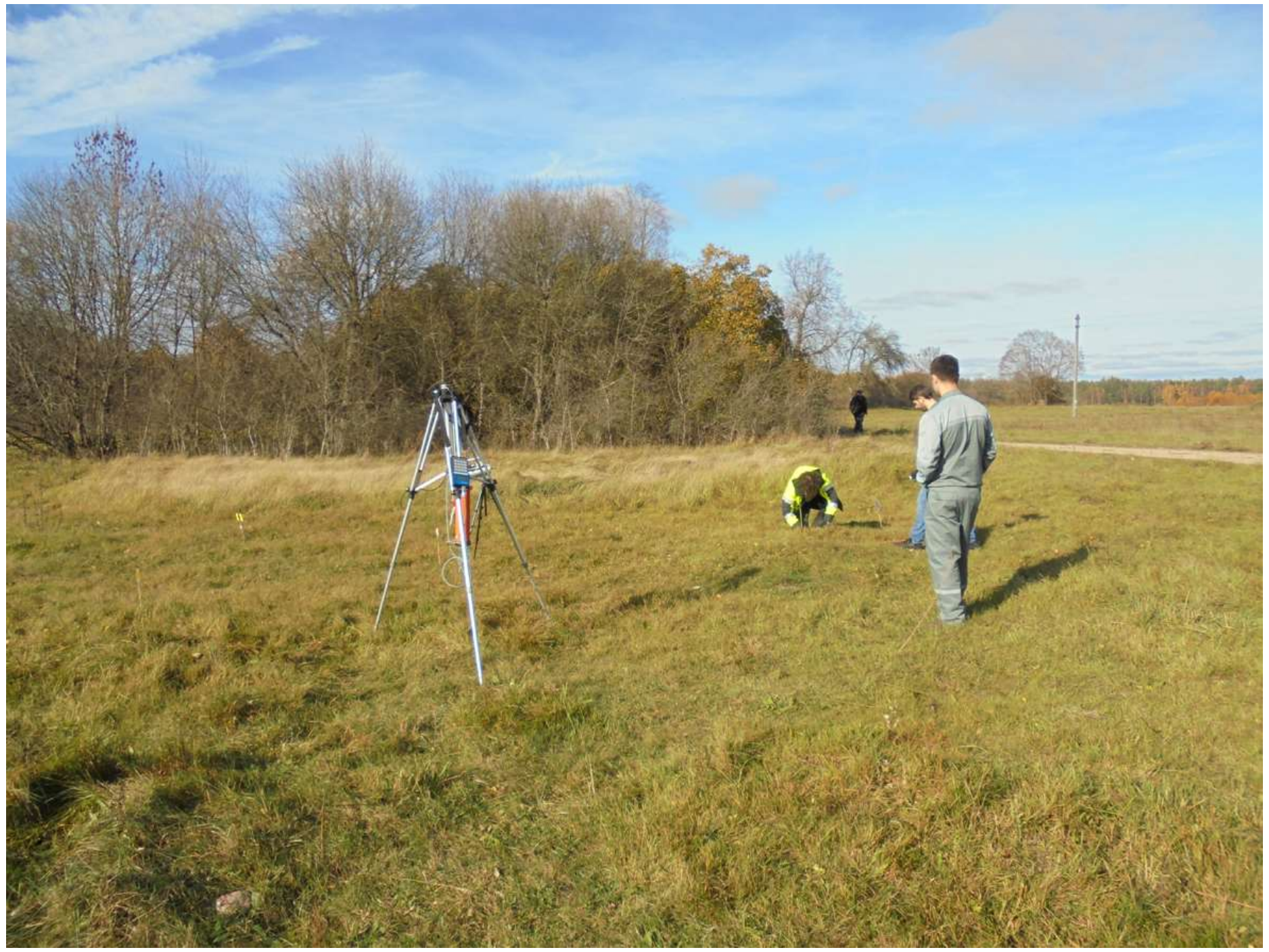

Plots surveyed by the joint expedition of Lund University, Research Institute of Radiation Hygiene and Institute of Radiobiology around BelNPP in 2019.

Podolcy-Plot-33. 15-10-2019.

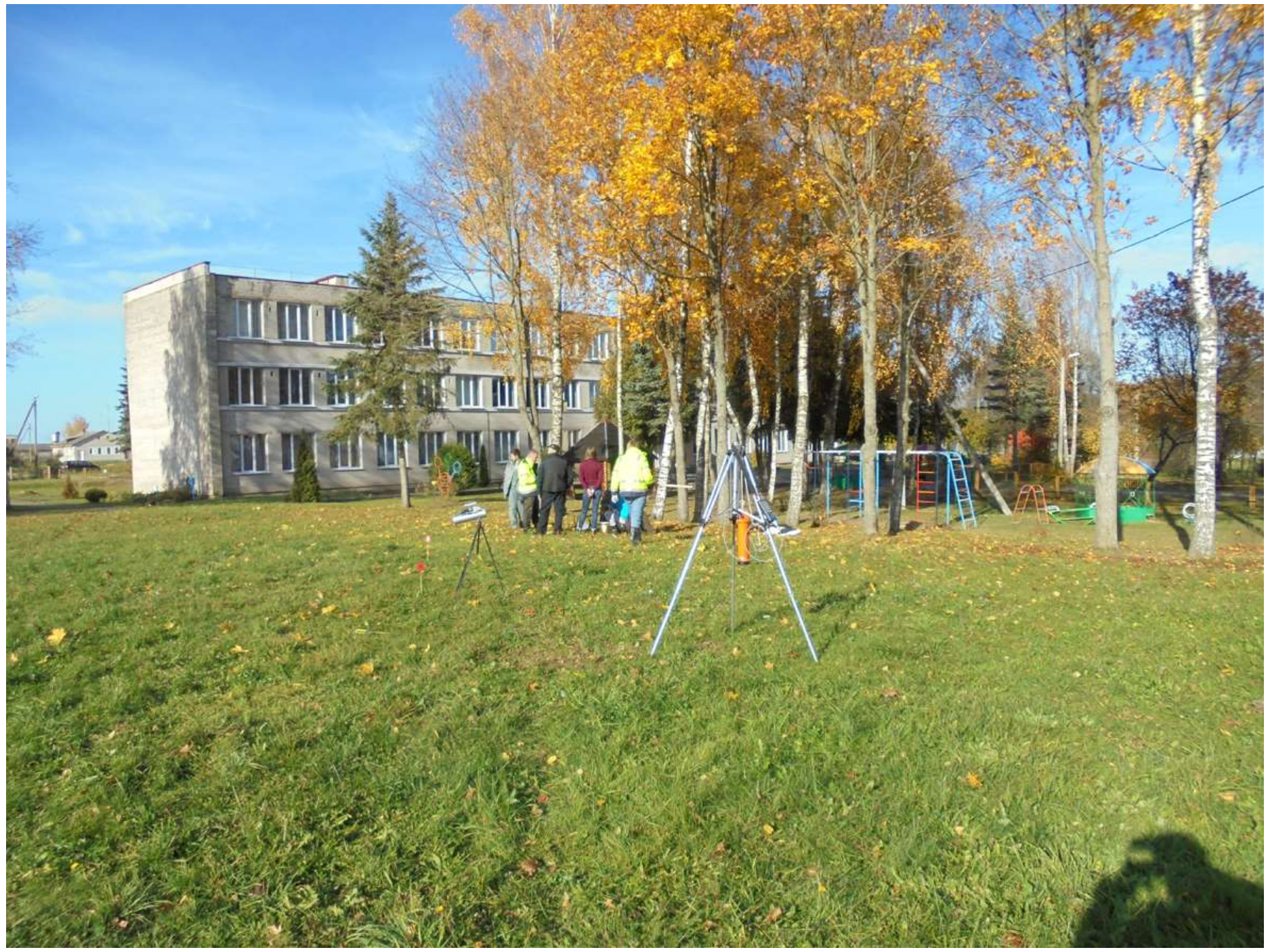

Kemelishki-Plot-34. 15-10-2019.

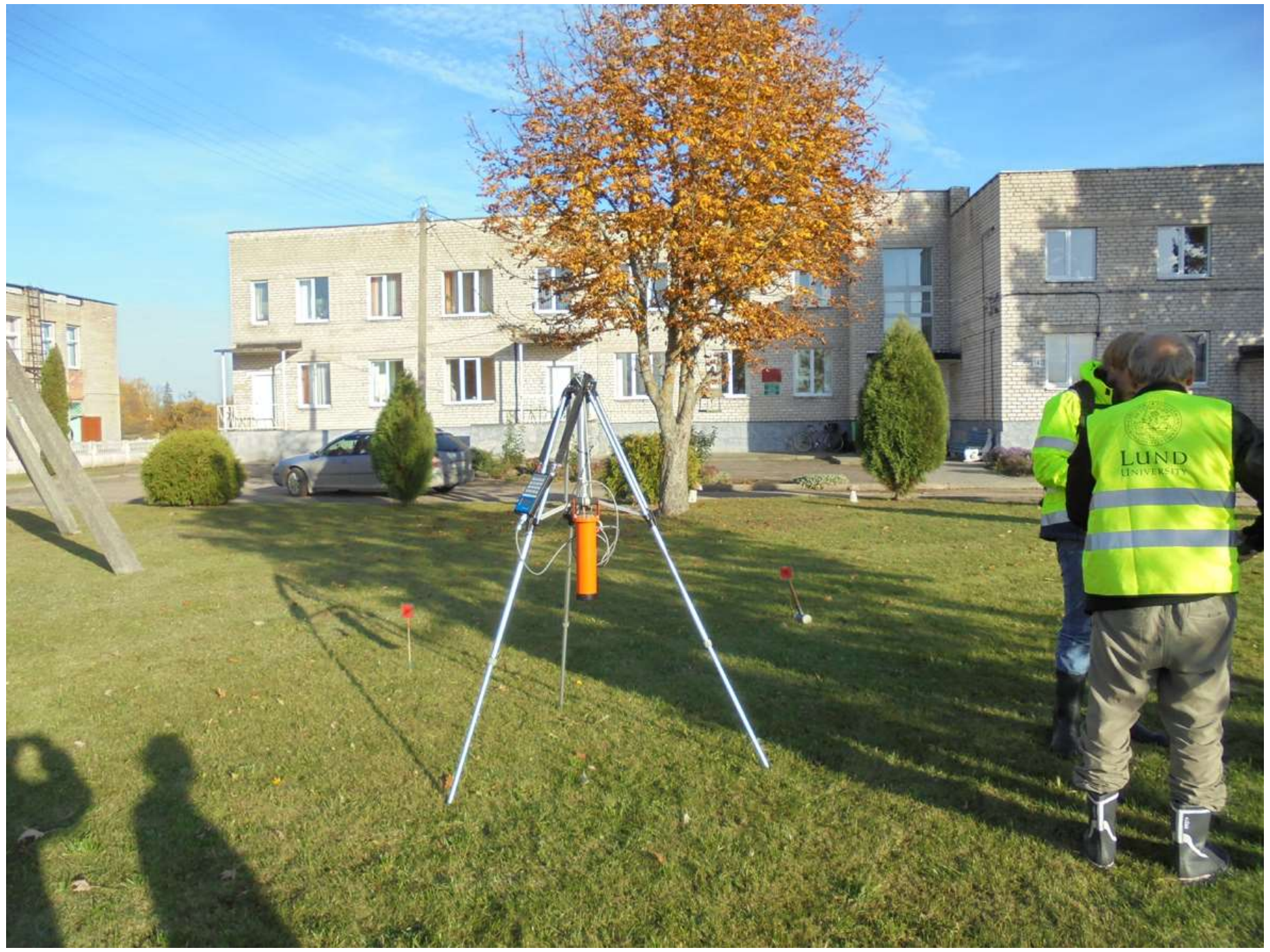

Plots surveyed by the joint expedition of Lund University, Research Institute of Radiation Hygiene and Institute of Radiobiology around BelNPP in 2019.

Rytan-Plot-35. 15-10-2019.

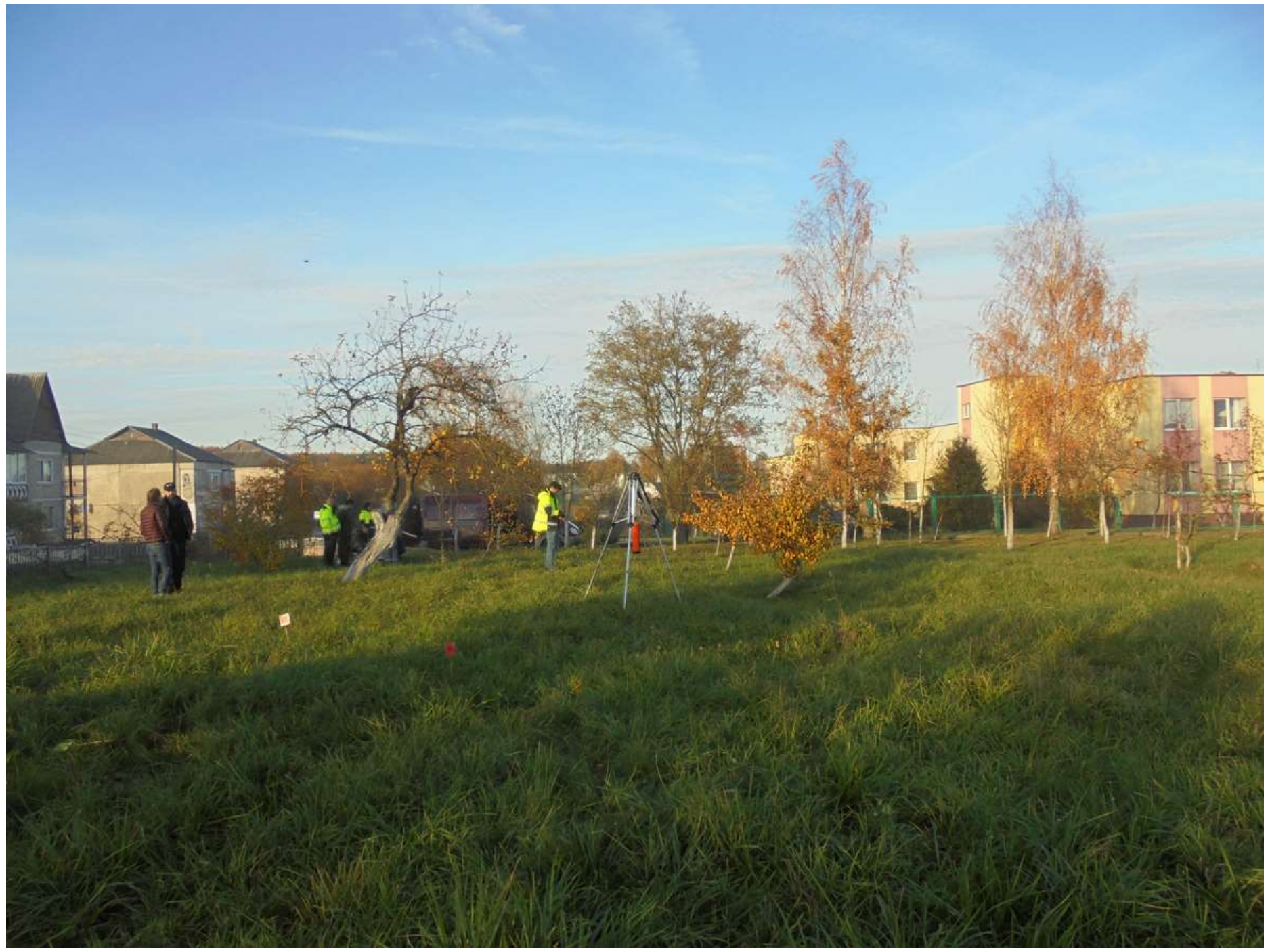

Bystrisa-Plot-36. 16-10-2019.

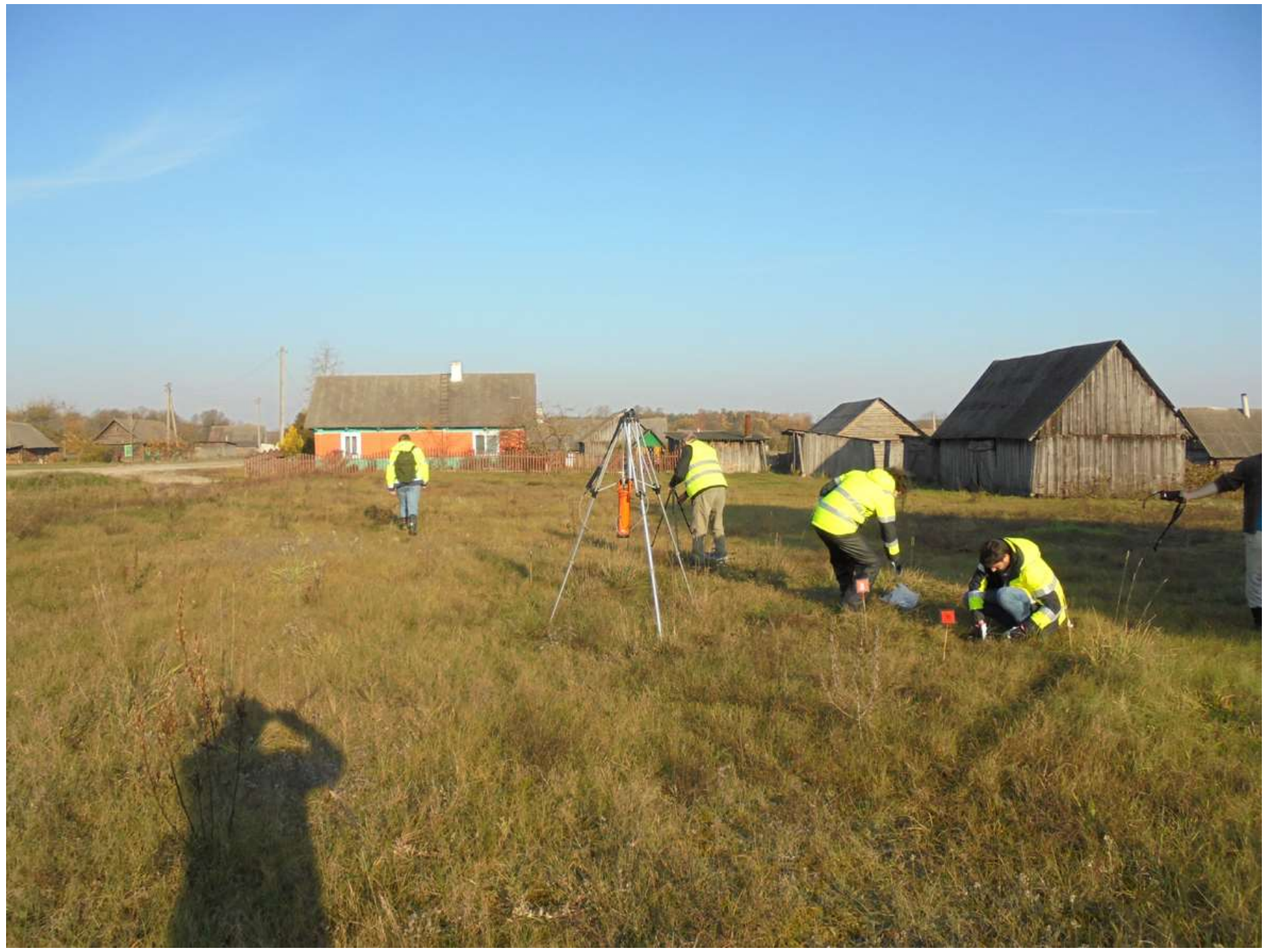

Plots surveyed by the joint expedition of Lund University, Research Institute of Radiation Hygiene and Institute of Radiobiology around BelNPP in 2019.

Zharneli-Plot-37. 16-10-2019.

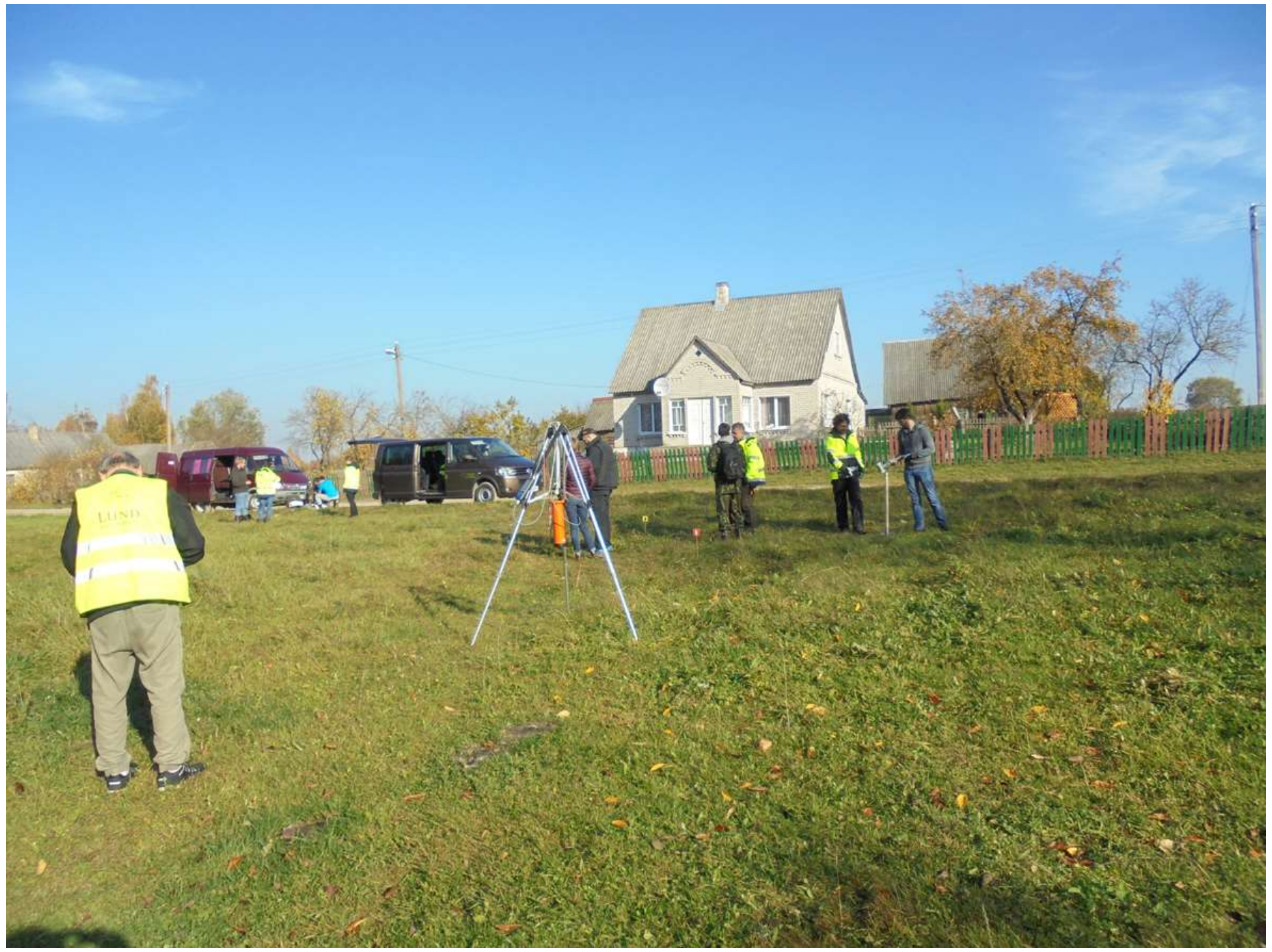

Osinovka-Plot-38. 16-10-2019.

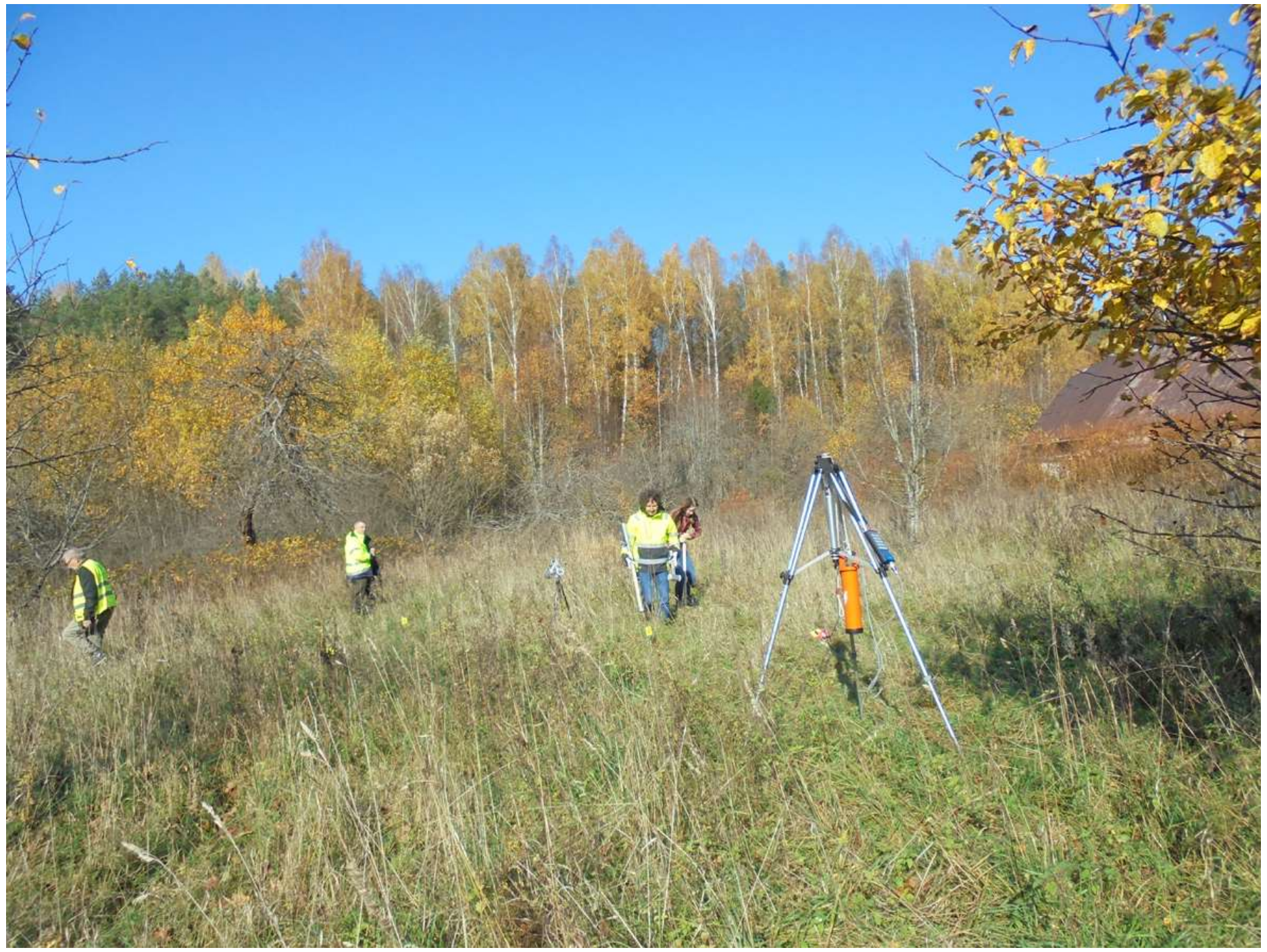

Plots surveyed by the joint expedition of Lund University, Research Institute of Radiation Hygiene and Institute of Radiobiology around BelNPP in 2019.

Zakharishki-Plot-39. 16-10-2019.

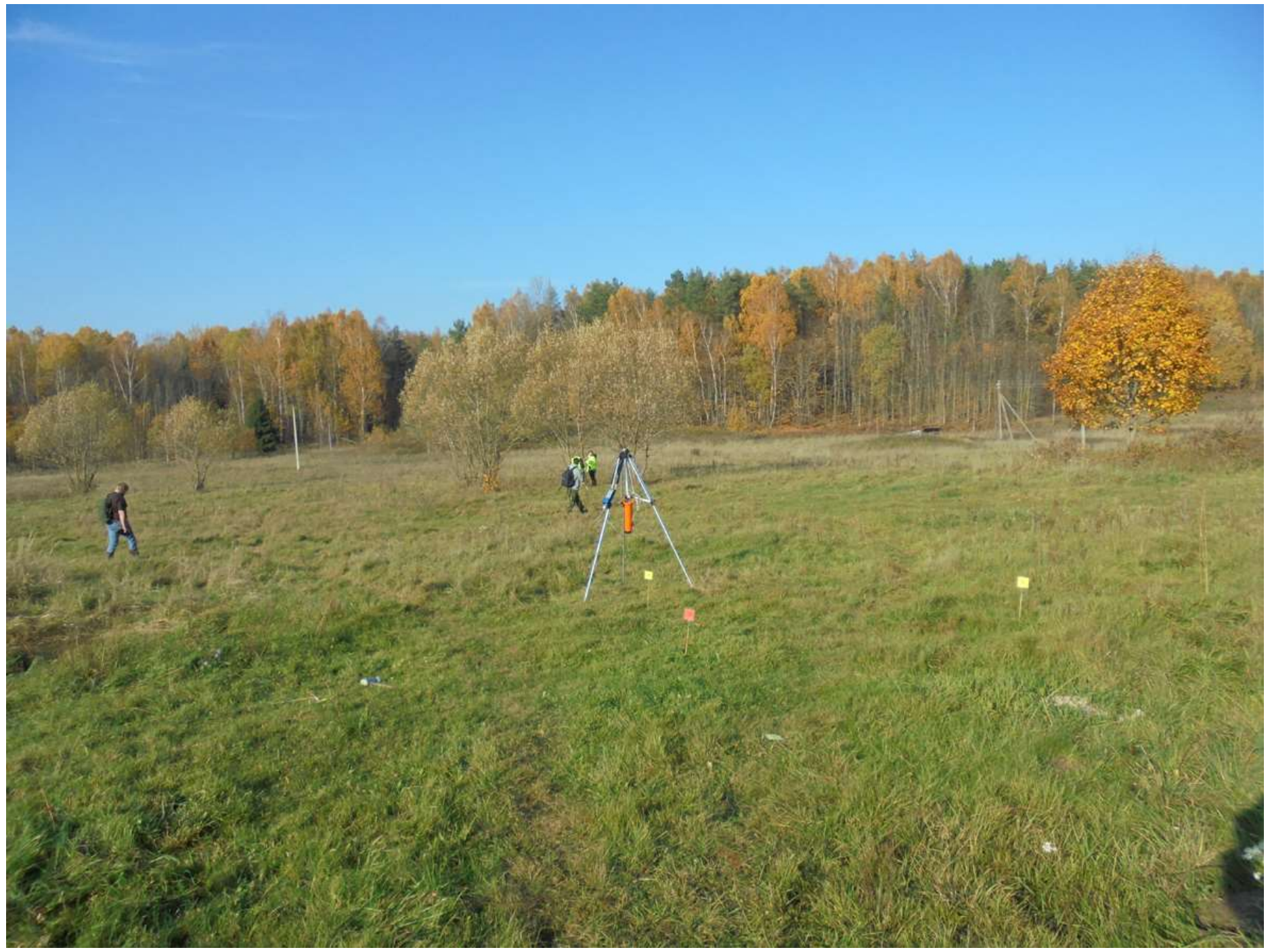

Dreveniki-Plot-40. 16-10-2019.

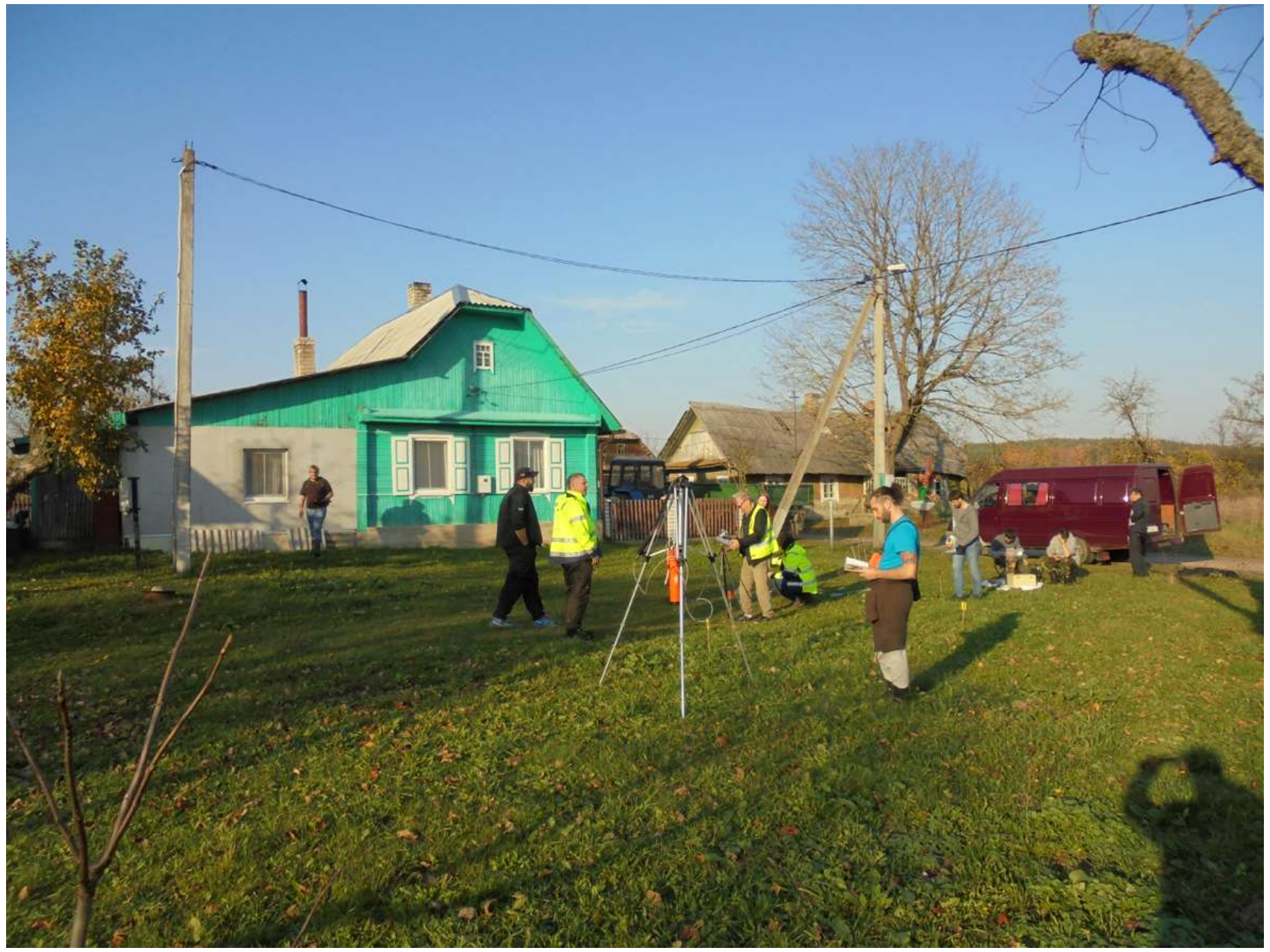

Plots surveyed by the joint expedition of Lund University, Research Institute of Radiation Hygiene and Institute of Radiobiology around BelNPP in 2019.

Radjuly-Plot-41. 17-10-2019.

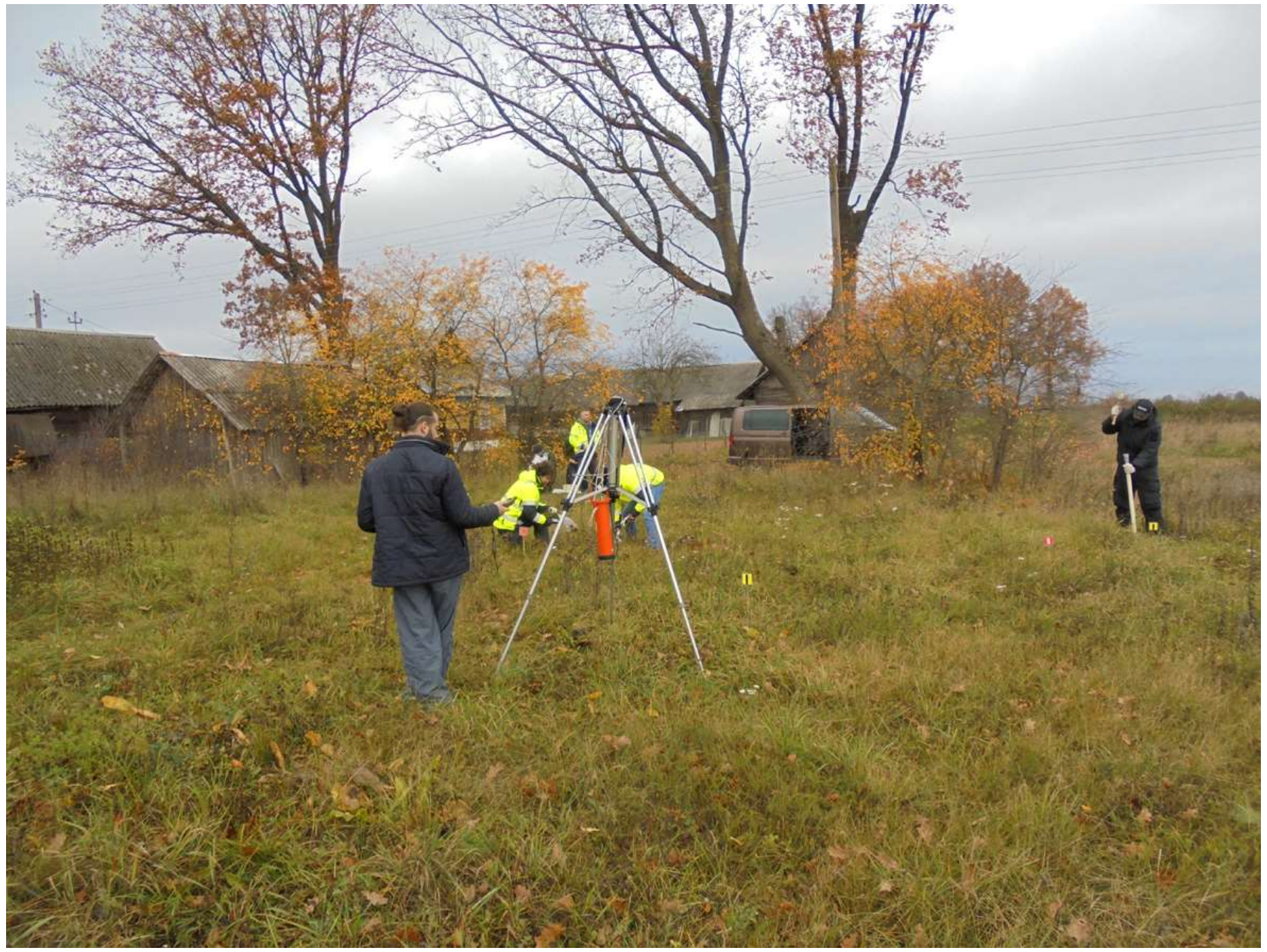

Palushi-Plot-42. 17-10-2019.

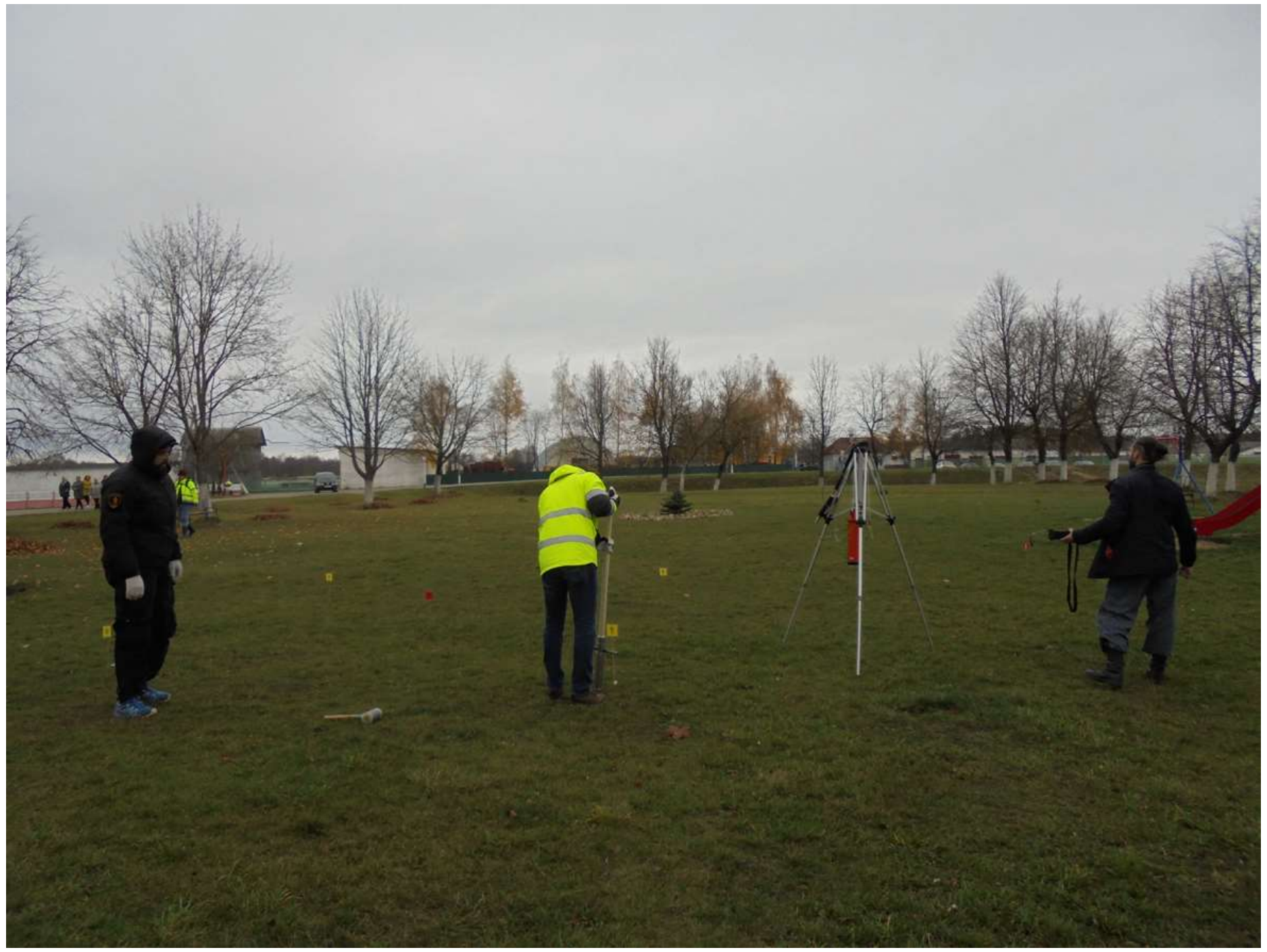

Plots surveyed by the joint expedition of Lund University, Research Institute of Radiation Hygiene and Institute of Radiobiology around BelNPP in 2019.

Grodi-Plot-43. 17-10-2019.

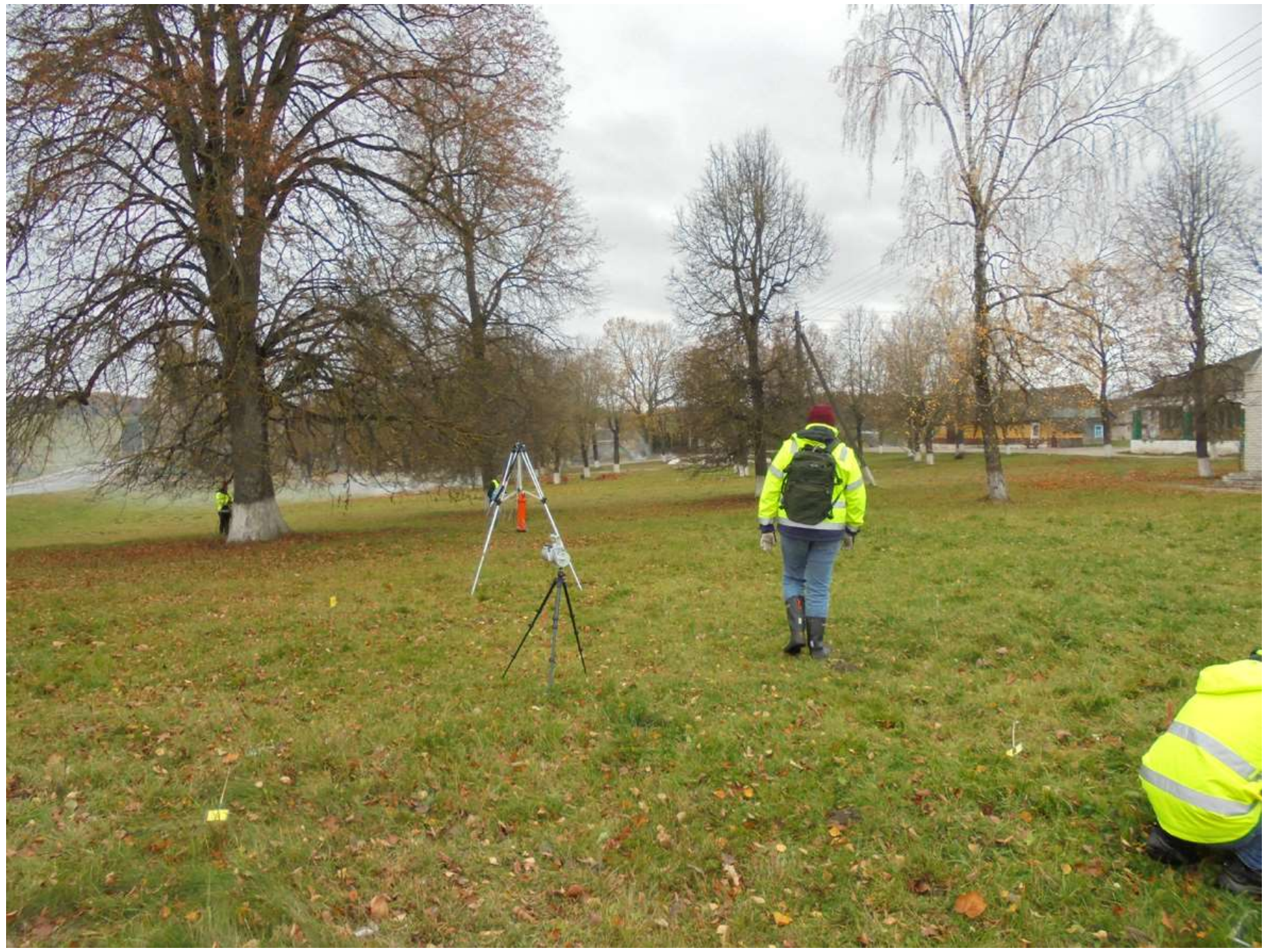

Astrovets-i-Plot-44. 17-10-2019.

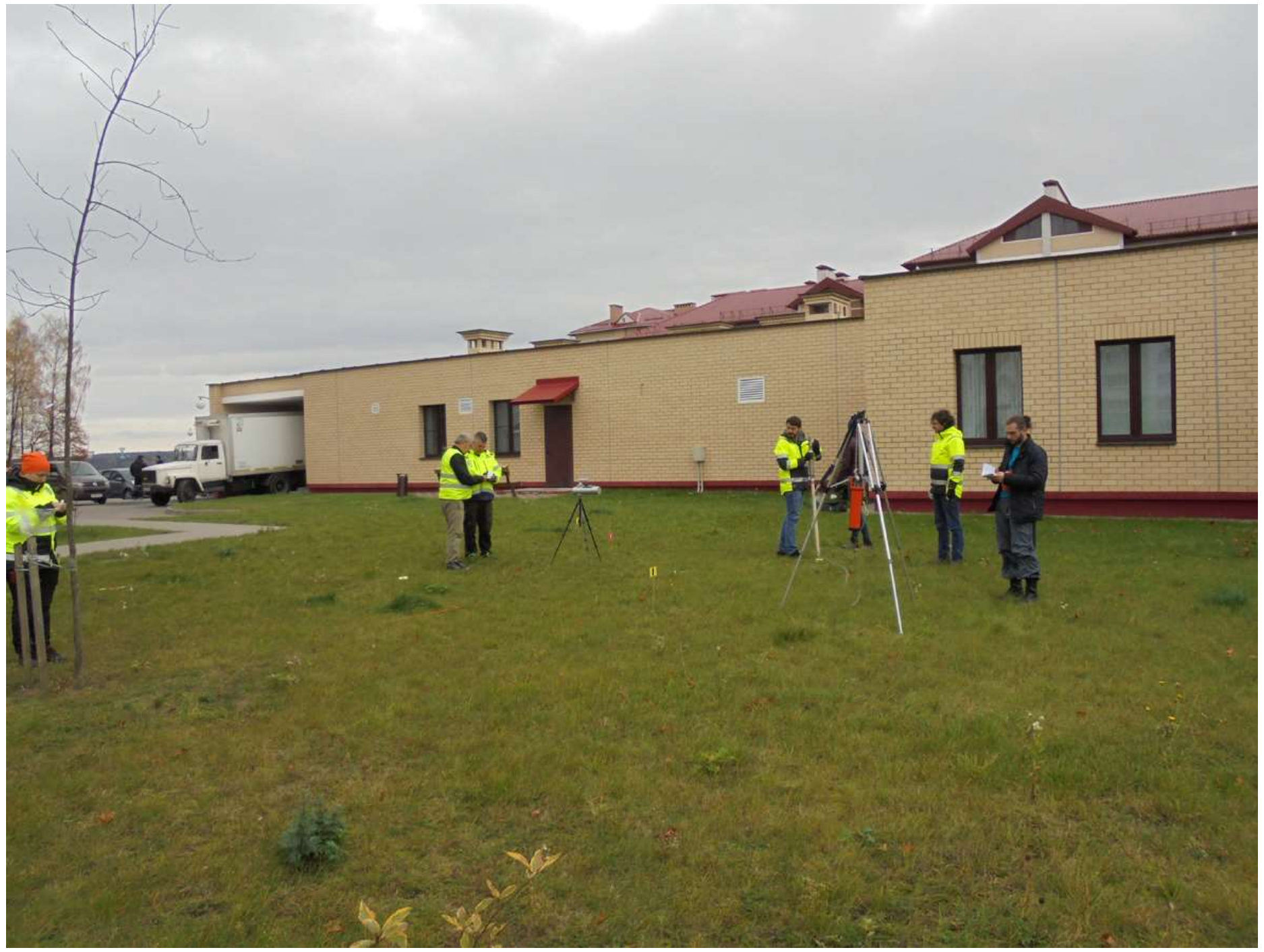

Plots surveyed by the joint expedition of Lund University, Research Institute of Radiation Hygiene and Institute of Radiobiology around BelNPP in 2019.

Astrovets-p-Plot-45. 17-10-2019.

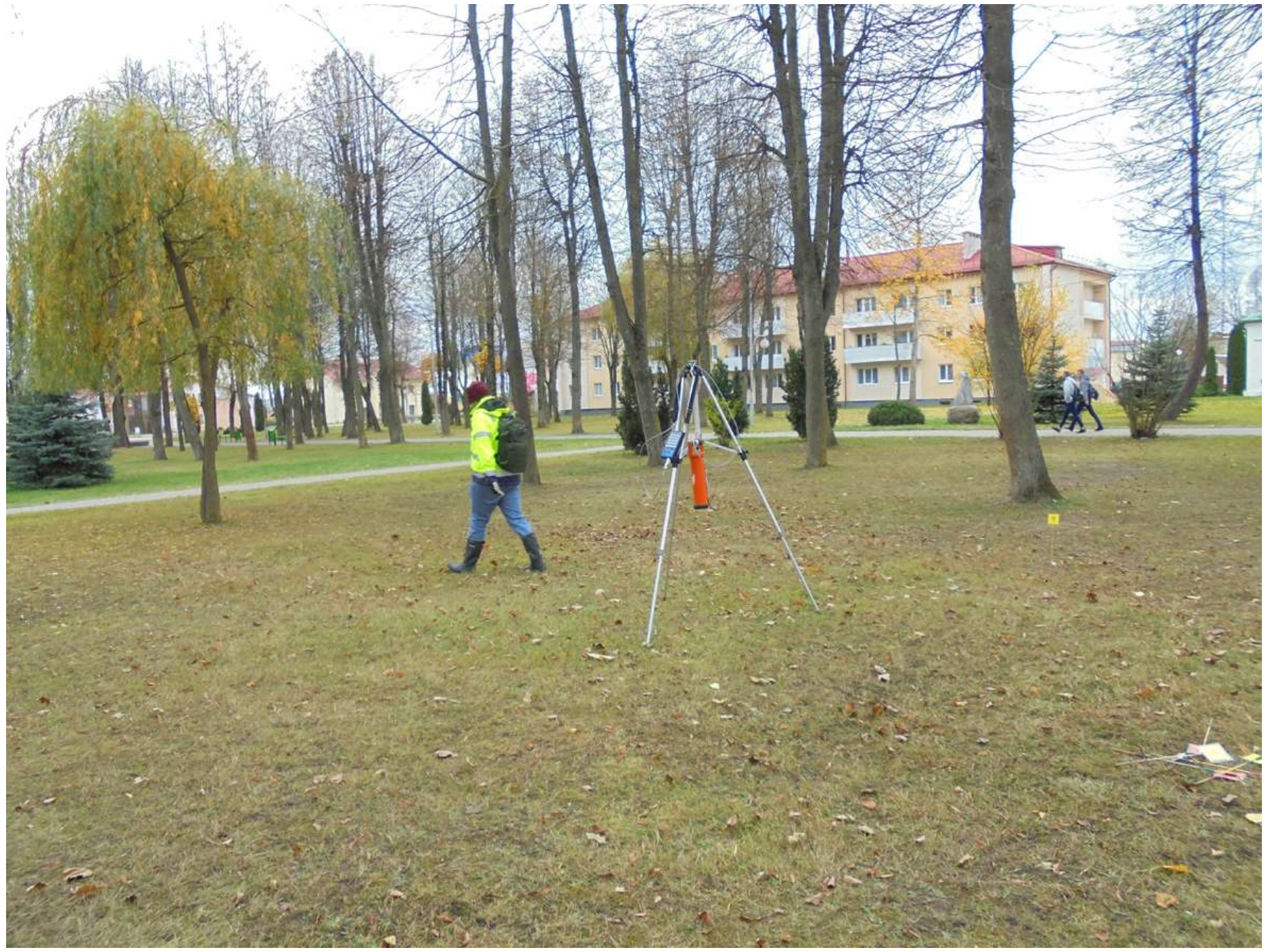

Supplement: Supplementary file 1 [file supplementary-s2-to-baseline_levels_of_137cs-bel_npp-26-05-2025_ncag063.pdf]
